# Stereodynamism in Chiral Polyaromatic Phosphepines

**DOI:** 10.1002/chem.202500343

**Published:** 2025-05-19

**Authors:** Mengling Lyu, Thomas Delouche, Réka Mokrai, Thomas Vives, Thierry Roisnel, Muriel Hissler, Zoltán Benkő, Pierre‐Antoine Bouit

**Affiliations:** ^1^ Department of Inorganic and Analytical Chemistry Budapest University of Technology and Economics Műegyetem rkp. 3 Budapest H‐1111 Hungary; ^2^ Univ Rennes CNRS ISCR–UMR 6226 Rennes F‐35000 France; ^3^ HUN‐REN‐BME Computation‐Driven Chemistry Research Group Műegyetem rkp. 3 Budapest H‐1111 Hungary

**Keywords:** DFT‐caclulations, helicenoids, luminescence, organophosphorus, stereodynamism

## Abstract

In the present experimental‐computational study, we demonstrate that the stereodynamism of phosphahelicenoids can be used to afford diastereoisomers of chirally perturbed polyaromatics fused with a phosphepine ring (seven‐membered P‐cycle). The phosphahelicenoids have been synthesized through a stereospecific approach from commercially available BINAPs. In particular, using the combination of NMR, X‐ray diffraction, and chiral HPLC experimental techniques with DFT and higher level *ab initio* calculations, we highlight a complex interplay between the inversion at the phosphorus stereocenter and the epimerization of the helicenoid framework, as well as a competition with a rearomatization reaction through P‐extrusion. The impact of the conformational modifications on the structure have been studied through X‐ray diffraction. The spectroscopic properties, in particular, of chiroptical nature, including circularly polarized luminescence, are also discussed in detail and compared to DFT and ADC(2) models.

## Graphical abstract

Stereodynamism of phosphahelicenoid scaffolds can be used to afford diastereoisomers of polyaromatics fused with a chiral seven‐membered P‐ring. In particular, a complex interplay between the inversion at the P centre and helicenoid epimerization, as well as a competition with a rearomatization reaction, takes place.

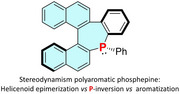

## 1 Introduction

In the last decades, π‐conjugated materials based on heteroaromatics (featuring O, N, B, Si, or P) have been widely incorporated in (opto)‐electronic devices such as organic light‐emitting diodes (OLEDs), organic photovoltaics, just to mention a few.^[^ ^1^, ^2^, ^3^, ^4^ ^]^ Among these heterocycles, organophosphorus derivatives are particularly interesting, as their structures, reactivities, and electronic properties highly depend on the nature of the P‐cycle and the valency/coordination number of the P atom.^[^ ^5^ ^]^ In recent years, a great diversity of π‐conjugated P‐cycles has been developed: four‐membered (phosphetenes),^[^ ^6^ ^]^ five‐membered (phospholes),^[^ ^7^ ^]^ six‐membered (phosphinines),^[^ ^8^ ^]^ and more recently even seven‐membered rings (phosphepines).^[^ ^9^ ^]^

**Figure 1:**
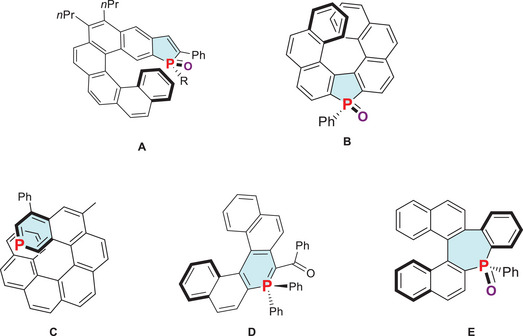
Examples of phospholes **A‐B**, phosphinines **C‐D** and a phosphepine **E** fused to helicene/helicenoid backbone.

Meanwhile, in the field of π‐conjugated systems, helicenes and helicenoids have also emerged as fascinating chiral platforms.^[^ ^10^ ^]^ These organic compounds consist of *ortho*‐fused aromatics, and their helical arrangement confers them chiroptical properties. Thus, it is not surprising that the combination of these two appealing fields namely, organophosphorus and helicene chemistry, may afford unprecedented chiral heteroaromatic structures. Interestingly, the nature of the P‐heterocycle has a strong influence on the stereodynamic properties of the so‐called phospha‐helicenes as, contrary to most of the other P‐heterocycles, P‐inversion in phospholes may occur at room temperature because of an aromatic transition state.^[^ ^11^ ^]^ In addition to all the properties previously described, phospha‐helicenes also display attractive properties in the field of enantioselective catalysis.^[^ ^12^ ^]^ The first examples of organophosphorus helicenes were carbo[n]helicenes exocyclicly‐substituted by P‐derivatives.^[^ ^13^ ^]^ However, in order to maximize π‐conjugation, most of the effort has been devoted to fusing the P‐ring to the helicene backbone, either as a terminal ring or within the helical backbone. Over the last 15 years, various helicenes containing the phosphole ring were prepared (**A‐B**, Figure 1) and some of them were used as ligands for enantioselective catalysis or emitters in fluorescent OLEDs.^[^ ^14^ ^]^ Regarding the other P‐rings, the number of structures described so far is significantly more limited. Beránek et al recently reported a phospha[7]helicene (**C**, Figure 1) featuring a terminal phosphinine ring.^[^ ^15^ ^]^ Lately, we also reported a phospha[5]helicene with a central six‐membered P‐ring possessing an internal ylidic bond (**D**, Figure 1).^[^ ^16^ ^]^ Finally, our groups previously reported the stereospecific synthesis of a phospha[5]helicenoid exhibiting a phosphepine ring (**E**, Figure 1).^[^ ^17^ ^]^ Contrary to many other heterohelicenes, these last derivatives are easily prepared in two steps from commercially available compounds. In the present work we scrutinize the stereodynamic properties of helicenoids containing seven‐membered P‐rings and show that the conformational flexibility of a trivalent phosphine can afford diastereoisomers. The impact of the conformation on the structural and the chiroptical properties is also presented.

## 2 Results and Discussion

### 2.1 Synthesis

As previously reported, the enantiopure phosphepine‐fused [5]helicenoids **1** and **2** are easily prepared by nucleophilic aromatic substitution on the oxidized form of 2,2′‐bis(diphenylphosphino)‐1,1′‐binaphthalene (BINAPO).^[^ ^17^ ^]^ The phosphepine oxide **1** and its reduced form **2** exhibit three types of chirality, leading to eight possible isomers (four pairs of enantiomers): a helical chirality at the [5]helicene, a central at P, and another helical at the benzo unit fused to the seven‐membered ring. This can be indicated by the corresponding notation for the enantiomer pair (*M*, R_P_, *P*)‐**1**/(*P*, S_P_, *M*)‐**1** (the order of the descriptors is the same as above). Our computational studies (see below) have outlined that the two kinds of helical chiralities are interconnected (flipping of the [5]helicene triggers the epimerization at the benzo unit in the case of the experimentally accessed isomers), and therefore, in the following the chirality at the [5]helicene backbone will only be indicated followed by the central chirality at P, such as (*M*,R_P_)‐**1**/(*P*,S_P_)‐**1**.

**Scheme 1:**
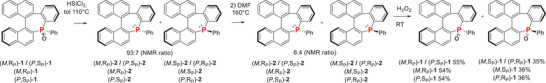
Sequence of reduction, partial P‐inversion, and oxidation applied to (*M*,R_P_)‐**1**/(*P*, S_P_)‐**1**.

**Figure 2:**
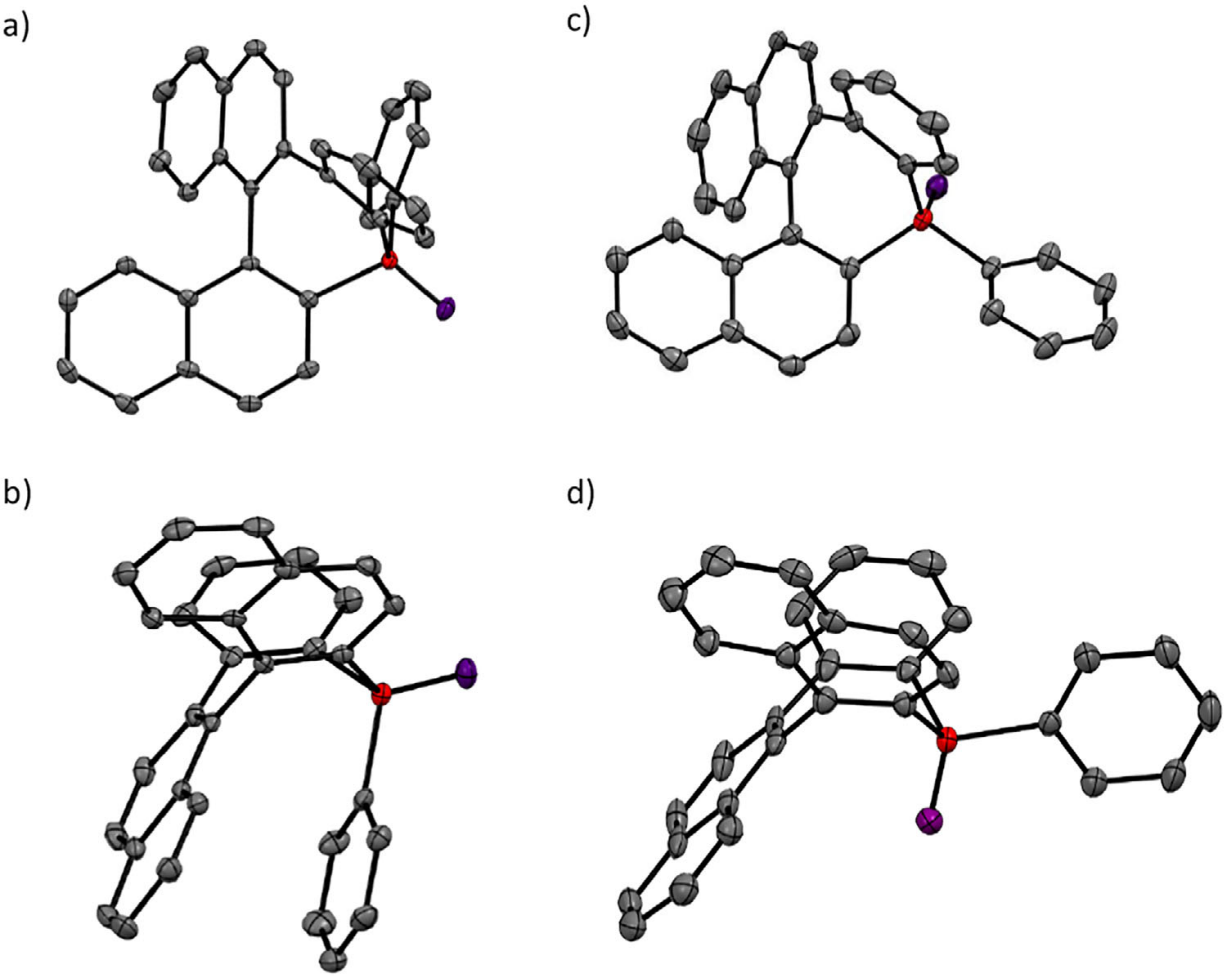
Top and side view of X‐ray crystallographic structure of (a, b) (*M*,R_P_)‐**1** ^[^ ^17^ ^]^ and (c, d) (*M*,S_P_)‐**1**.

While studying the reduction of the racemic σ^4^,λ^5^ phosphine oxide (*M*,R_P_)‐**1**/(*P*,S_P_)‐**1** to its σ^3^,λ^3^ analog (*M*,R_P_)‐**2**/(*P*,S_P_)‐**2** (Scheme 1) using HSiCl_3_, we noticed a small signal for a byproduct (at δ = −18 ppm) in the ^31^P NMR spectrum of the crude product. This by‐product was not observed after purification and thus could not be characterized (Figure S4). Assuming that this by‐product could be a diastereoisomer originating from the pyramidal inversion at the trivalent P‐center, we applied harsher thermal conditions to the trivalent derivative. By heating overnight the racemic (*M*,R_P_)‐**2**/(*P*,S_P_)‐**2** in DMF at 160 °C (Scheme 1), we observed that the intensity of the ^31^P NMR signal at = −18 ppm increased so that it approaches that of the starting material (NMR ratio 60:40, Figure S5). After oxidizing the mixture and separating the compounds by column chromatography, we obtained, besides the initial derivative (*M*,R_P_)‐**1**/(*P*,S_P_)‐**1**, a so far unknown compound. This new derivative was fully characterized by multinuclear NMR spectroscopy as well as MS analysis and its structure was unambiguously assigned by single crystal X‐ray diffraction to the racemic mixture (*M*,S_P_)‐**1**/(*P*,R_P_)‐**1**. As represented in Figure 2, (*M*,S_P_)‐**1**/(*P*,R_P_)‐**1** is indeed the diastereoisomer of (*M*,R_P_)‐**1**/(*P*,S_P_)‐**1** originating from the pyramidal inversion of the trivalent phosphine **2**. Chiral HPLC confirmed that, in agreement with the X‐ray structure (vide infra), the reaction indeed delivers a racemic mixture of the so far unprecedented diastereoisomer (*M*,S_P_)‐**1**/(*P*,R_P_)‐**1**. Using the enantiopure form of (*M*,R_P_)‐**1** (respectively (*P*,S_P_)‐**1**), the sequence produces the enantiopure (*M*,S_P_)‐**1** (respectively (*P*,R_P_)‐**1**), as confirmed by X‐ray diffraction and chiral HPLC (see Supporting Information).

**Figure 3:**
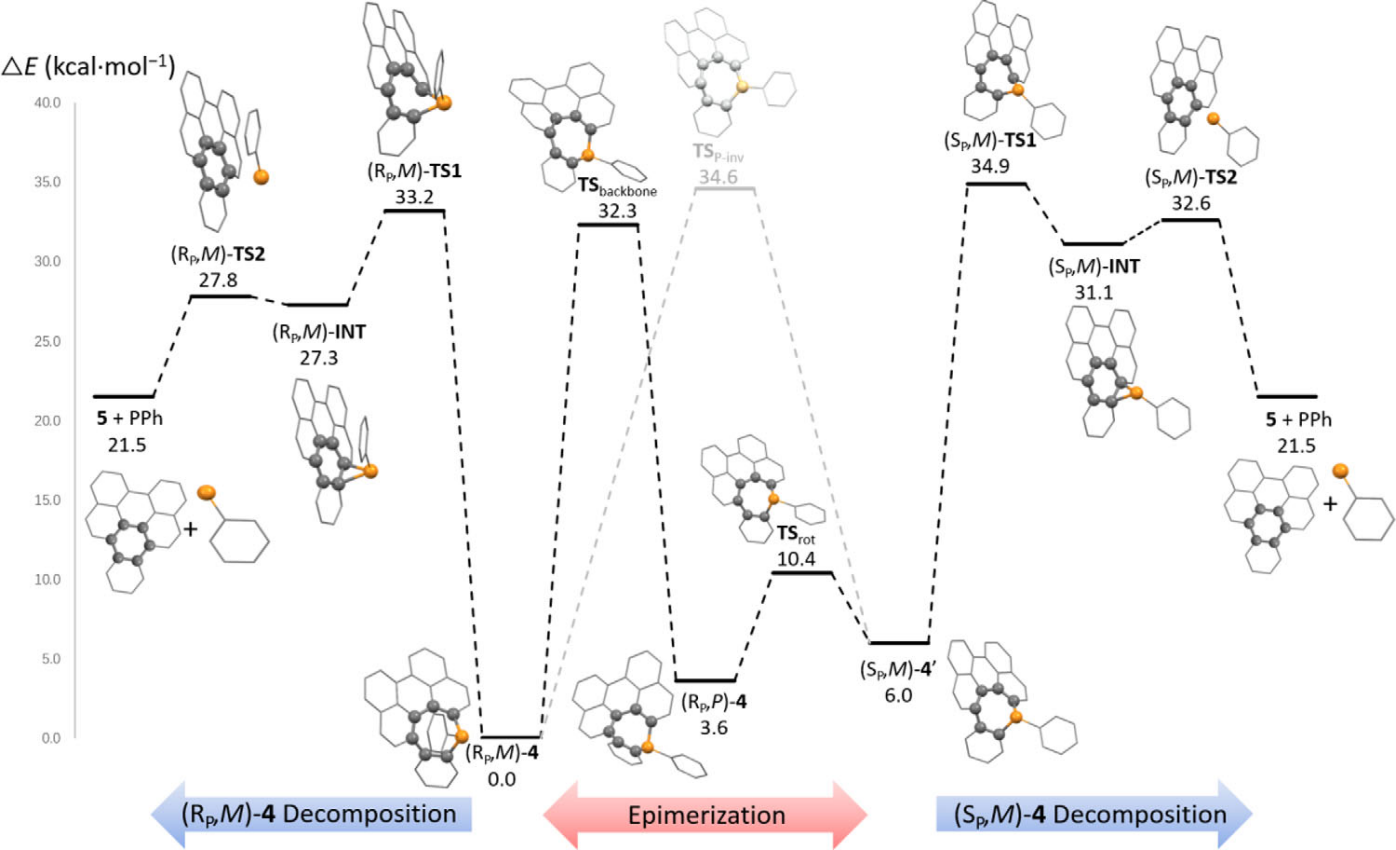
Relative energy profile for the epimerization of phosphine **4** and its decomposition to **5** at the B3LYP‐D3/cc‐pVDZ level.

The molecular structures of the new derivatives (*M*,S_P_)‐**1**/(*P*,R_P_)‐**1** were determined by single‐crystal X‐ray diffraction (Figure 3, Tables S1,S2, and Figures S7–S9)^[^ ^18^ ^]^ and the structure of this helicenoid can be compared to that of its previously reported diastereoisomer (*M*,R_P_)‐**1**/(*P*,S_P_)‐**1**.^[^ ^17^ ^]^ The phosphepine ring exhibits an expected boat‐like conformation with the oxygen on the P‐atom in axial position (Figure 2c,d), which confirms that the configuration of the P center is reversed compared to the previously reported diastereoisomer with the O substituent in equatorial position (Figure 2a,b). The main consequence of the inversion at the P center is the absence of intramolecular π‐stacking between the equatorial P‐phenyl and one of the naphthyl fragments in (*M*,S_P_)‐**1**. Such geometry hints at the lower conformational stability of this new diastereoisomer (vide infra). The “helicity” angle of (*M*,S_P_)‐**1**/(*P*,R_P_)‐**1** is 73.9° and 66.9°, lying in the same order of magnitude as the previously described derivative (∼68°).^[^ ^17^ ^]^ In general, the other metric parameters of (*M*,S_P_)‐**1**/(*P*,R_P_)‐**1** are similar to those of (*M*,R_P_)‐**1**/(*P*,S_P_)‐**1** with alternating single C─C and double C ═C bonds in the seven‐memberd ring (1.473(5) to 1.494(4) Å and 1.387(5) to 1.414(5) Å, respectively) and similar P─C distances (1.803(3) and 1.810(3) Å). The C─P─C angle of 99.65(17) in the phosphepine ring is somewhat smaller than the ideal angle in a tetrahedral coordination, in line with the crowded geometry due to the large ring size. The structures of the two diastereomers of phosphepine oxide **1** optimized with DFT methods are in good agreement with those obtained experimentally by X‐ray crystallography (Table S2). These observations on the geometries outline that P‐inversion allows the preparation of further chiral heteroaromatic derivatives.

**Scheme 2:**
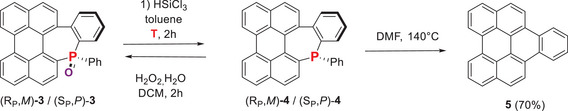
Tests of P‐inversion on a racemic mixture of (R_P_,*M*)‐**3**/(S_P_,*P*)‐**3**.

The so‐called Scholl reaction is an efficient tool to access fused ring systems as it was demonstrated previously for the transformation of the helicenoid **1** to the perylenoid **3**.^[^ ^17^ ^]^ After synthesizing the new diastereoisomer couple of **1**, we hypothesised that the so far unknown diastereomer (S_P_,*M*)‐**3**/(R_P_,*P*)‐**3** could also be obtained in a similar way. Note that during the Scholl reaction, one of the three chiralities, namely the helical at the [5]helicene part, is lost. For the (R_P_,*M*)‐**3**/(S_P_,*P*)‐**3** couple, the helical chirality *M* or *P* (behind the descriptor of the central chirality) refers to the benzo unit at the phosphepine.

First we attempted to synthesize (S_P_,*M*)‐**3**/(R_P_,*P*)‐**3** applying the “reduction‐epimerization‐oxidation” sequence (Scheme 2). When the racemic mixture of (R_P_,*M*)‐**3**/(S_P_,*P*)‐**3** was reduced in the same conditions as above, a main P‐derivative could be assigned to the trivalent phosphine (R_P_,*M*)‐**4**/(S_P_,*P*)‐**4** according to the ^31^P NMR spectrum. Although a minor ^31^P NMR signal at −19.1 ppm was observed in the crude mixture containing the reduction products, potentially attributable to (S_P_,*M*)‐**4**/(R_P_,*P*)‐**4** (see Figure S6), we could not isolate this compound. After reoxidation, (R_P_,*M*)‐**3**/(S_P_,*P*)‐**3** could only be isolated, as initially observed with scaffold **1**, and the targeted epimerization was not achieved. In order to force the inversion at the P‐atom, we applied a similar thermal treatment to (R_P_,*M*)‐**4**/(S_P_,*P*)‐**4** as described above. To our surprise, upon heating the mixture to 140 °C, we observed the formation of naphtho[1,2,3,4‐ghi]perylene **5** (70% yield) (Scheme 2),^[^ ^19^ ^]^ generated through the extrusion of a PhP fragment from the phosphepine ring.^[^ ^20^ ^]^ Attempts to trap the elusive phosphinidene intermediate with dimethyl acetylenedicarboxylate and observe it by ^31^P NMR failed. Nevertheless, this undesired decomposition reaction may be used in the future to generate novel all‐carbon polyaromatics, as previously observed with polyaromatic phospholes or chalcogen‐containing derivatives.^[^ ^21^ ^]^

**Scheme 3:**
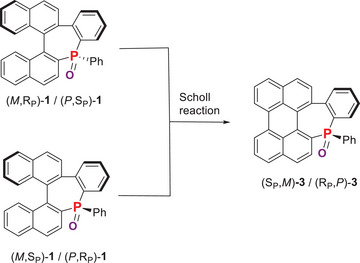
“Scholl” conditions ((1) AlCl_3_/NaCl, 140 °C, 2.5 hours, (2) DDQ, Tol, 60 °C, 2.5 hours) applied to (*M*,R_P_)‐**1**/(*P*,S_P_)‐**1** and (*M*,S_P_)‐**1**/(*P*,R_P_)‐**1**.

Second, in order to isolate the hypothetical diastereoisomer (S_P_,*M*)‐**3**/(R_P_,*P*)‐**3**, we decided to apply our optimized Scholl conditions to the racemic mixture of the new derivative (*M*,S_P_)‐**1**/(*P*,R_P_)‐**1**. However, in these conditions, the reaction afforded again racemic (R_P_,*M*)‐**3**/(S_P_,*P*)‐**3**, as it was the case for (*M*,R_P_)‐**1**/(*P*,S_P_)‐**1** (Scheme 3).^[^ ^17^ ^]^ These experimental observations triggered us to perform quantum‐chemical calculations for an in‐depth understanding.

### 2.2 Computational Considerations on the Mechanistic Aspects

In order to gain insights into the structural changes upon the transformations, and to suggest possible reaction mechanisms, we carried out computations at the B3LYP‐D3/cc‐pVDZ level of theory. To verify the DFT results, single‐point energy calculations were performed at the LNO‐CCSD(T)/cc‐pVTZ level, and all these results are in good general agreement. In the following, we will primarily discuss the energies obtained using the coupled cluster method. Besides determining the relative energies of possible isomers, we also scrutinized the transition states (and activation barriers) for the transformations of these isomers into each other.

As the consequence of the three types of chirality, for compounds **1** and **2** altogether four pairs of enantiomers can be considered in theory, and we attempted to locate all of these isomers on the potential energy surface. While for compound **2** all of the four pairs could be determined, for compound **1** three pairs were only found. Importantly, for both compounds two pairs of enantiomers have significantly (by more than 30 kcal·mol^−1^) lower relative energies compared to the others, and exactly these lower energy pairs were obtained in the experiments as well. The geometries of the lower and higher energy isomers show marked differences (see Figure S16): the aryl groups at the seven‐membered ring take up a “butterfly” orientation in the former, while they are arranged in a “twisted” form in the latter. The stability of the butterfly orientation stems from the conformational preference of the phosphepine ring (for the model tribenzo[*b,d,f*]phosphepine the twisted isomer is not even accessible computationally as all the optimization runs led to the isomer with butterfly structure). Due to the enormous energy differences between the lower and higher energy diastereomers, in the following we will only concentrate on the two enantiomer pairs lying at lower relative energy.

The relative energy of the racemate (*M*,S_P_)‐**1**/(*P*,R_P_)‐**1** is substantially, 7.3 kcal·mol^−1^, higher than that of (*M*,R_P_)‐**1**/(*P*,S_P_)‐**1**, which is consistent with the absence of intramolecular π‐stacking between the equatorial P‐phenyl group and one of the naphthyl fragments in (*M*,S_P_)‐**1**/(*P*,R_P_)‐**1**. We examined the possibility of epimerization between the lower energy diastereomers of the oxidized compound **1**. The activation barrier for the flipping of the helical backbone leading from (*M*,R_P_)‐**1**/(*P*,S_P_)‐**1** to (*M*,S_P_)‐**1**/(*P*,R_P_)‐**1** is enormous, 45.0 kcal·mol^−1^ (Scheme 3, Table 1), in line with the experimental observation that this transformation does not happen even at high temperature. We tested experimentally the possibility of the reverse reaction from (*M*,S_P_)‐**1**/(*P*,R_P_)‐**1** to (*M*,R_P_)‐**1**/(*P*,S_P_)‐**1** exhibiting a considerably smaller activation barrier of 37.7 kcal·mol^−1^. Upon heating the sample of the less stable isomer (*M*,S_P_)‐**1**/(*P*,R_P_)‐**1** overnight at 160 °C, then 4 more hours at 180 °C in DMSO, however, no transformation was observed. We also calculated the activation barrier using a solvent model for DMSO, giving 39.5 kcal·mol^−1^, explaining that the epimerization is also hindered in the reverse direction (toward the more stable isomer).

**Table 1:** Epimerization barriers of (*M*,R_P_)‐**1**, (*M*,R_P_)‐**2**, (R_P_, *M*)‐**3**, (R_P_, *M*)‐**4**, and the reaction energies for the interconversion of the two diastereomers at the LNO‐CCSD(T)/cc‐pVTZ//B3LYP‐D3/cc‐pVDZ level (values in parenthesis: B3LYP‐D3/cc‐pVDZ results).

	Type of Inversion	ΔE^‡^ kcal·mol^−1^ CCSD(T) (DFT)	ΔE_rxn_ kcal·mol^−1^ CCSD(T) (DFT)
(*M*,R_P_)‐**1** → (*P*,R_P_)‐**1**	[5]Helicene backbone	45.0 (46.2)	7.3 (7.9)
(*M*,R_P_)‐**2** → (*P*,R_P_)‐**2**	[5]Helicene backbone	44.8 (45.9)	3.0 (3.0)
(*M*,R_P_)‐**2** → (*M*,S_P_)‐**2**	P‐center pyramidal	33.1 (30.7)	3.0 (3.0)
(R_P_,*M*)‐**3** → (R_P_,*P*)‐**3**	Phosphepine backbone	31.8 (32.6)	6.8 (7.5)
(R_P_,*M*)‐**4** → (R_P_,*P*)‐**4**	Phosphepine backbone	32.3 (32.3)	3.6 (3.6)
(R_P_,*M*)‐**4** → (S_P_,*M*)‐**4**	P‐center pyramidal	36.8 (34.6)	3.6 (3.6)

In contrast to **1**, the reduced phosphepine **2** may undergo two kinds of epimerization, at least in theory; therefore, both the pyramidal inversion of the σ^3^,λ^3^ P‐centre and the flipping of the helicene backbone have been evaluated computationally. The P‐inversion barrier for the transformation from (*M*,R_P_)‐**2**/(*P*,S_P_)‐**2** leading to (*M*,S_P_)‐**2**/(*P*,R_P_)‐**2** of ΔE^‡^ = 36.1 kcal·mol^−1^ (Table 1) is considerably lower than the epimerization barrier at the helical backbone (ΔE^‡^ = 44.8 kcal·mol^−1^, Table 1). Thus, the substantial (but not enormous) computed P‐inversion barrier is consistent with the high temperature required for this transformation in the experiments. Furthermore, the diastereomers (*M*,R_P_)‐**2**/(*P*,S_P_)‐**2** and (*M*,S_P_)‐**2**/(*P*,R_P_)‐**2** have similar relative energies (3.0 kcal·mol^−1^), favoring the former compared to the latter (again due to intramolecular π‐stacking). This slight energy difference (around the accuracy of the computation employed) is in a good qualitative agreement with the 6:4 ratio of the diastereomers observed in the experiments.

For both compounds **3** and **4** exhibiting more rigid perylene structures, two pairs of enantiomers with not too different relative energies were obtained in the geometry optimization runs, in line with the two types of chiralities. Remarkably, the relative energy of the two diastereomers of the oxidized **3** is larger (6.8 kcal·mol^−1^) compared to the reduced form **4**, similarly to the situation of compounds **1** and **2** (Table 1). Importantly, in these cases the backbone inversion involves the flipping of the peripheral phenyl moiety attached to the phosphepine ring (termed as “phosphepine backbone epimerization”), in contrast to the exchange between two naphthyl fragments as shown above for the couples **1** and **2**.

We targeted to find reasonable explanations why the diastereomer (R_P_,*P*)‐**3**/(S_P_,*M*)‐**3** could not be accessed in the experiments. Although the conversion of (R_P_,*M*)‐**3**/(S_P_,*P*)‐**3** to (R_P_,*P*)‐**3**/(S_P_,*M*)‐**3** has a moderate activation barrier of ΔE^‡^ = 31.8 kcal·mol^−1^, the process is thermodynamically disfavored by + 6.8 kcal·mol^−1^, prohibiting this conversion. As to the formation of (R_P_,*P*)‐**3**/(S_P_,*M*)‐**3** from the less stable isomer (*M*,S_P_)‐**1**/(*P*,R_P_)‐**1** in the Scholl conditions, two possibilities can be considered depending on the sequence of cyclization and epimerization. Among these, most likely, the first step is the cyclization of (*M*,S_P_)‐**1**/(*P*,R_P_)‐**1** to (R_P_,*P*)‐**3**/(S_P_,*M*)‐**3**, and, this isomer converts to the more stable form (R_P_,*M*)‐**3**/(S_P_,*P*)‐**3** through a barrier of 25.0 kcal·mol^−1^. The other alternative that (*M*,S_P_)‐**1**/(*P*,R_P_)‐**1** first epimerizes to (*M*,R_P_)‐**1**/(*P*,S_P_)‐**1** would require to pass through a much higher activation barrier of 37.7 kcal·mol^−1^.

The situation of compound **4** is more complicated, especially due to its rearomatization to PAH **5** (Figure 3). The calculated inversion barrier at the phosphepine backbone for phosphine (R_P_,*M*)‐**4** to (R_P_,*P*)‐**4** is ΔE^‡^ = 32.3 kcal·mol^−1^, similar to that obtained for the oxidized analogue **3** (Table 1, Figure 3, central part) Interestingly, this value is just slightly lower than the pyramidal inversion barrier at the P‐center of ΔE^‡^ = 34.6 kcal·mol^−1^(Figure 3), leading from (R_P_,*M*)‐**4** to (S_P_,*M*)‐**4′**. Note that surprisingly, another pathway (Figure 3) was also found for the same conversion, in which (R_P_,*M*)‐**4** first undergoes an epimerization to (R_P_,*P*)‐**4** via a backbone inversion (ΔE^‡^ = 32.3 kcal·mol^−1^ at TS_backbone_). Note that (R_P_,*P*)‐**4** and (S_P_,*M*)‐**4′** are in a rotamer relation, (S_P_,*M*)‐**4** can be obtained simply by the rotation of the phenyl group (with a negligible barrier of ΔE^‡^ = 6.8 kcal·mol^−1^). As (S_P_,*M*)‐**4** was not observed in experiments, we studied further decomposition pathways. Because of their low aromaticity, phosphepines are known to be thermally labile,^[^ ^22^ ^]^ and commonly decompose via a phosphanorcaradiene intermediate that expels a phosphinidene fragment, gaining aromatic stabilization. In our case the rigid aromatic side rings are expected to significantly contribute to the stability of the phosphepine and such transformation only occurs at high temperature as employed in the experiments. We computationally explored the possible formation of compound **5** and phenyl phosphinidene (PPh) from compound **4** (Figure 3, decomposition paths). In the first reaction step, the two carbon atoms neighboring the P center in (R_P_,*M*)‐**4** approach each other, leading to the (R_P_,*M*)‐**INT** intermediate through an energy barrier of ΔE^‡^ = 33.2 kcal·mol^−1^ (Figure 3, left). Starting from this intermediate, compound **5** and PPh may easily form via (R_P_,*M*)‐**TS2**, crossing over a negligible activation barrier of only ΔE^‡^ = 0.5 kcal·mol^−1^. A highly similar decomposition pathway was determined for the other diastereomer (S_P_,*M*)‐**4** as well (Figure 3, right side), and the activation barrier on this route is even smaller. Although the decomposition reaction is quite endothermic by 21.5 kcal·mol^−1^, the follow‐up chemistry of the resulting phosphinidene may further contribute to the thermodynamic sink of the reaction. Indeed, the electron‐deficient nature of phosphinidenes makes them highly reactive toward self‐association to form oligomers. The simplest oligomerization reaction is the dimerization to diphosphene (e.g., P_2_Ph_2_), which is calculated to be highly exothermic (ΔE = −85 kcal·mol^−1^), further promoting thermodynamically the decomposition of phosphepines. While, based on the energy barriers, the two epimerization pathways seem reachable at high temperature (T = 140 °C), the decomposition (rearomatization) may also happen parallelly as its activation barrier is clearly in the range of the epimerization processes. Therefore, the final product of the thermolysis is the PAH **5**.

Given the decomposition of **4**, the question may arise, why the rearomatization of **2** was not observed in the experiments. Therefore, we calculated the activation barrier for the extrusion of phosphinidene from the isomer (*M*,S_P_)‐**2**/(*P*,R_P_)‐**2**, which is enormously high (E^‡^ = 49.2 kcal·mol^−1^), explaining that this reaction is kinetically hampered. In contrast to the planar backbone of compound **4**, the helical curvature in phosphepine **2** most likely hinders the formation of a planar norcaradiene moiety.

**Figure 4:**
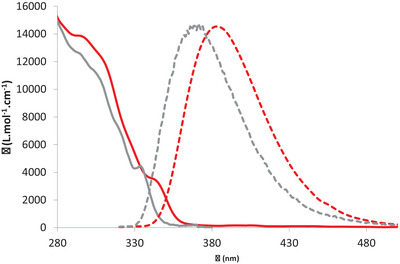
UV–vis absorption (solid line) and normalized emission (dotted) spectra of (*M*,R_P_)‐**1**/(*P*,S_P_)‐**1** (grey) and (*M*,S_P_)‐**1**/(*P*,R_P_)‐**1** (red) in DCM (c = 5.10^−6^ M).

The peculiar stereodynamic properties of phosphepine‐based polyaromatics allowed us to observe molecular diversity in the phosphepine‐containing helicenoid **1** but not in the perylenoid **3** due to competitive processes between the epimerization of the two stereogenic centers (P‐atom/helicenoid backbone) and the rearomatization leading to PAH **5**. The chiroptical properties of the new chiral derivatives will now be discussed in detail. The spectroscopic properties of the new diastereomers were investigated in diluted DCM solutions (c = 5·10^−6^ mol·L^−1^, Figure 4 and Table S3) at room temperature. To understand the nature of electronic excitations, TD‐DFT (time‐dependent density functional theory, using the B3LYP functional combined with the cc‐pVDZ basis set) and ADC(2) methods (second‐order algebraic diagrammatic construction combined with the cc‐pVTZ basis set) calculations were performed.

**Figure 5:**
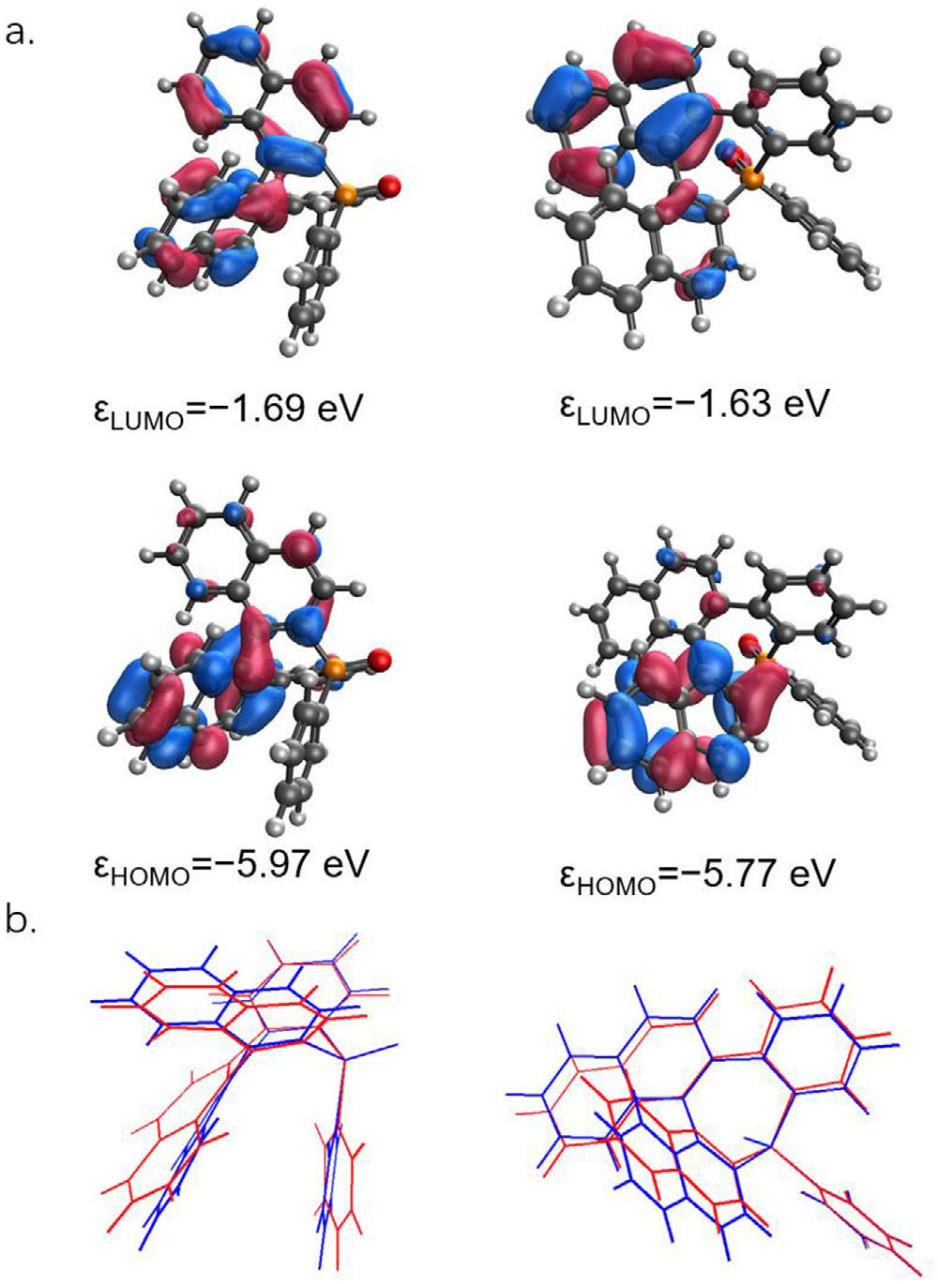
(a) Electronic distribution of LUMO (top) and HOMO (bottom) of (*M*,R_P_)‐**1** (left), (*M*,S_P_)‐**1** (right), at the B3LYP/cc‐pVDZ level plotted with a contour value of 0.1 (b) overlapped geometries of the ground state (blue) and excited state (red) of (*M*,R_P_)‐**1** (left), (*M*,S_P_)‐**1** (right).

Phosphine oxide (*M*,S_P_)‐**1**/(*P*,R_P_)‐**1** displays UV/vis spectra consisting of a strong absorption band at 296 nm (*ε* = 1,4·10^4^ M^−1^ cm^−1^) with a shoulder of lower intensity (*ε* ∼ 3,8·10^3^ M^−1^ cm^−1^) at 344 nm (Figure 4). TD‐DFT calculations give computed absorption wavelengths for the first excitation at 354 nm agreeing nicely with the value experimentally obtained for the shoulder. The spectrum of (*M*,S_P_)‐**1**/(*P*,R_P_)‐**1** is slightly redshifted compared to its diastereoisomer (*M*,R_P_)‐**1**/(*P*,S_P_)‐**1^[^** ^17^ ^]^ (see Figure 4). Based on our DFT calculations, this first excitation can be attributed to practically pure HOMO–LUMO transition of π–π* character for each of the diastereomers. The distributions of the HOMO and LUMO are different on the two helicenoid fragments leading to considerable charge transfer (Figure 5). Moreover, the HOMO–LUMO energy gap is slightly smaller for (*M*,S_P_)‐**1**/(*P*,R_P_)‐**1** (4.1 eV) than for (*M*,R_P_)‐**1**/(*P*,S_P_)‐**1** (4.3 eV), being consistent with the observed red‐shift of the UV–vis spectrum.

The emission of the racemate (*M*,S_P_)‐**1**/(*P*,R_P_)‐**1** was, then, examined in DCM at a concentration of 10^−5^ M (Figure 4). At room temperature, (*M*,S_P_)‐**1**/(*P*,R_P_)‐**1** displays blue emission at 384 nm with a quantum yield of 0.09, and a luminescence lifetime of 2.3 ns, typical for fluorescent compounds. The emission is red‐shifted compared to the diastereoisomer (*M*,R_P_)‐**1**/(*P*,S_P_)‐**1** (Δλ = 13 nm). The ADC(2) calculation performed on the relaxed geometries obtained by the TD‐DFT optimizations also outline substantial Stokes‐shifts (1745 cm^−1^ for (*M*,R_P_)‐**1** and 2510 cm^−1^ for (*M*,S_P_)‐**1**), being in the ranges of the experimental results (2856 cm^−1^ for (*M*,R_P_)‐**1** and 3130 cm^−1^ for (*M*,S_P_)‐**1**). These remarkable Stokes‐shifts can be explained by the twisting of the helicene backbones in the excited states (in line with the asymmetrical spatial distribution of the π systems on the helixes, Figure 5). Low temperature emission allowed to localize the triplet level (Figure S10), which again appeared slightly red‐shifted compared to the parent diastereoisomer, thus resulting in similar singlet–triplet energy gap (Table S3; the calculated singlet–triplet gaps for both (*M*,R_P_)‐**1**/(*P*,S_P_)‐**1** and (*M*,S_P_)‐**1**/(*P*,R_P_)‐**1** are 57.0 kcal·mol^−1^).

**Figure 6:**
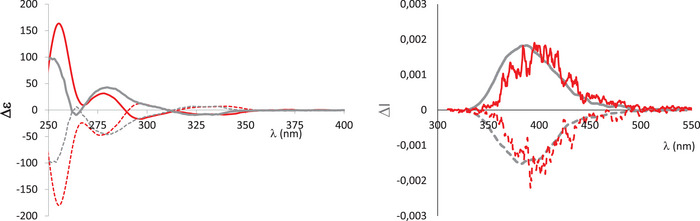
ECD and CPL spectra of (*P*,S_P_)‐**1** (grey), (*M*,R_P_)‐**1** (grey dotted), (*P*,R_P_)‐**1** (red), and (*M*,S_P_)‐**1** (red dotted) in diluted DCM at 10^−5^ M.

Electronic circular dichroism (ECD) spectra were then recorded in diluted DCM solutions. The ECD spectra of (*M*,S_P_)‐**1** and (*P*,R_P_)‐**1** display the expected mirror‐image relationship (Figure 6). The spectrum of (*P*,R_P_)‐**1** consists of positive ECD bands (Δε = +160 at 256 nm and +30 at 280 nm) followed by negative bands (Δε = −17 M^−1^ cm^−1^ at 296 nm and −9 M^−1^ cm^−1^ at 339 nm). For these red‐shifted transitions, the g_abs_ values are ∼3.10^−3^. Interestingly, the global shape is rather different compared to the other diastereoisomers (*M*,R_P_)‐**1** and (*P*,S_P_)‐**1**,^[^ ^17^ ^]^ illustrating that both the conformation of the helical backbone and the P‐atom strongly influences the chiroptical properties. Finally, (*M*,S_P_)‐**1** (resp. (*P*,R_P_)‐**1**) also display circularly polarized luminescence (CPL) with g_lum_ ∼ 2.10^−3^ at 400 nm, in good correlation with the g_abs_ value (Figure 6). The sign of the CPL seems to be governed by the configuration of the helical backbone (the two positive CPL emitters display a *M*‐helicenoid). The g_lum_ values of (*M*,S_P_)‐**1**/(*P*,R_P_)‐**1** being rather classical for organic CPL emitters,^[^ ^23^ ^]^ have similar dissymmetry factors but are again slightly red‐shifted compared to (*M*,R_P_)‐**1** and (*P*,S_P_)‐**1**.^[^ ^17^ ^]^ The TD‐DFT calculations are also fully consistent with the experimental ECD spectra. (*P*,R_P_)‐**1** shows a negative ECD band at 268 nm (rotatory strength = −54.6) while, (*P*,S_P_)‐**1** exhibits a negative ECD band at 262 nm (rotatory strength = −188.7).

## 3 Conclusion

In conclusion, we have presented that taking advantage of stereodynamism in phosphahelicenoids via the inversion of a trivalent P‐center allowed us to generate molecular diversity in chiral phosphepine helicenoids **1**. However, due to a complex interplay between P‐epimerization, helicenoid inversion and aromatization upon loss of the P center, such strategy is not feasible for the more fused phosphepine perylenoid **3** but leads to a naphtho perylene isomer instead. In addition, the epimerization and rearomatization processes are explained by in‐depth computational investigations. The newly formed chiral phosphepines display helicene‐like chiroptical properties, including circularly polarized luminescence. TD‐DFT and ADC(2) calculations have been employed to confirm that the source of the dissymmetry factors lies in the asymmetric spatial distribution of the molecular orbitals involved in the transitions. This study outlines that seven‐membered P‐rings are fascinating building blocks to prepare contorted and chiral π‐systems and eventually can be used as molecular platform to access larger chiral nanographenes.

## 4 Experimental Section

All experiments were performed under an atmosphere of dry argon using standard Schlenk techniques. Commercially available reagents were used as received without further purification. Solvents were freshly purified using MBRAUN SPS‐800 drying columns filled with Al_2_O_3_. Separations were performed on air by gravity column chromatography on basic alumina (Aldrich, Type 5016A, 150 mesh, 58 Å) or silica gel (Merck Geduran 60, 0.063–0.200 mm). Enantiomeric excesses were determined by high performance liquid chromatography (HPLC) analysis on Alliance e2695 Waters HPLC with a UV/vis detector 2489 Waters at 254 nm. HPLC method: OD‐3 column (0.46 cm x 25 cm) as stationary chiral phase and with hexane (70%) and isopropanol (30%) at 1.0 mL/min as mobile phase at 25 °C and λ = 254 nm. ^1^H, ^13^C, and ^31^P NMR spectra were recorded on a Bruker AV III 400 MHz NMR spectrometer equipped with BBFO probehead. High‐resolution mass spectra were obtained on Bruker Maxis 4G instrument at Scanmat (UAR 2025). UV–vis spectra were recorded at room temperature on a JASCO V‐630 spectrophotometer. The UV–vis emission and excitation spectra measurements were recorded on an FL 920 Edinburgh Instrument and corrected for the response of the photomultiplier. Quantum yields were calculated relative to quinine sulfate (Φ = 0.54 in H_2_SO_4_ 0.1N, ϕ_ref_ = 0.55). Lifetimes measurements were conducted with 295 nm pulsed LED excitation (EDEPLED‐300) plugged to a TCSPC pulsed source interface using an Edinburgh FS920 Steady State Fluorimeter combined with a FL920 Fluorescence Lifetime Spectrometer. The CD spectra measurements were recorded on Spectropolarimeter of circular dichroism J‐815 (Jasco France), and the optical rotations measurements were carried out on Perkin Elmer 341 instrument with sodium light at 589 nm. (*M*,R_P_)‐**1**/(*P*,S_P_)‐**1** and (R_P_,*M*)‐**3**/(S_P_,*P*)‐**3** were synthesized according to the previous procedure.^[^ ^17^, ^24^ ^]^

(*M*,S_P_)‐**1**/(*P*,R_P_)‐**1**, (*M*,R_P_)‐**1**/(*P*,S_P_)‐**1** (180 mg, 0.398 mmol, 1 equiv) is dissolved in 18 mL of dry toluene. HSiCl_3_ (0.41 mL, 3.98 mmol, 10 equiv) is added dropwise and then heated to reflux 1 h 30 min. The solvent is evaporated under vacuum, and the resulting compound is dissolved in 18 mL of dry DMF, then heated to 160 °C overnight. The solvent is evaporated and the crude mixture is dissolved in 25 mL of DCM with 1 L of H_2_O_2_/H_2_O (1:1). After extraction, the crude mixture was purified by silica gel chromatography using DCM/AcOEt (8/2) to afford (*M*,S_P_)‐**1**/(*P*,R_P_)‐**1** (65 mg, η = 36%) as the first eluted compound and the starting compound (*M*,R_P_)‐**1**/(*P*,S_P_)‐**1** (98 mg, η = 54%) as second eluted compound. ^1^H NMR (400 MHz, CD_2_Cl_2_) δ 8.12–8.02 (m, 3H), 7.98 (dd, *J* = 8.3, 1.3 Hz, 1H), 7.90–7.82 (m, 3H,), 7.81–7.68 (m, 4H), 7.56 (tt, *J* = 8.6, 1.9 Hz, 1H), 7.53–7.43 (m, 3H), 7.36 (ddd, *J* = 12.9, 7.8, 1.3 Hz, 1H), 7.28–7.13 (m, 4H), 6.88 (dd, *J* = 8.6, 1.1 Hz, 1H). ^13^C NMR (101 MHz, CD_2_Cl_2_) δ 143.6 (d, *J* = 6.4 Hz, C_q_), 139.3 (d, *J* = 102.2 Hz, C_q_), 139.0 (d, *J* = 6.7 Hz, C_q_), 137.2 (d, *J* = 1.3 Hz, C_q_), 136.9 (d, *J* = 102.3 Hz, C_q_), 135.5 (d, *J* = 8.0 Hz, C_ortho_), 134.6 (d, *J* = 2.3 Hz, C_q_), 134.0 (d, *J* = 11.4 Hz, C_q_), 133.6 (C_q_), 133.3 (d, *J* = 2.9 Hz, C_q_), 132.9 (d, *J* = 3.3 Hz, C_para_), 132.9 (C_q_), 132.1 (d, *J* = 2.4 Hz, CH), 131.2 (d, *J* = 9.4 Hz, CH), 129.5 (d, *J* = 11.5 Hz, C_2_), 129.3 (C_9_), 129.1 (C_8_), 128.7 (C_19_), 128.6 (d, *J* = 11.5 Hz, C_meta_), 128.6 (C_11_), 128.4 (C_14,22_), 128.0 (d, *J* = 12.3 Hz, CH), 127.9 (CH), 127.0 (d, *J* = 12.3 Hz, CH), 127.0 (CH), 126.8 (CH), 126.5 (CH), 125.9 (d, *J* = 104.5 Hz, C_ipso_), and 125.1 (d, *J* = 12.0 Hz, CH). ^31^P NMR (162 MHz, CD_2_Cl_2_) δ +20.4. HRMS (ESI, CH_3_OH/DCM_:_ 9/1) [M+H]^+^ (C_32_H_22_OP): *m/z* Theoretical: 453.1403, m/z Found: 453.1397.

The computational results were obtained with the Gaussian 16^[^ ^25^ ^]^ and MRCC^[^ ^26^ ^]^ suites of programs. All structures were optimized using the B3LYP‐D3 method combined with the cc‐pVDZ basis set, and then single‐point calculations were also employed at the LNO‐CCSD(T)/cc‐pVTZ level. In the case of all the optimized structures, vibrational analysis was carried out to check whether the stationary point located on the potential energy hypersurface is a minimum (no imaginary frequencies were obtained) or a transition state (one imaginary frequency). Intrinsic Reaction Coordinate (IRC) calculations were performed to locate the minima connected by the transition state. The stability of the wavefunction was tested for the stationary point in the extrusion of phosphinidene. In case of instability, unrestricted formalism was used. To verify the TD‐DFT results, the ADC(2) method combined with the cc‐pVTZ basis set was also used. For the TD‐DFT and ADC(2) calculations, the first 20 excitations were considered. The molecular orbitals were visualized with IQmol.^[^ ^27^ ^]^ The ECD spectra were plotted with the GaussSum program.^[^ ^28^ ^]^

## Conflict of Interests

The authors declare no conflict of interest.

## Supporting information



Supporting Information

Supporting Information

## Data Availability

The data that support the findings of this study are available from the corresponding author upon reasonable request.
