# Supplementary material for: Stereodynamism in Chiral Polyaromatic Phosphepines
Source: Chemistry. 2025 May 19;31(34):e202500343. doi: 10.1002/chem.202500343 (PMC12172589; doi:10.1002/chem.202500343)
Supplement: Supplementary file 1 — Supporting Information [file CHEM-31-e202500343-s001.docx]

**Stereodynamism in Chiral Polyaromatic Phosphepines**

Mengling Lyu,^a^ Thomas Delouche,^b^ Réka Mokrai, ^a,b^ Thomas Vives,^b^ Thierry Roisnel,^b^ Muriel Hissler,^b^ Zoltán Benkő,* ^a,c^ Pierre-Antoine Bouit*^b^

1. Department of Inorganic and Analytical Chemistry, Budapest University of Technology and Economics, Műegyetem rkp. 3, H-1111 Budapest.
2. Univ Rennes, CNRS, ISCR - UMR 6226, F-35000 Rennes.
3. HUN-REN-BME Computation-Driven Chemistry Research Group, Műegyetem rkp. 3, H-1111 Budapest.

**Table of Content**

**NMR spectra page S2**

**Crystallographic data and structure refinement parameters page S5**

**Photophysical properties page S9**

**Chiral chromatography data** **page S10**

**Theoretical calculations page S12**

**NMR spectra**

Figure S1: ^1^H NMR (400 MHz, CD_2_Cl_2_, 293K) spectra of compound (*M* ,S_P_)-**1**/ (*P* , R_P_)-**1.**

Figure S2: ^31^P{^1^H} NMR (162 MHz, CD_2_Cl_2_, 293K) spectra of compound (*M*,S_P_)-**1**/ (*P* ,R_P_)-**1.**

Figure S3: ^13^C NMR (101 MHz, CD_2_Cl_2_, 293K) spectra of compound (*M* ,S_P_)-**1**/ (*P*,R_P_)-**1.**

Figure S4: *in situ* ^31^P NMR following of the deprotection-oxidation sequence on (*M,* S_P_)-**1**/(*P*,R_P_)-**1**

Figure S5: *in situ* ^31^P NMR following of the epimerization of (*M,*S_P_)-**2**/ (*P* ,R_P_)-**2** in DMF at 140°C for one night

Figure S6: *in situ* ^31^P NMR following of the deprotection-epimerization sequence on (R_P_,*M*)-**3** / (S_P_,*P*)-**3**

**Crystal structure determination**:

Single crystals suitable for X-Ray crystal analysis were obtained by slow diffusion of vapors of pentane into a dichloromethane solution of the derivatives at room temperature. Single crystal data collection were performed at 150 K with an APEXII diffractometer with Mo-*K*α radiation (*λ* = 0.71073 Å). The structure was solved by dual-space algorithm using the *SHELXT* program^[[1]](#footnote-1)^, and then refined with full-matrix least-squares methods based on *F*^2^(*SHELXL*)^[[2]](#footnote-2)^. All non-hydrogen atoms were refined with anisotropic atomic displacement parameters. H atoms were finally included in their calculated positions and treated as riding on their parent atom with constrained thermal parameters.

Table S1 : crystallographic data

| **Compound** | (*M,* S_P_)-**1**/ ( *P*,R_P_)-**1** | (*M*,S_P_,)-**1** | (*P*,R_P_,)-**1** |  |
| --- | --- | --- | --- | --- |
| CCDC | 2331801 | 2331811 | 2331808 |  |
| Formula | C_32_ H_21_ O P | C_32_ H_21_ O P | C_32_ H_21_ O P |  |
| MW | 452.46 | 452.46 | 452.46 |  |
| a (Å) | 8.3401(5) | 26.645(6) | 12.8870(13) |  |
| b (Å) | 11.3671(6) | 8.0490(17) | 16.1932(18) |  |
| c (Å) | 13.9059(8) | 11.873(3) | 26.002(3) |  |
| α (°) | 106.620(2) | 90 | 90 |  |
| β (°) | 98.993(2) | 108.670(8) | 90 |  |
| γ (°) | 109.860(2) | 90 | 90 |  |
| V (Å^3^) | 1140.15(11) | 2412.4(10) | 5426.2(10) |  |
| Z | 2 | 4 | 8 |  |
| *D*c (g.cm^-3^) | 1.318 | 1.246 | 1.108 |  |
| Crystal system | triclinic | monoclinic | orthorhombic |  |
| Space group | P -1 | C 2 | P 2_1_ 2_1_ 2_1_ |  |
| T (K) | 150(2) | 150(2) | 150(2) |  |
| WavelengthMo-Kα (Å) | 0.71073 | 0.71073 | 0.71073 |  |
| μ (mm^-1^) | 0.144 | 0.137 | 0.121 |  |
| *F* (000) | 472 | 944 | 1888 |  |
| θ limit (°) | 2.648 to 27.648 | 2.656 to 27.537 | 2.635 to 27.527 |  |
|  | -6 ≤ h ≤ 10 | -33 ≤ h ≤ 34 | -16 ≤ h ≤ 16 |  |
| Index ranges *hkl* | -14 ≤ k ≤ 13 | -9 ≤ k ≤ 10 | -21 ≤ k ≤ 19 |  |
|  | -18 ≤ l ≤ 17 | -15 ≤ l ≤ 14 | -29 ≤ l ≤ 33 |  |
| Reflections collected | 12007 | 8166 | 24682 |  |
| Independant reflections | 5234 | 4865 | 12231 |  |
| Reflections [*l*>2σ(*l*)] | 3688 | 4269 | 6809 |  |
| Data / restraints / parameters | 5234 / 0 / 307 | 4865 / 1 / 307 | 12231 / 0 / 541 |  |
| Goodness-of-fit on *F*^2^ | 1.014 | 1.040 | 0.960 |  |
| Final *R* indices [l>2σ(l)] | R1 = 0.0484 | R1 = 0.0478 | R1 = 0.0709 |  |
|  | wR2 = 0.1176 | wR2 = 0.1063 | wR2 = 0.1548 |  |
| R indices (all data) | R1= 0.0816 | R1= 0.0569 | R1= 0.1390 |  |
|  | wR2= 0.1408 | wR2= 0.1113 | wR2= 0.1884 |  |
| Largest diff peak / hole (e Å^-3^) | 0.332 and -0.563 | 0.296 and -0.355 | 0.288 and -0.301 |  |


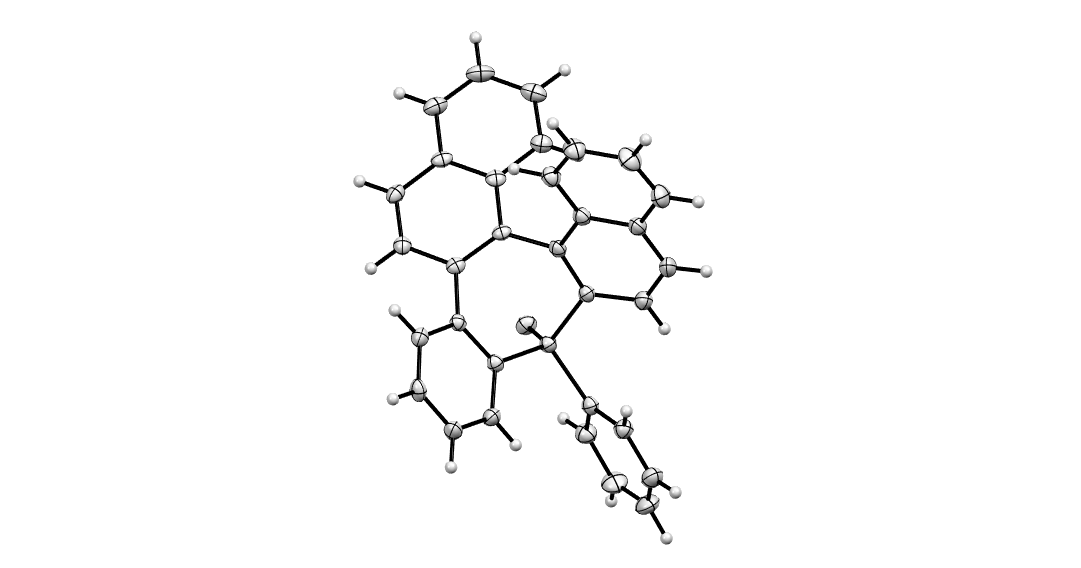


Figure S7: ORTEP representation of (*M,* S_P_)-**1**/ (*P,* R_P_)-**1** with 50% probability ellipsoids


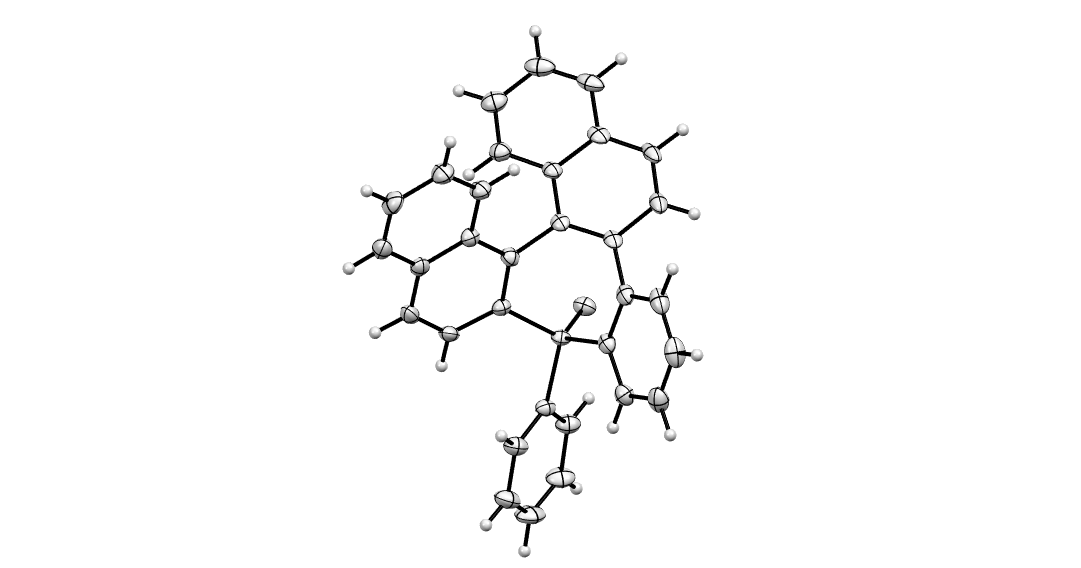


Figure S8: ORTEP representation of (*M,* S_P_)-**1** with 50% probability ellipsoids


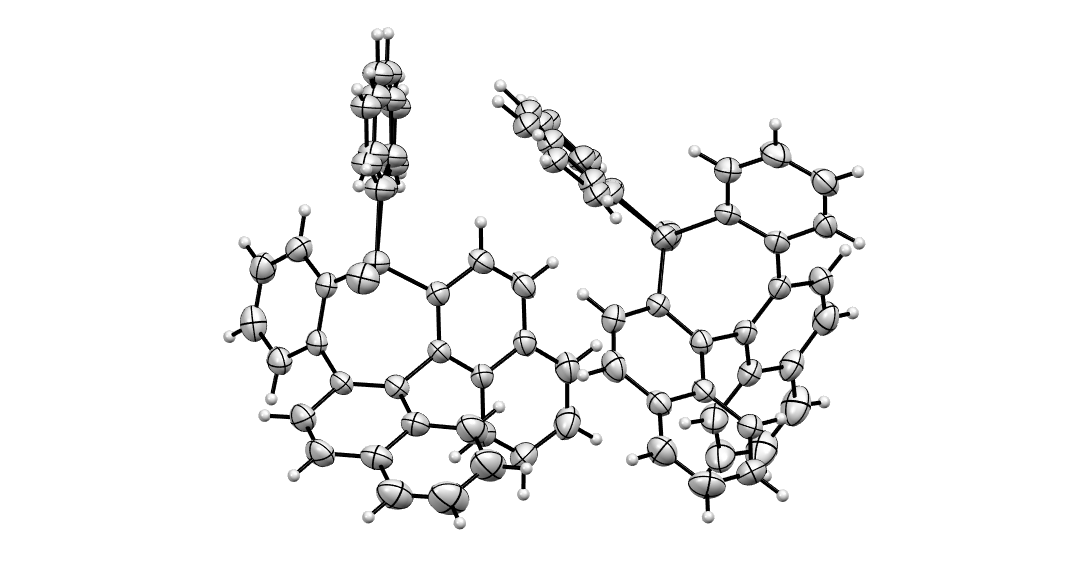


Figure S9: ORTEP representation of (*P,* R_P_)-**1**-**1** with 50% probability ellipsoids

Table S2: Experimentally determined and DFT optimized geometrical parameters of (*M*,S_P_)-**1** (distances in Å angles in degrees)

|  | experimental | DFT |
| --- | --- | --- |
| P-O | 1.480(3) | 1.519 |
| P-C1 | 1.803(3) | 1.838 |
| P-C11 | 1.803(3) | 1.852 |
| C11-C16 | 1.414(5) | 1.419 |
| C16-C17 | 1.473(5) | 1.490 |
| C17-C26 | 1.387(5) | 1.395 |
| C26-C27 | 1.494(4) | 1.496 |
| C27-C36 | 1.390(4) | 1.396 |
| C36-P1 | 1.810(3) | 1.847 |
|  |  |  |
| C11-P-C36 | 99.65(17) | 99.024 |
| O-P-C11 | 114.33(15) | 112.56 |
| O-P-C36 | 109.45(15) | 112.68 |

**Photophysical data**

Table S3: photophysical data

|  | λ_abs_ ^a^ (nm) | ε  ^a^  (L.mol^-1^.cm^-1^) | λ_em_ ^a^ (nm) | Φ ^a,b^ | τ (ns) | ΔE_ST_ ^c^  (eV) |
| --- | --- | --- | --- | --- | --- | --- |
| (*M*,R_P_)-**1** / (*P,* S_P_)-**1**^[[3]](#footnote-3)^ | 333 | 3,7 | 370 | 0,18 | 3,3 | 1,16 |
| (*M*,S_P_)-**1** / (*P*,R_P_)-**1** | 344 | 3,4 | 384 | 0,09 | 2,3 | 1,11 |

[a] In DCM (10^-5^M) [b] Luminescence quantum yields measured relative to quinine sulfate (H_2_SO_4_, 0.1 M), Ф= 0,55 [c] singlet-triplet energy gap, extracted from the edge of fluorescence/phosphorescence curves in MeTHF at 77K (Fig. S10).

Fig S10: Emission of (*M*,S_P_)-**1** / (*P*,R_P_)-**1** in 2-methyltetrahydrofuran at room temperature (orange) and 77K (blue)

**Enantiomeric excess determination**

Enantiomeric excesses were determined by HPLC Analysis (High Performance Liquid chromatography) on Alliance e2695 Waters® HPLC with a UV/visible detector 2489 Waters® at 254nm.

HPLC method: OD-3 column (0.46 cm x 25 cm) as stationary chiral phase and with hexane (70%) and isopropanol (30%) at 1.0 mL/min as mobile phase at 25°C and λ = 254 nm

(*M*,S_P_)-**1** / (*P*,R_P_)-**1**

|  | Name | Retention Time | Area | % Area | Height |
| --- | --- | --- | --- | --- | --- |
| 1 |  | 6.216 | 16885604 | 50.08 | 953011 |
| 2 |  | 10.726 | 16830452 | 49.92 | 372266 |

(*M*,S_P_)-**1**

|  | Name | Retention Time | Area | % Area | Height |
| --- | --- | --- | --- | --- | --- |
| 1 |  | 10.596 | 18509598 | 100.00 | 412571 |

(*P*,R_P_)-**1**

|  | Name | Retention Time | Area | % Area | Height |
| --- | --- | --- | --- | --- | --- |
| 1 |  | 6.126 | 42334658 | 100.00 | 2252665 |

**Theoretical calculations**

(*M*,R_P_,*P*)-**1** (*P*,R_P_,*M*)-**1** Transition state


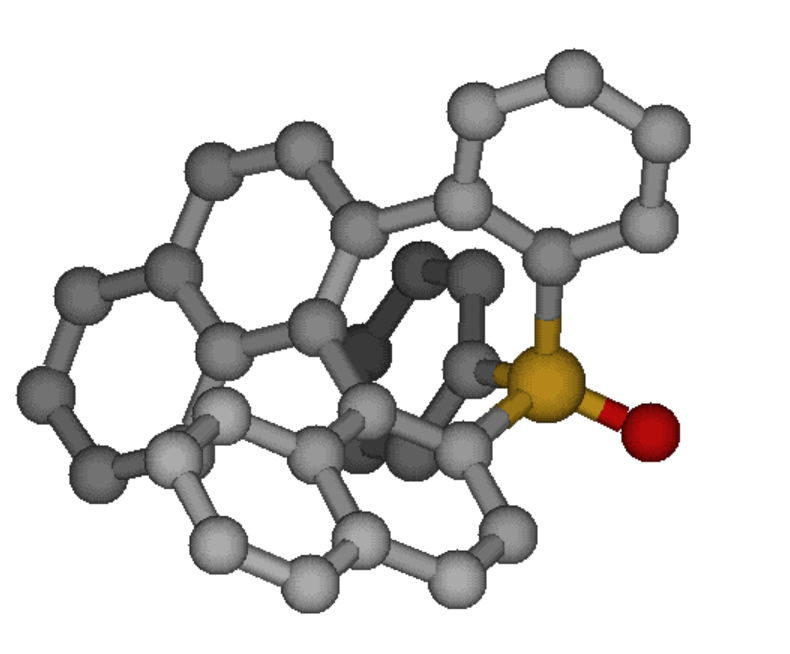

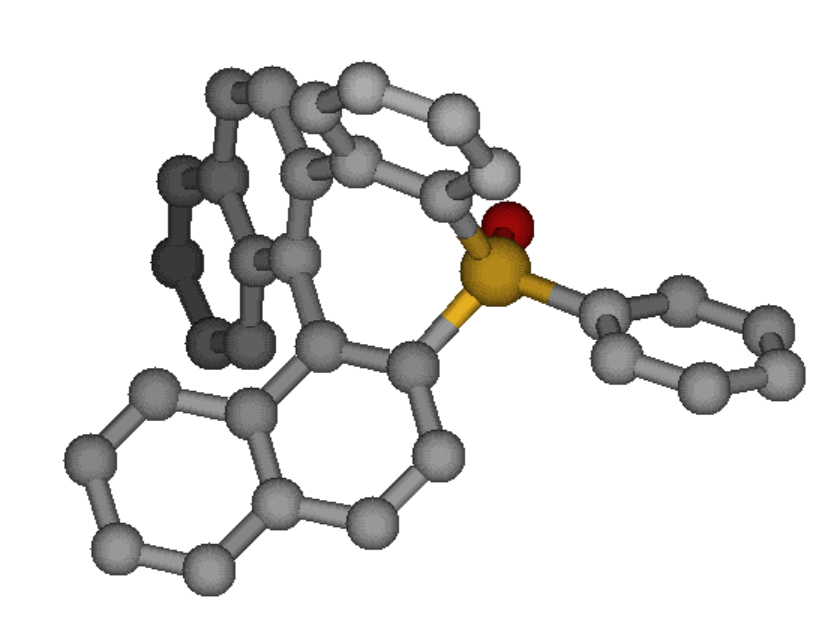

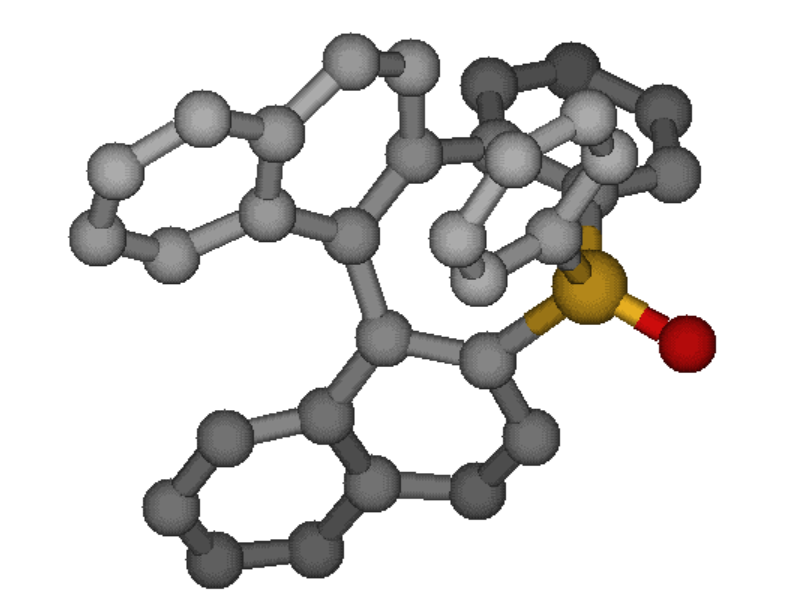


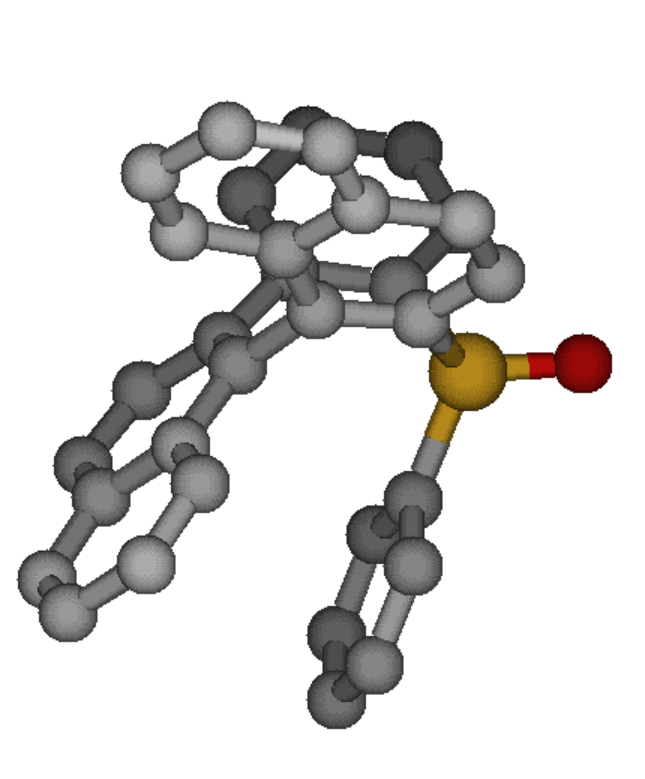

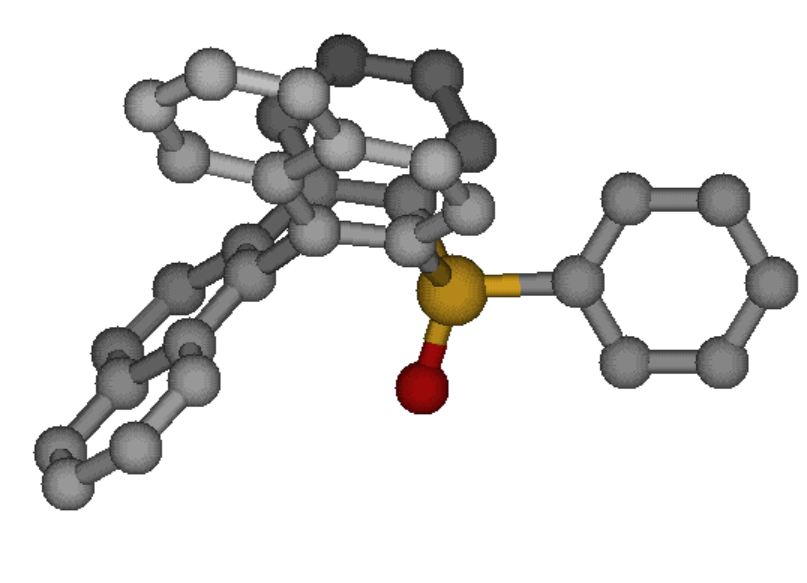

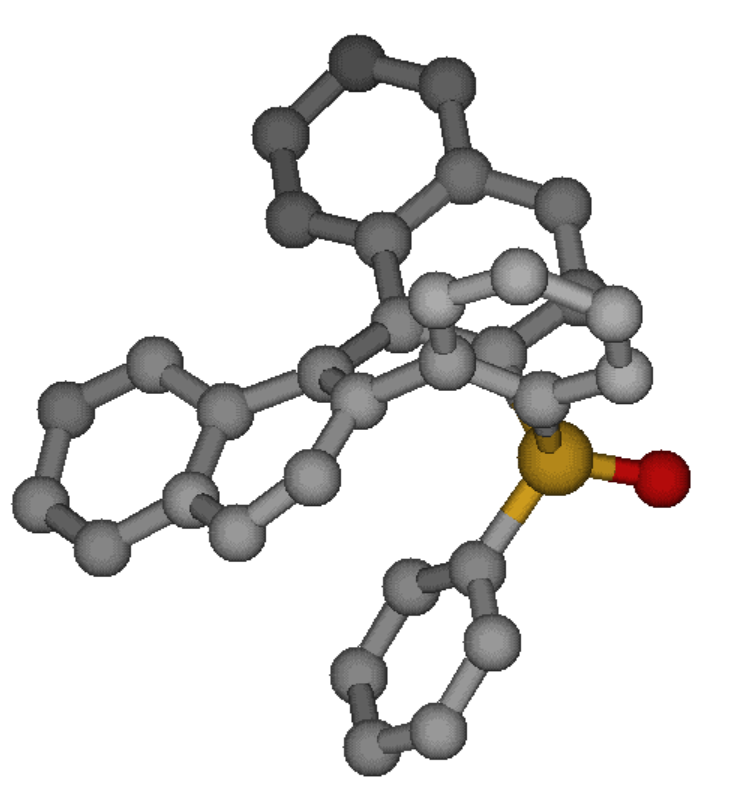


Figure S11: Top (up) and side (down) view of (*M*,R_P_,*P*)-**1** and (*P*,R_P_,*M*)-**1** and the transition state for backbone inversion, optimized at the B3LYP-D3/cc-pVDZ level (Hydrogen atoms were omitted for clarity.)

(*M*,R_P_,*M*)-**1**


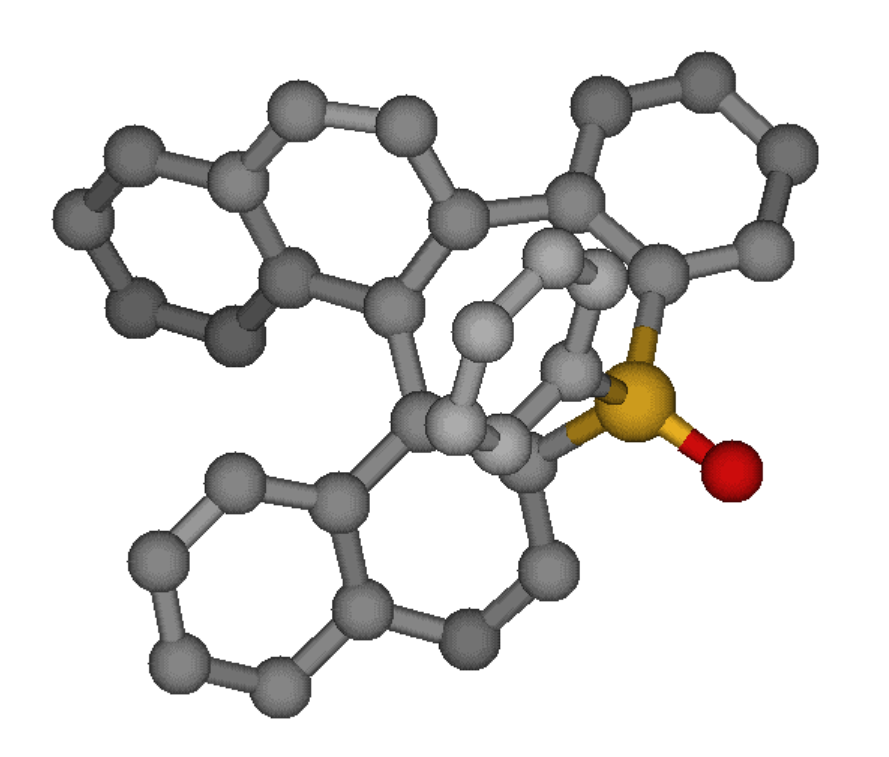


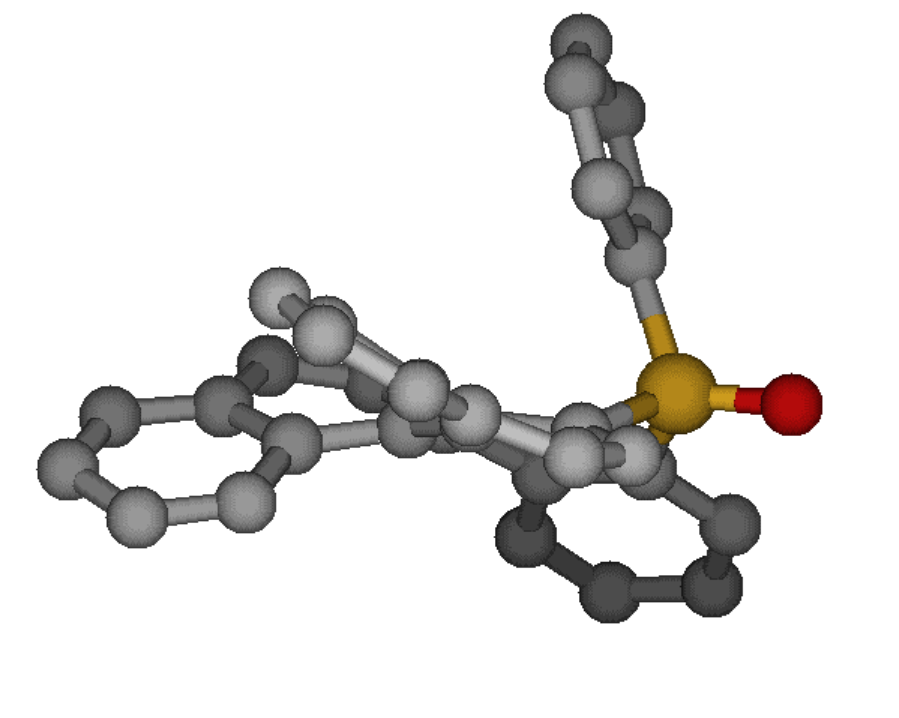


Figure S12: Top (up) and side (down) view of (*M*,R_P_,*M*)-**1**, optimized at the B3LYP-D3/cc-pVDZ level (Hydrogen atoms were omitted for clarity.)

(M,R_P_,*P*)-**2** (*M*,S_P_,*P*)-**2** Transition state


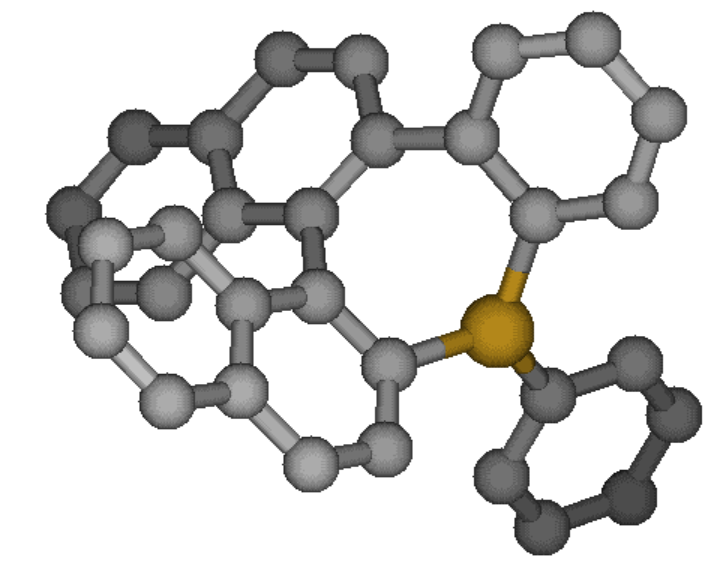

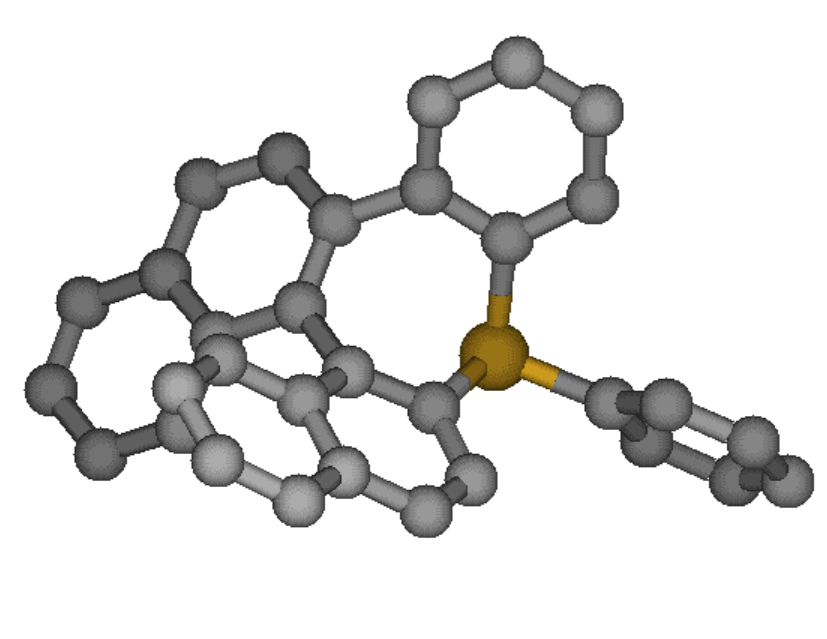

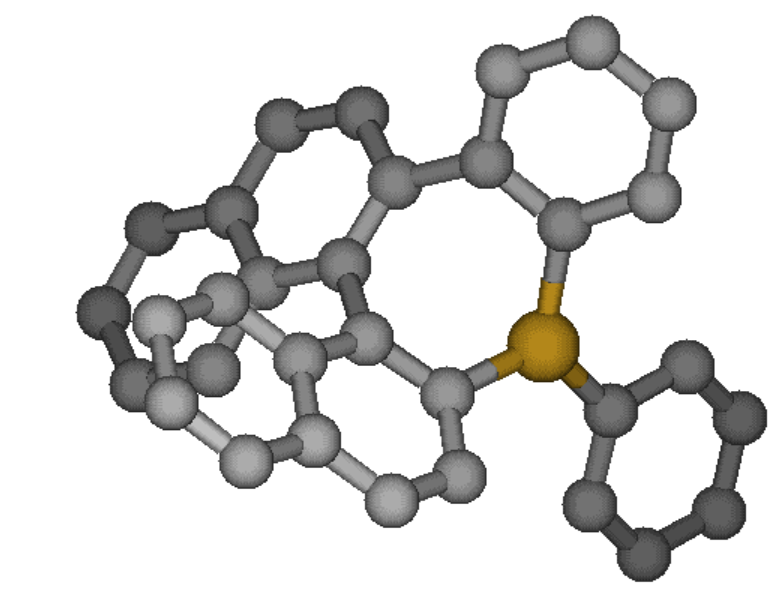


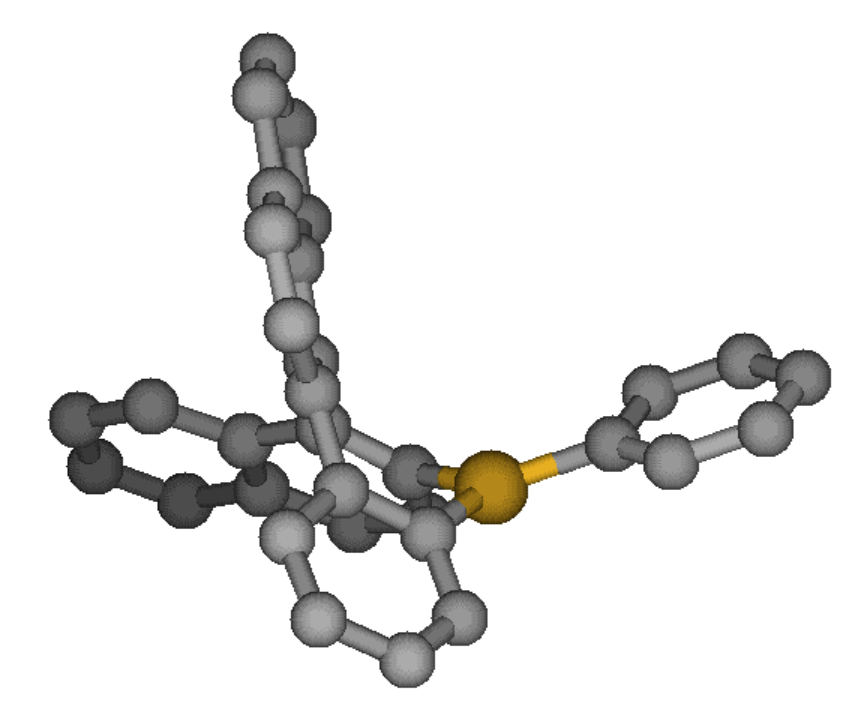

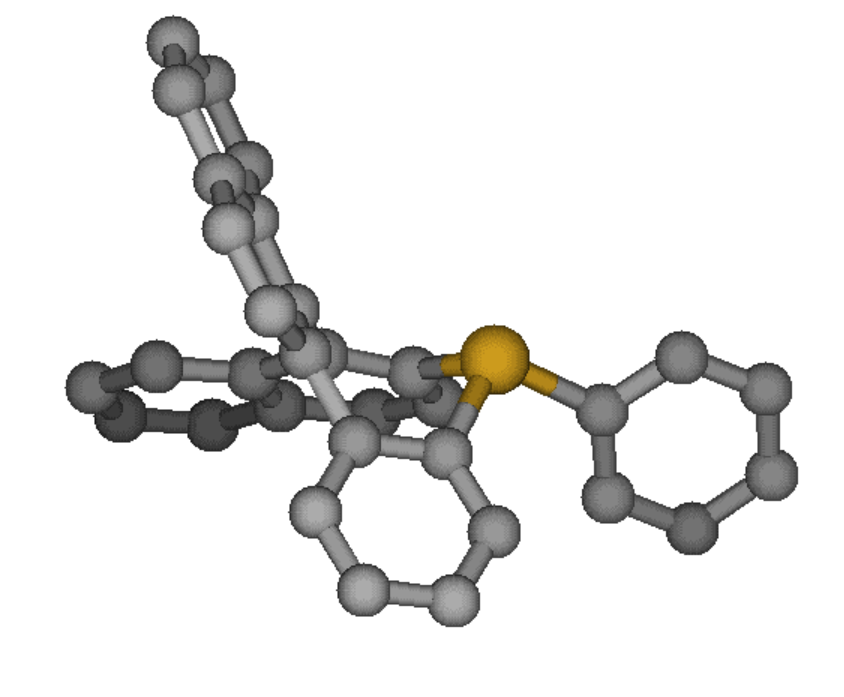

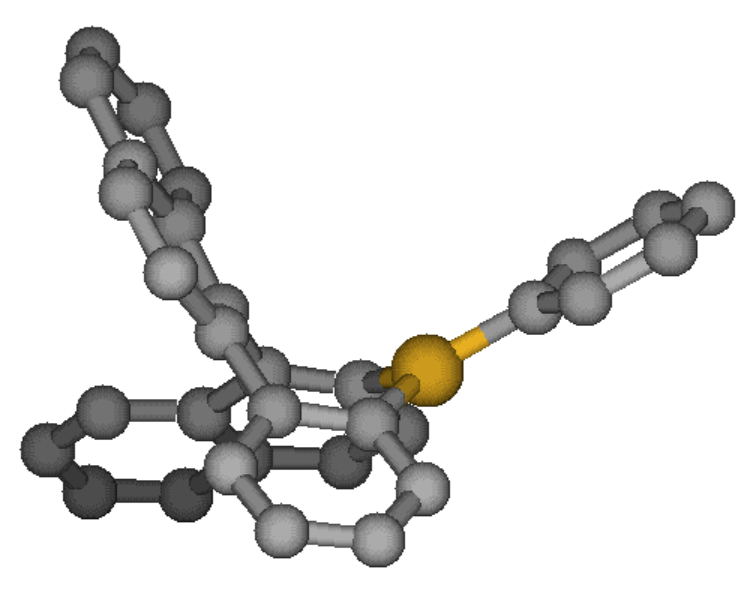


Figure S13: Top (up) and side (down) view of (M,R_P_,*P*)-**2** and (*M*,S_P_,*P*)-**2** and the transition state for inversion at the P-center, optimized at the B3LYP-D3/cc-pVDZ level (Hydrogen atoms were omitted for clarity.)

(*M*, R_P_, *M*)-**2**  (*P*, R_P_, *M*)-**2** Transition state


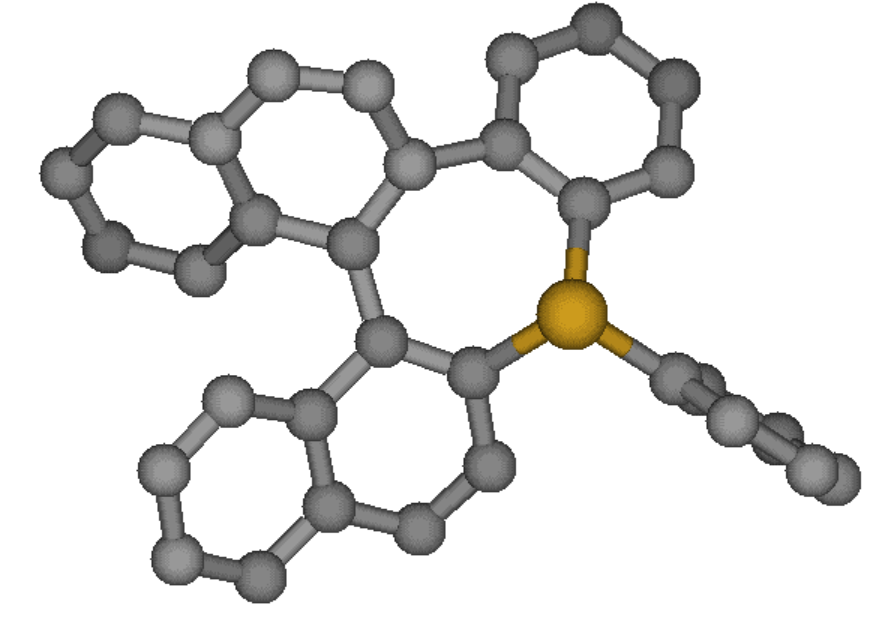

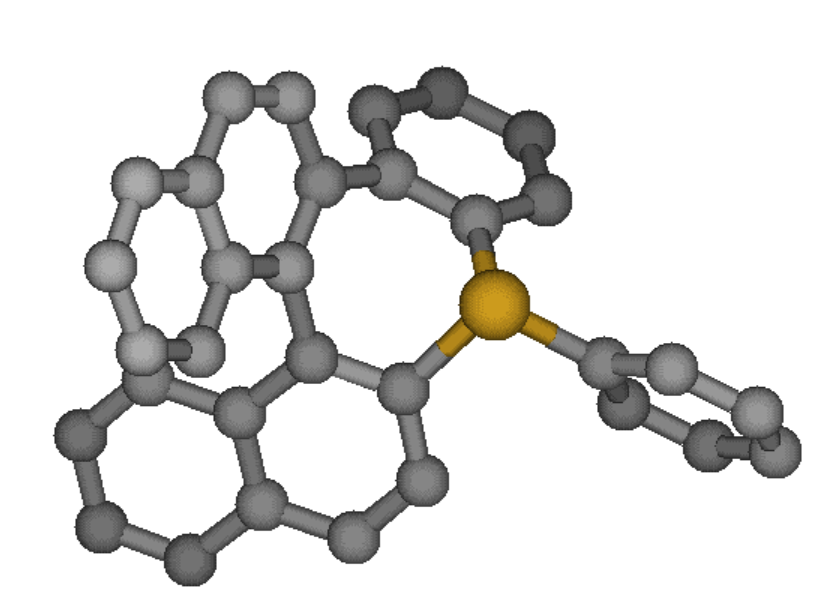

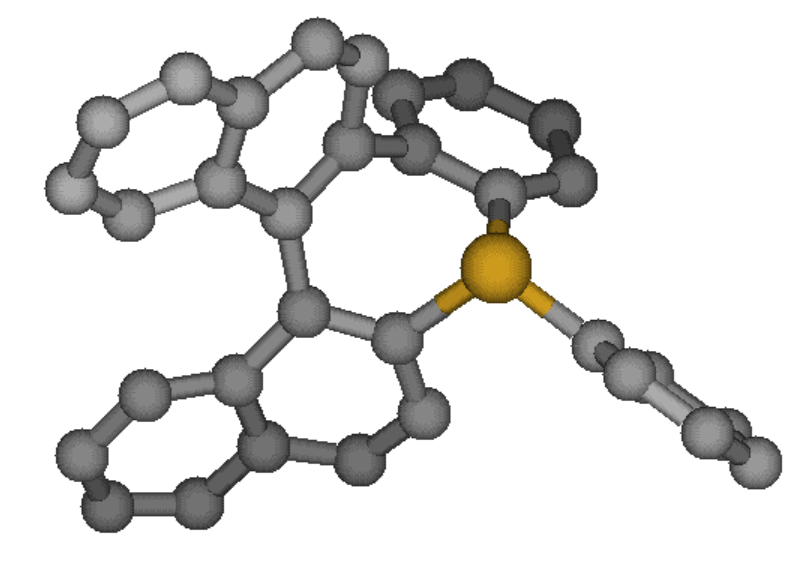


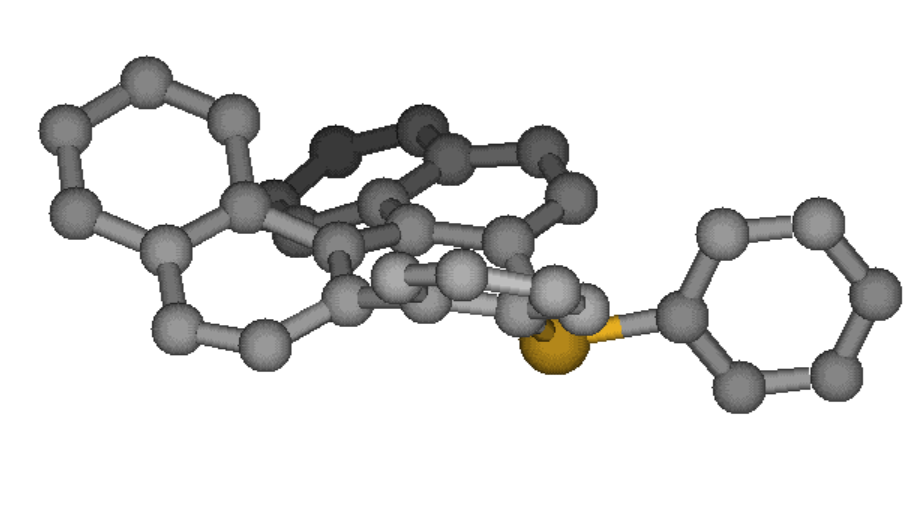

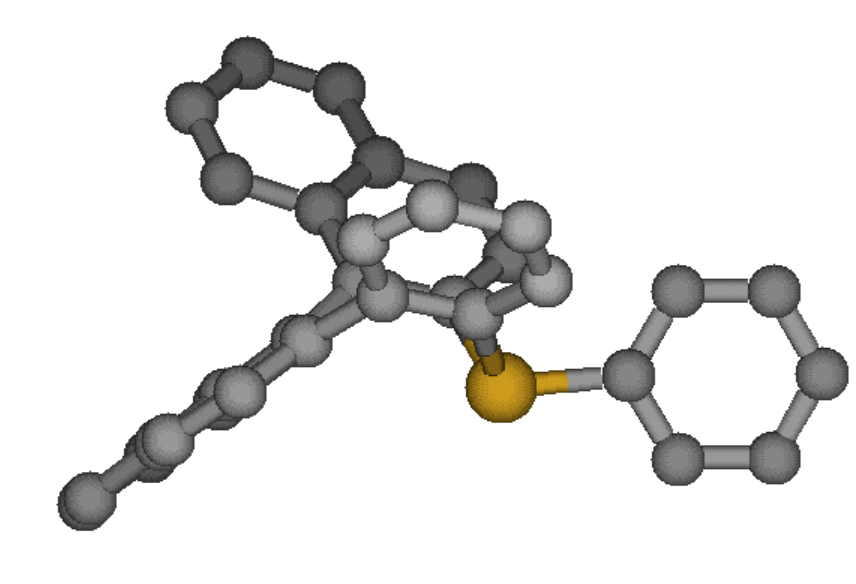

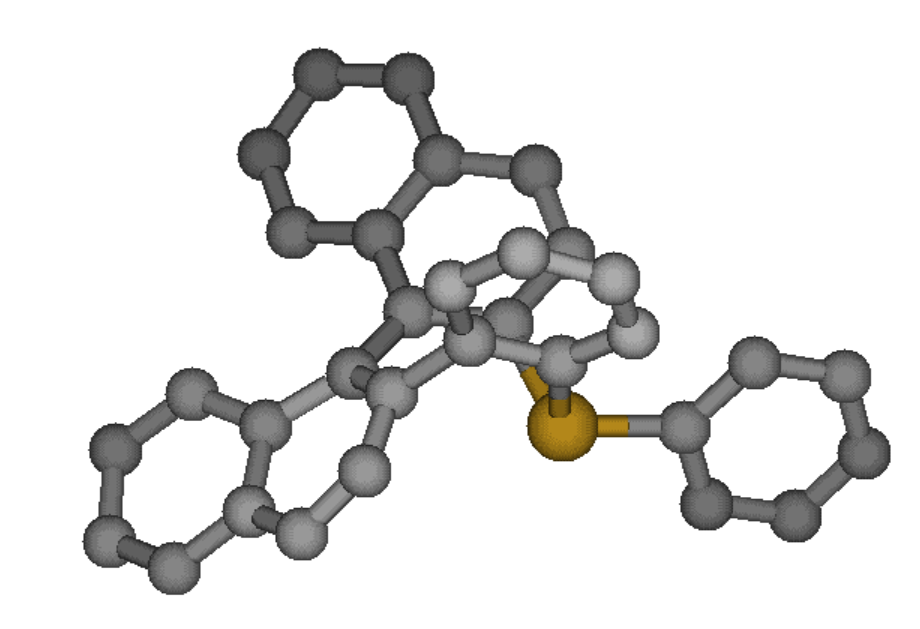


Figure S14: Top (up) and side (down) view of (*M*, R_P_, *M*)-**2** and (*P*, R_P_, *M*)-**2** and the transition state for backbone inversion, optimized at the B3LYP-D3/cc-pVDZ level (Hydrogen atoms were omitted for clarity.)

(*M*, S_P_, *M*)-**2** (P, S_P_, *M*)-**2** Transition state


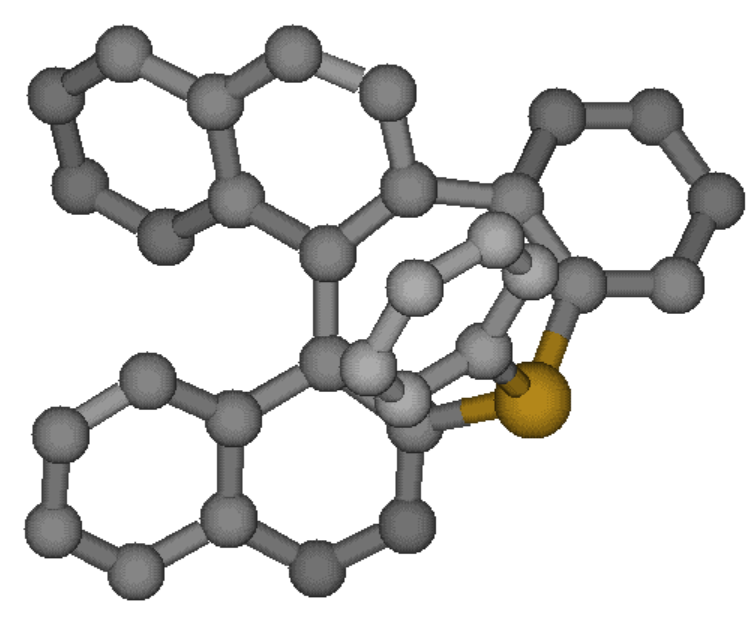

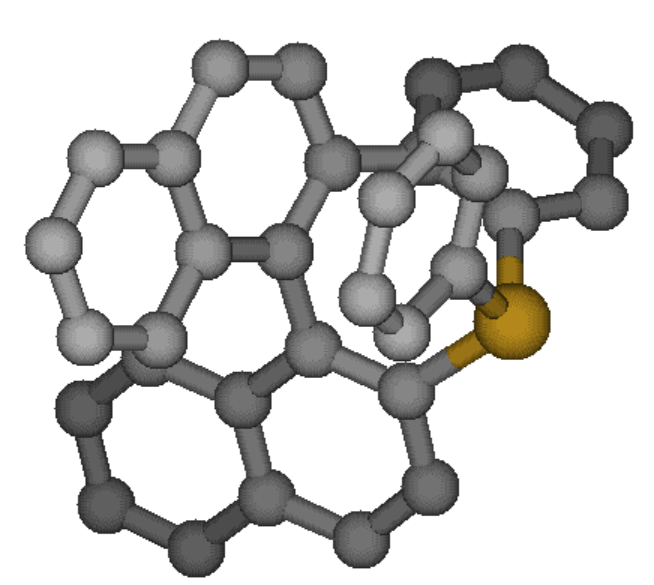

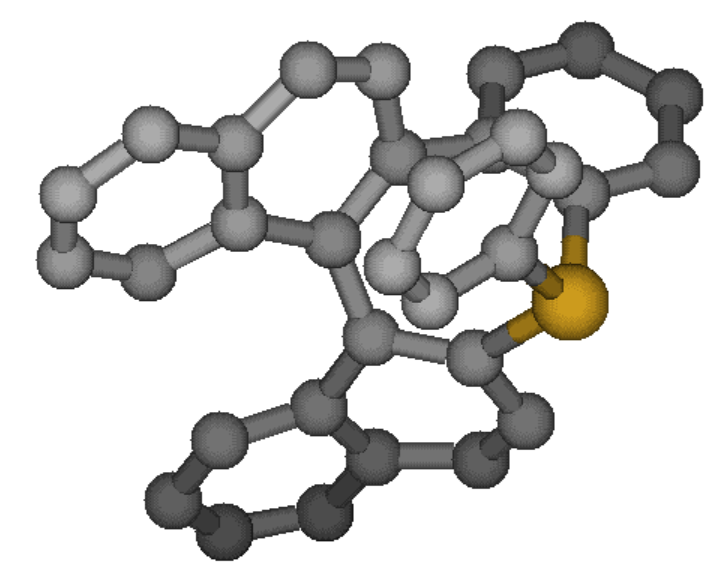


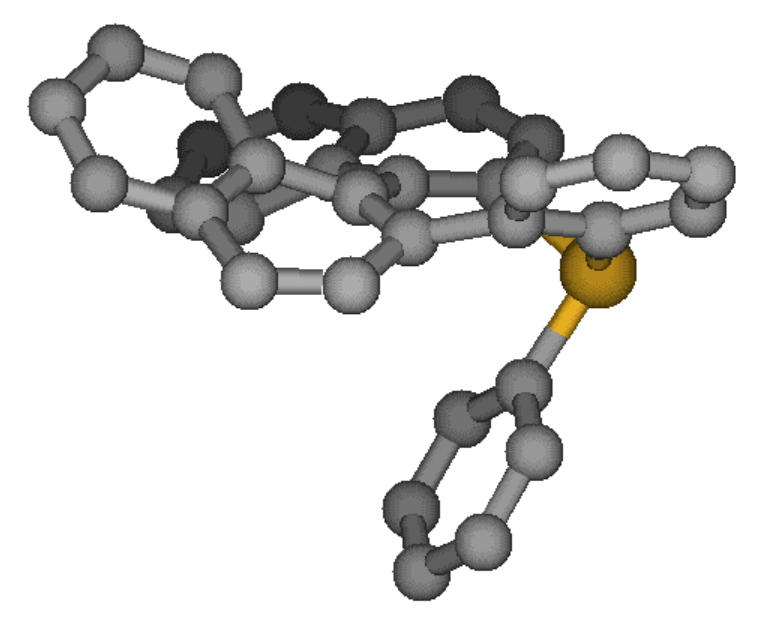

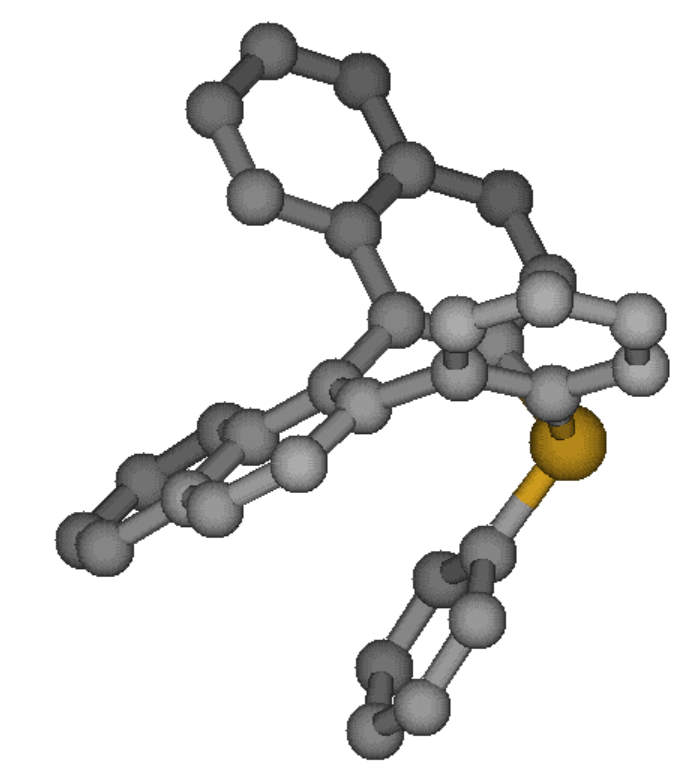

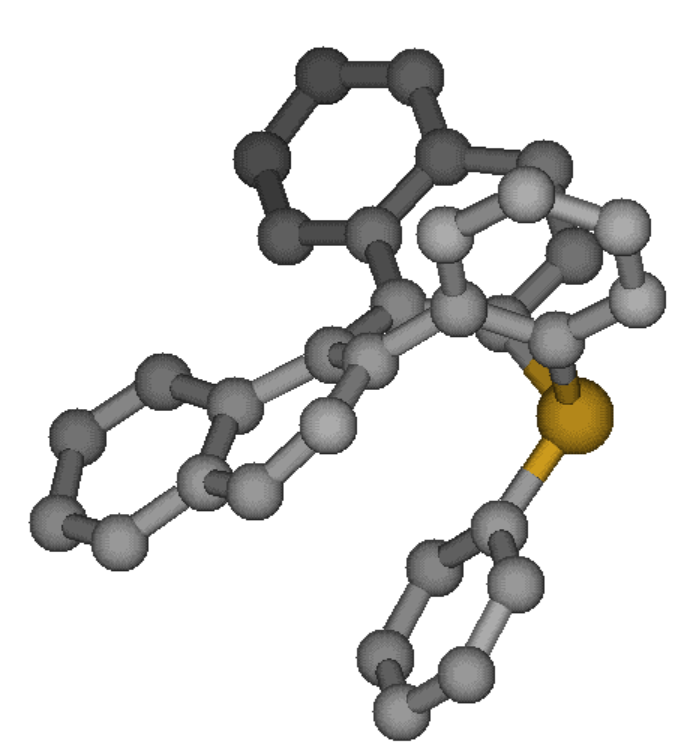


Figure S15: Top (up) and side (down) view of (*M*, S_P_, *M*)-**2** and (P, S_P_, *M*)-**2** and the transition state for backbone inversion, optimized at the B3LYP-D3/cc-pVDZ level (Hydrogen atoms were omitted for clarity.)

Table S4: Different types of isomers for compound **1** and **2** and their relative energies obtained at the B3LYP-D3/cc-pVDZ level


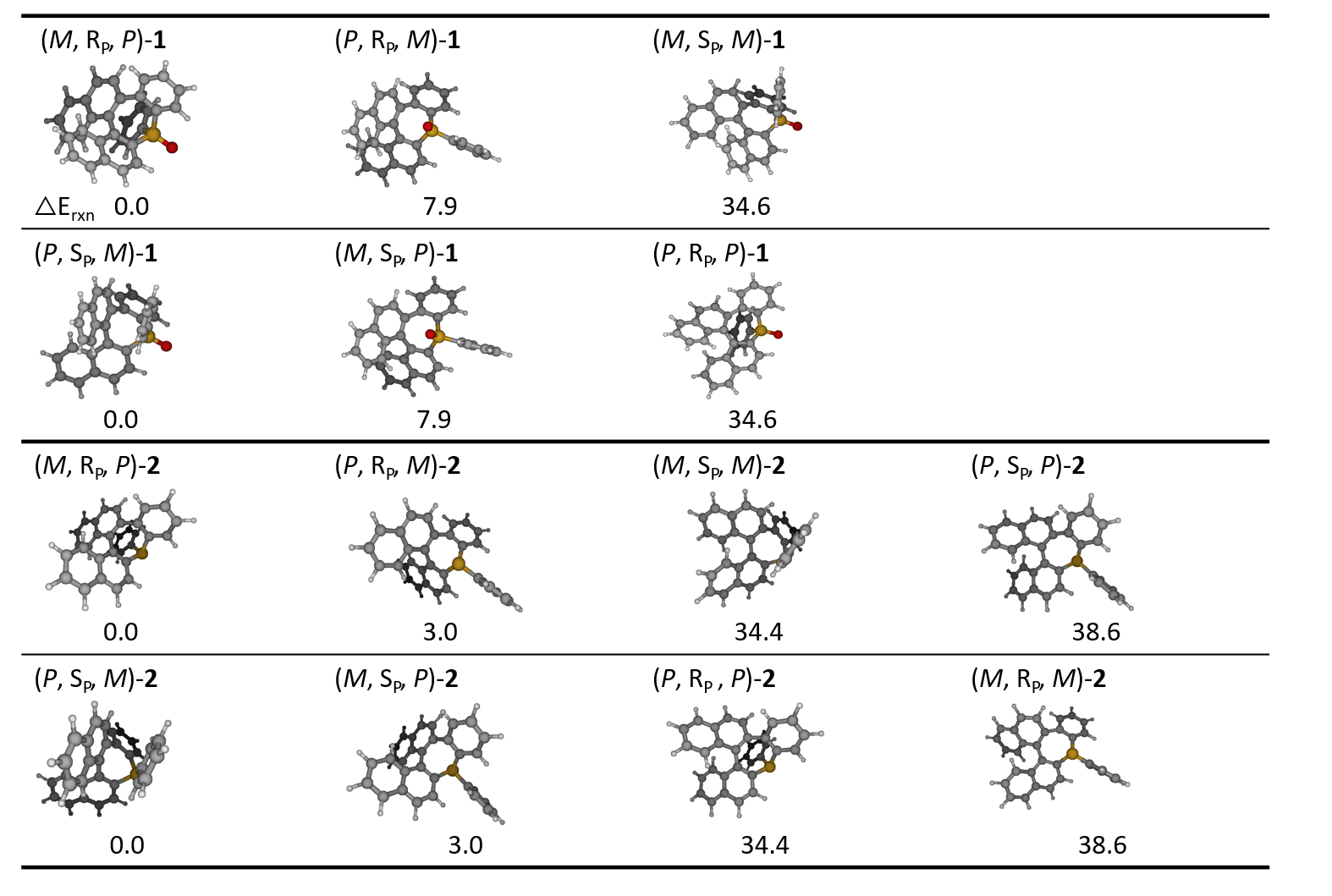


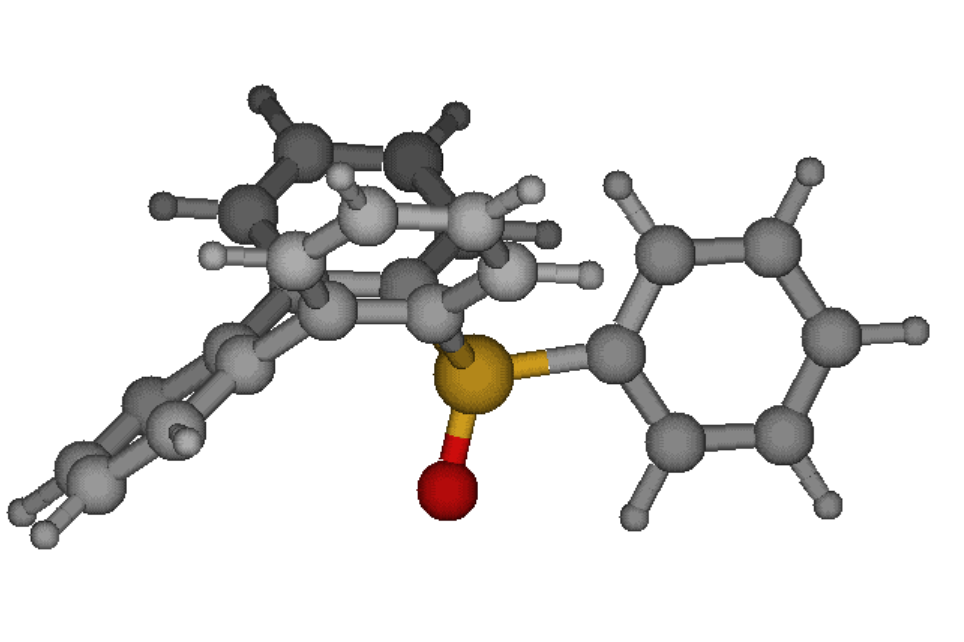

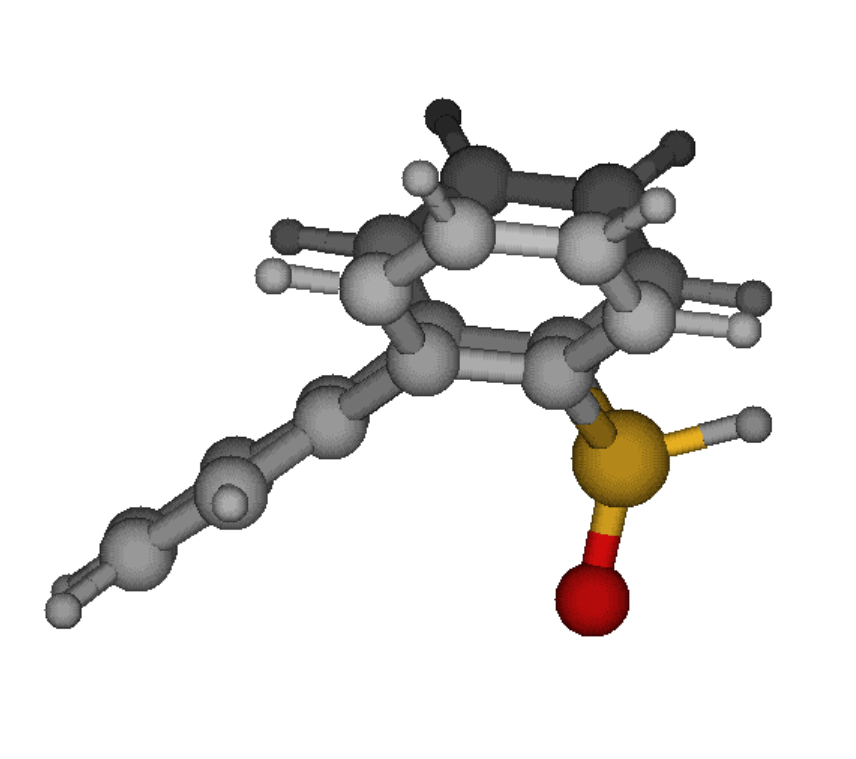

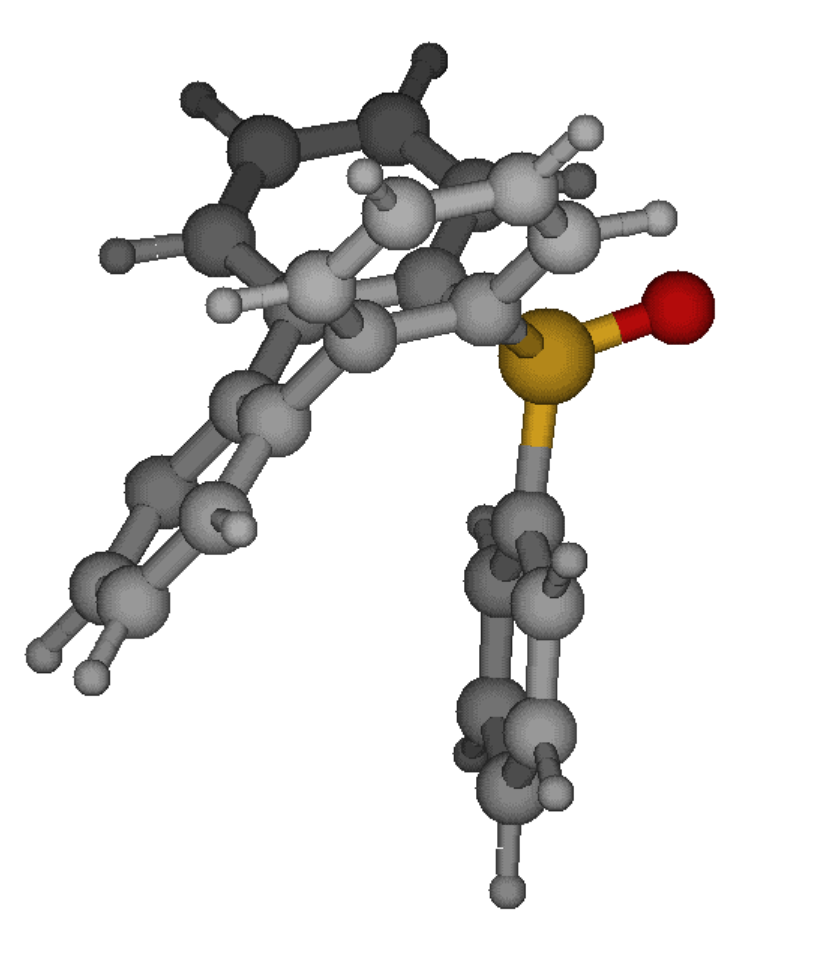

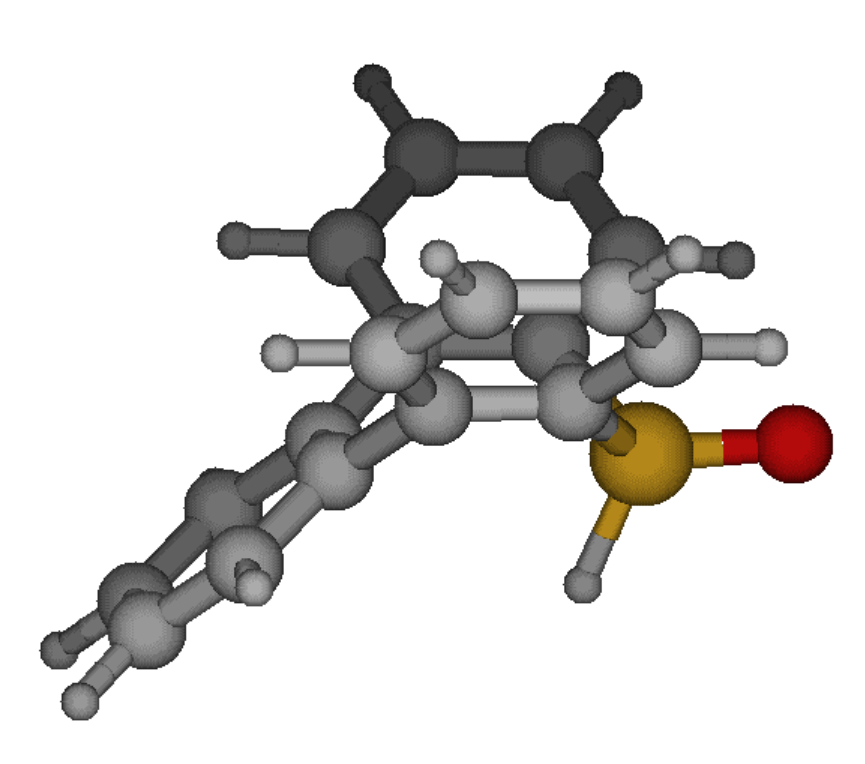


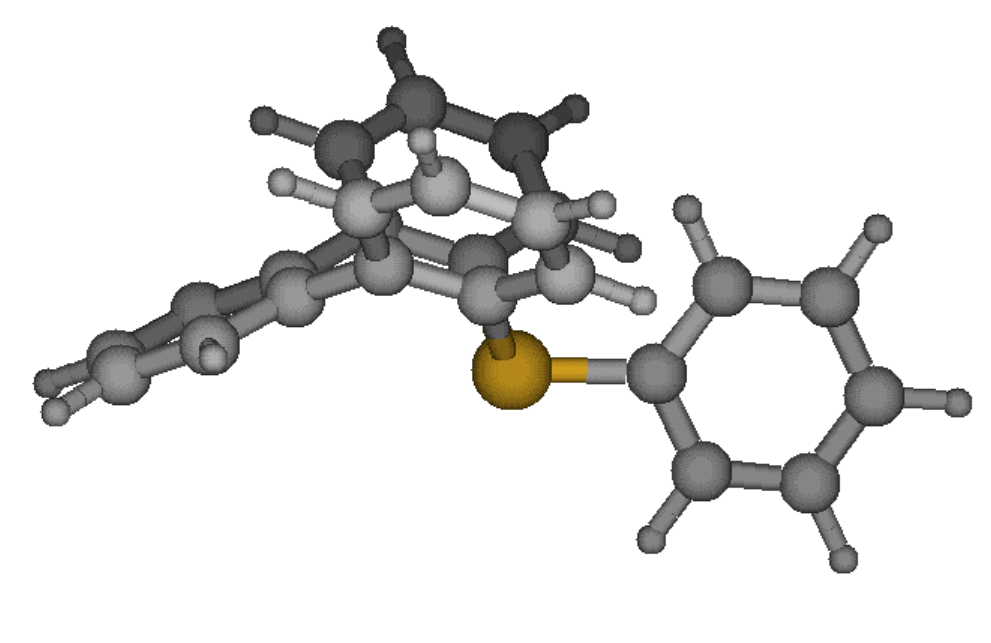

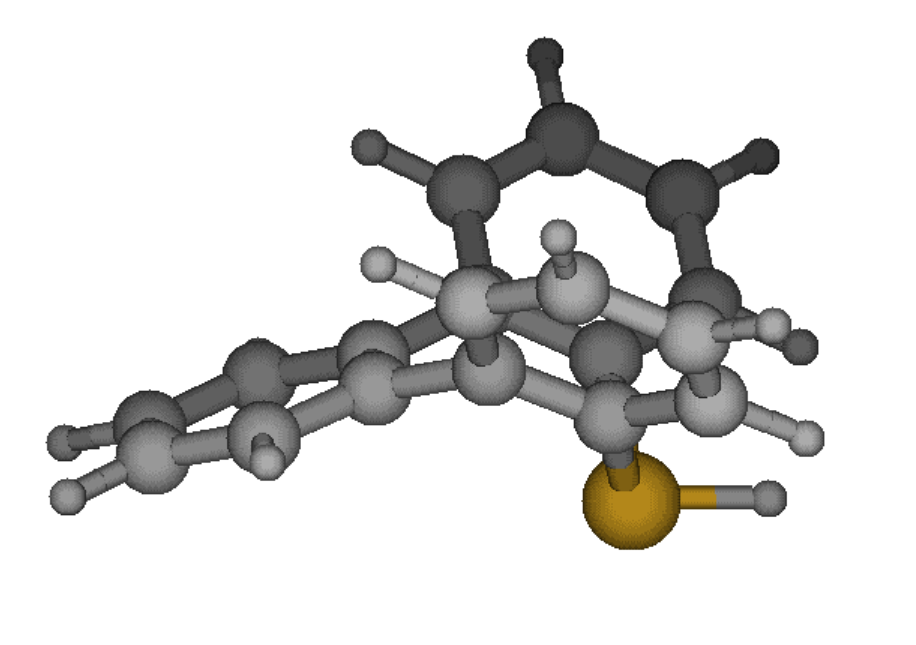

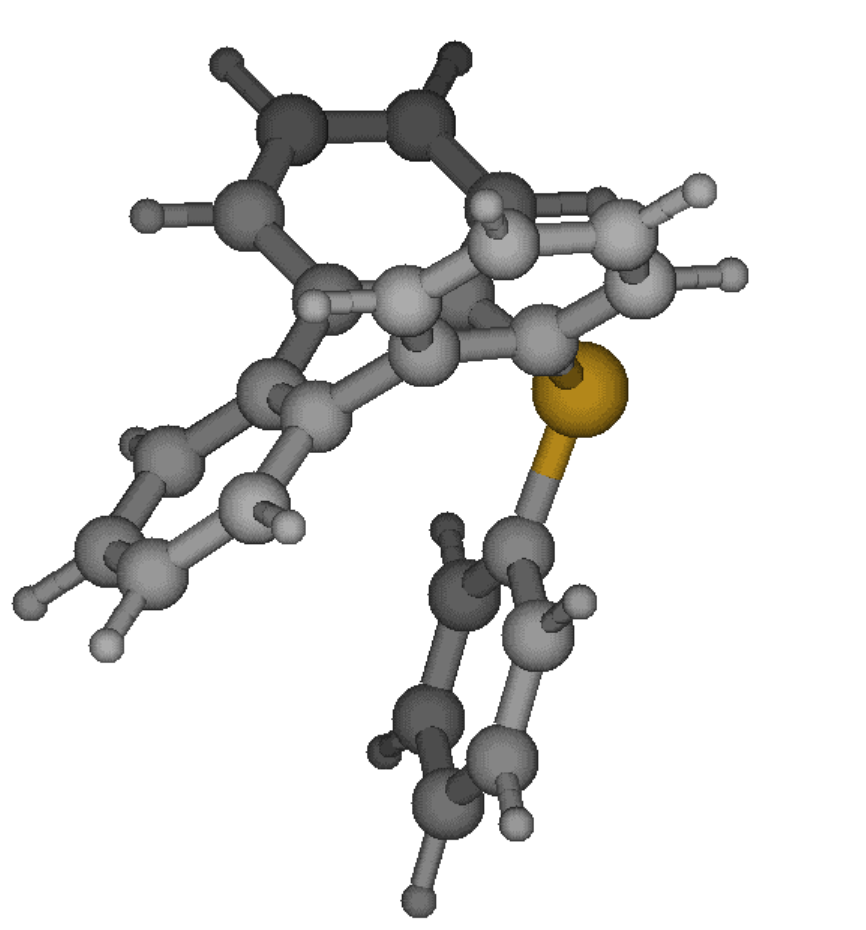

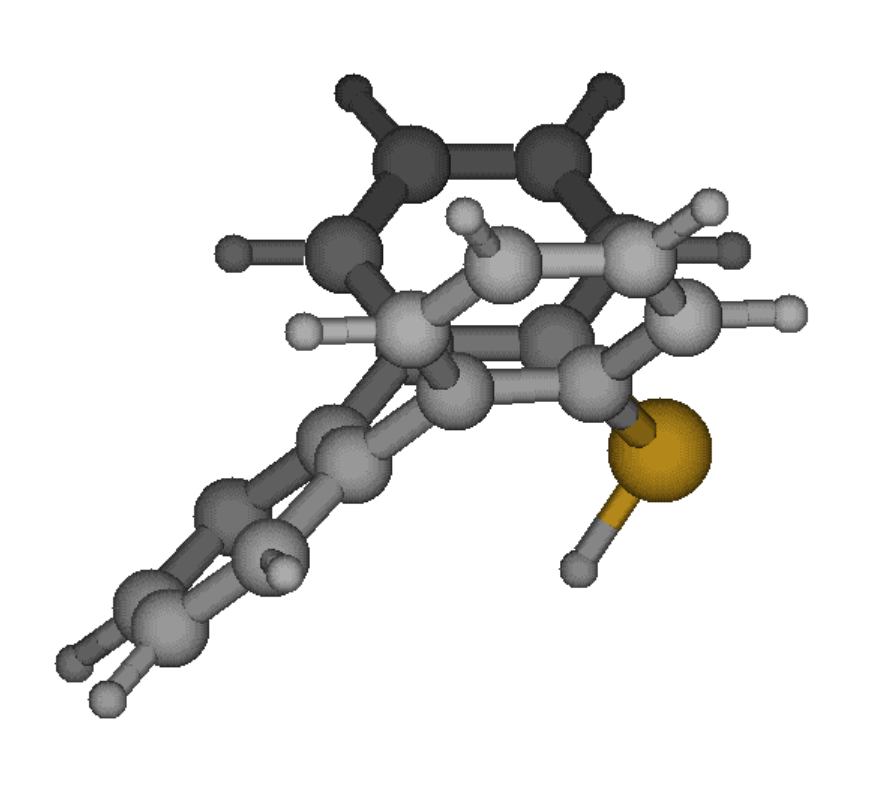


Figure S16: The possible conformational isomers of model molecules tribenzo[*b,d,f*]phosphepine core, optimized at the B3LYP-D3/cc-pVDZ level.

(R_P_,*M*)-**3** (R_P_,*P*)-**3** Transition state


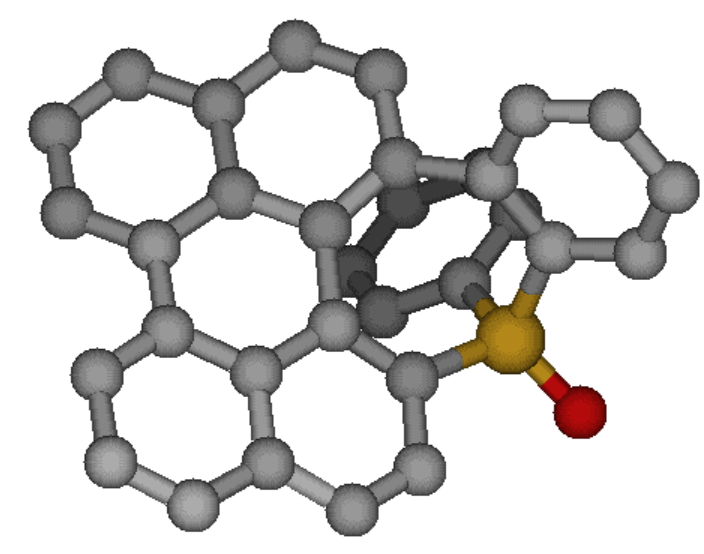

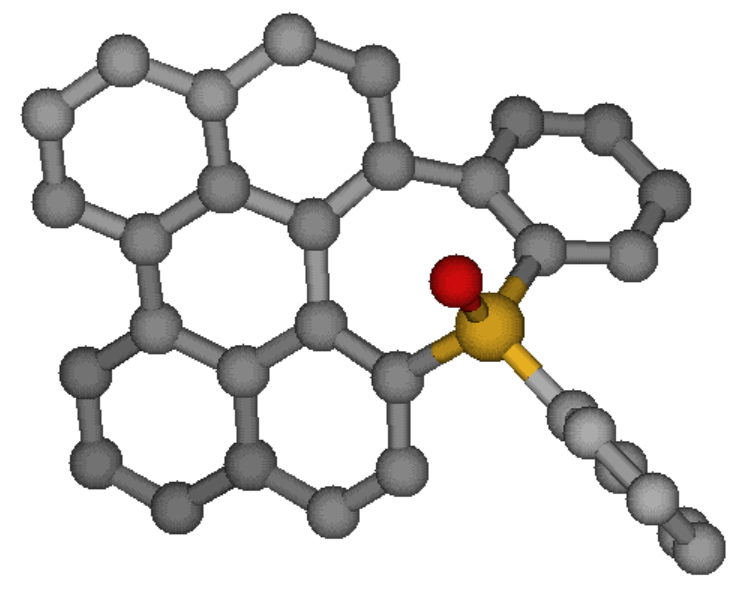

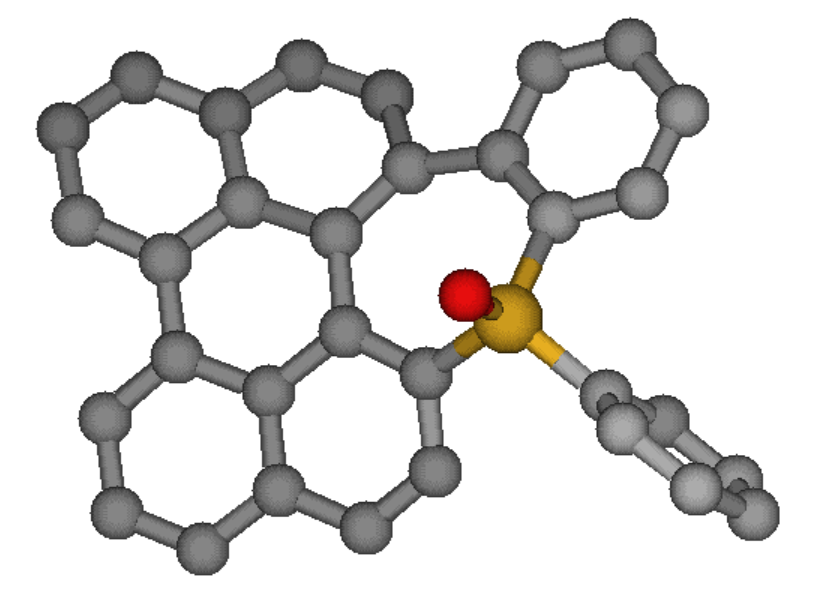


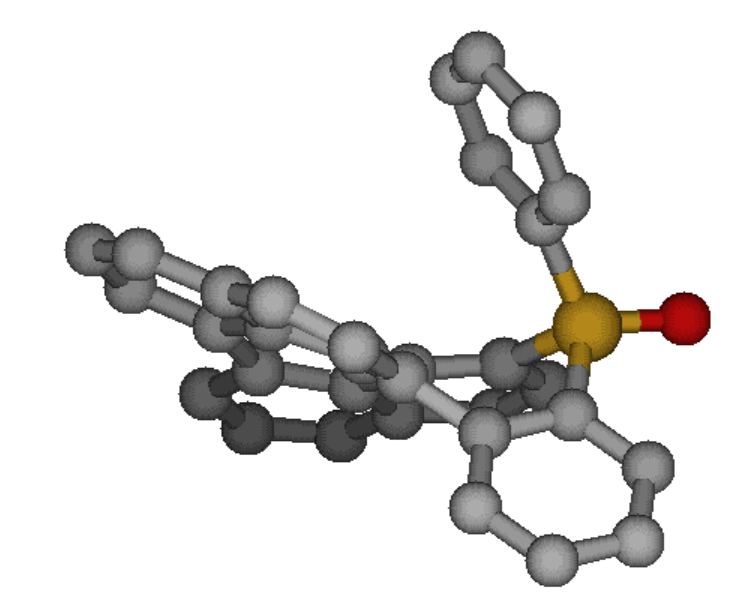

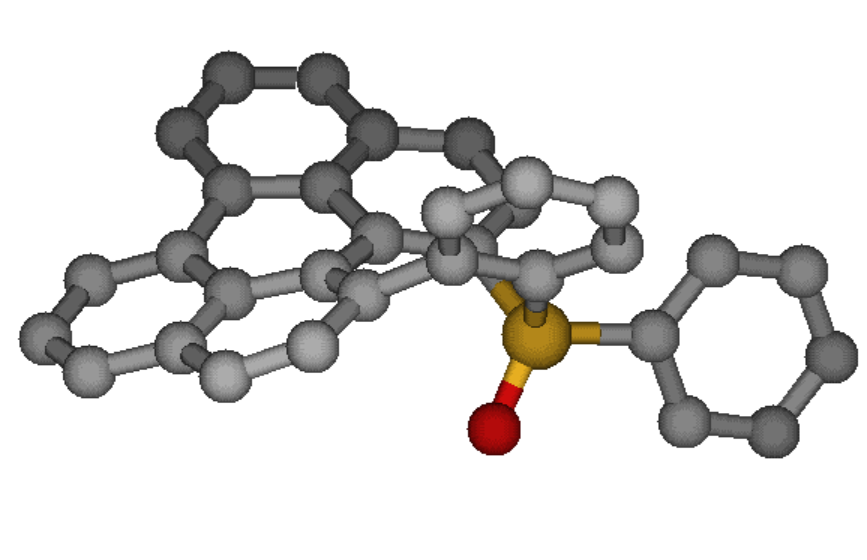

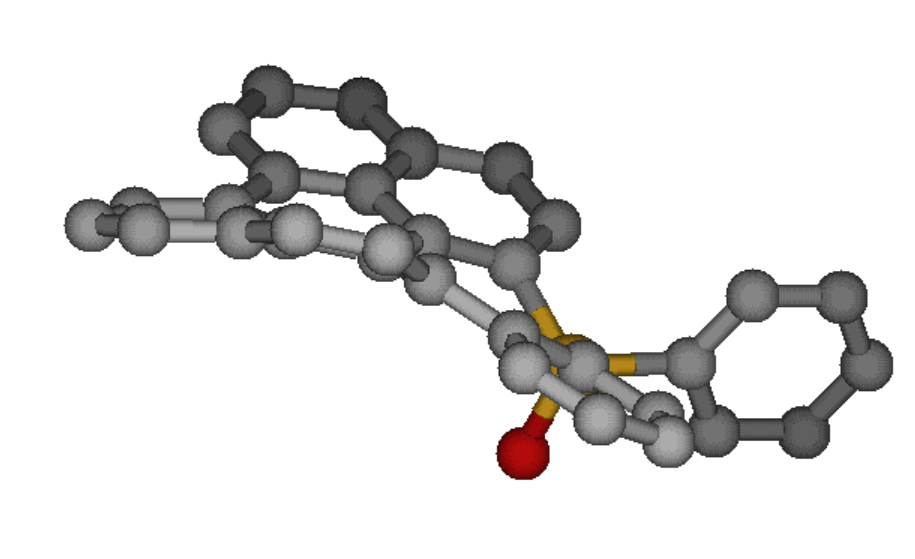


Figure S17: Top (up) and side (down) view of (R_P_,*M*)-**3** and (R_P_,*P*)-**3** and the transition state for backbone inversion, optimized at the B3LYP-D3/cc-pVDZ level (Hydrogen atoms were omitted for clarity.)

(R_P_,*M*)-**4** (S_P_,*M*)-**4** Transition state


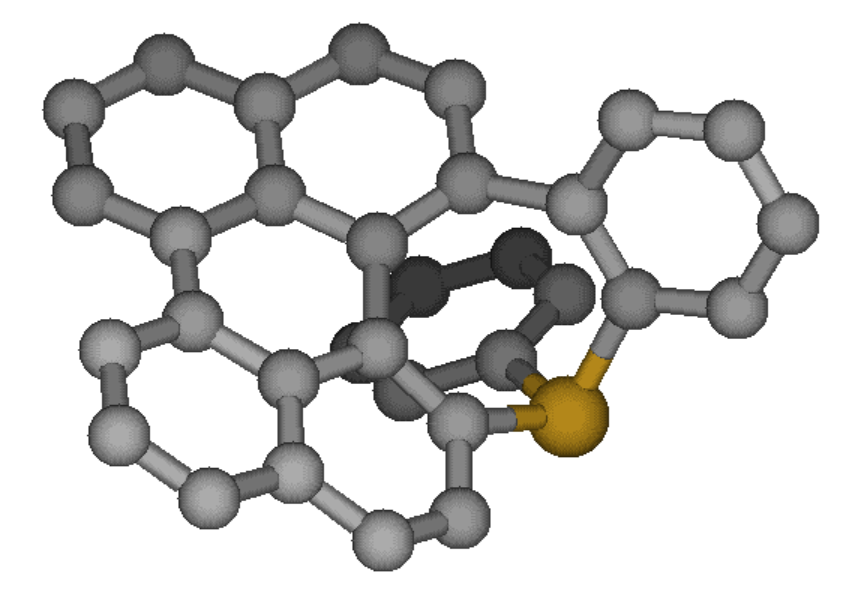

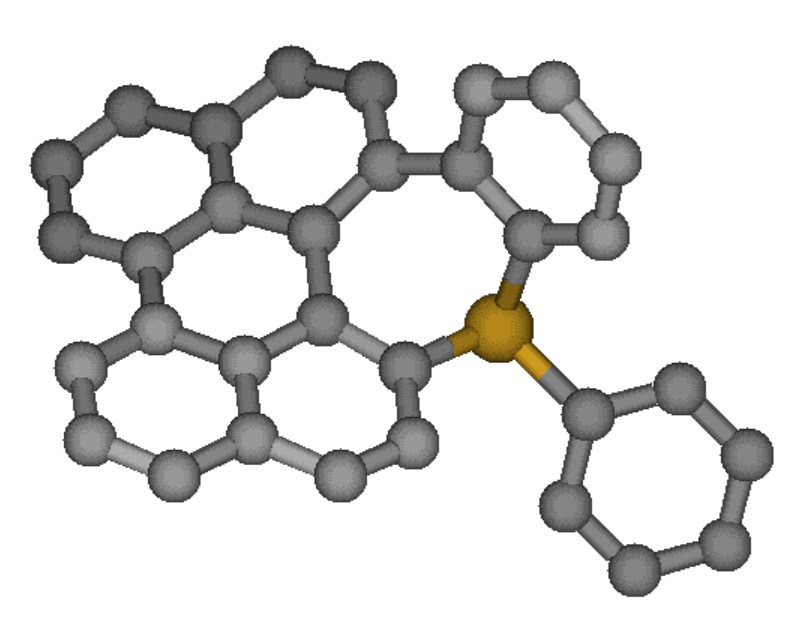

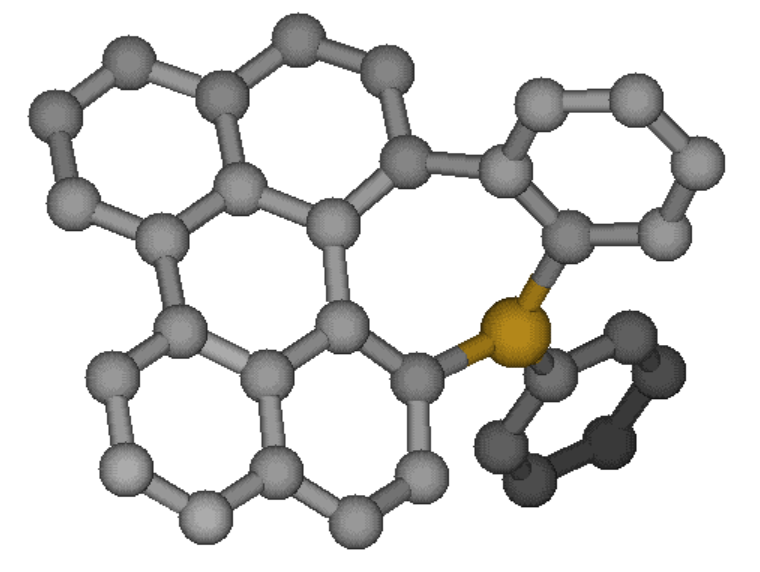


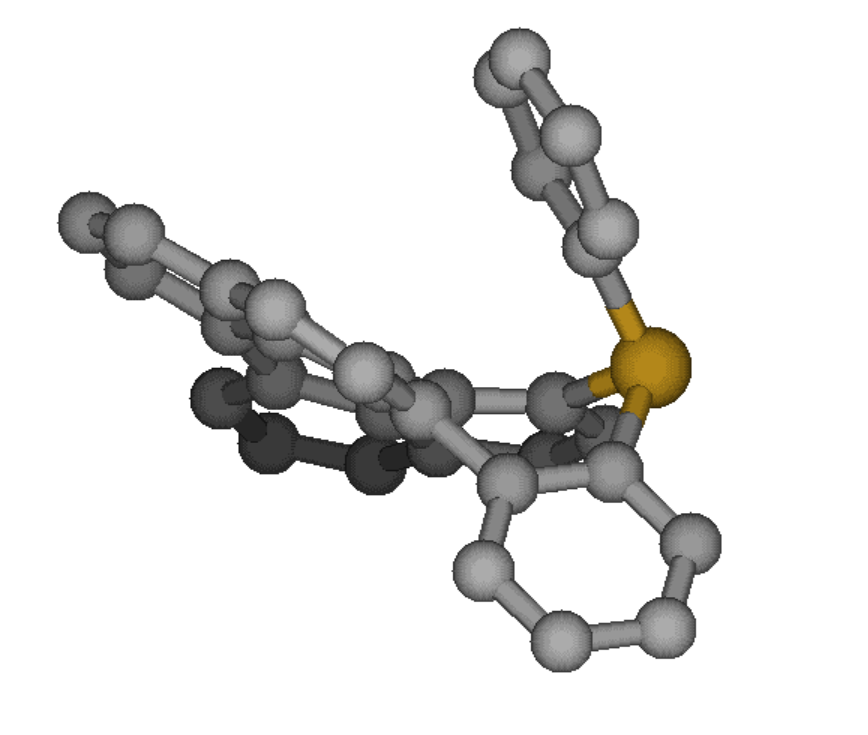

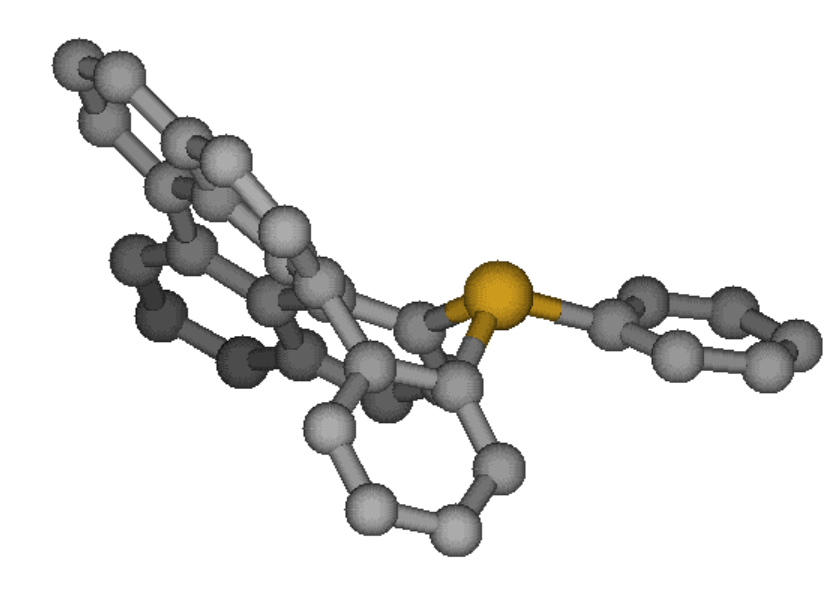

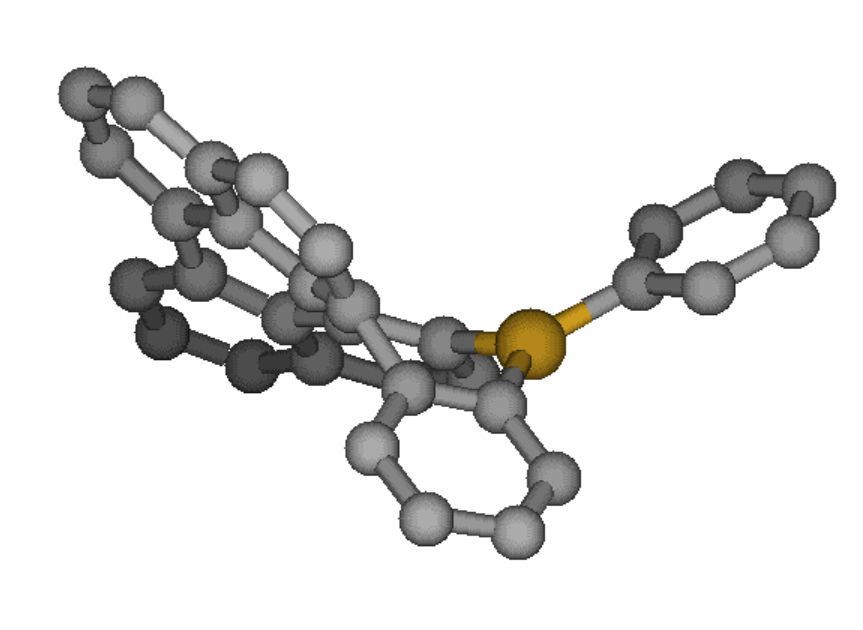


Figure S18: Top (up) and side (down) view of (R_P_,*M*)-**4** and (S_P_,*M*)-**4** and the transition state for the inversion at P-center, optimized at the B3LYP-D3/cc-pVDZ level (Hydrogen atoms were omitted for clarity.)

(R_P_,*M*)-**4** (R_P_,*P*)-**4** Transition state


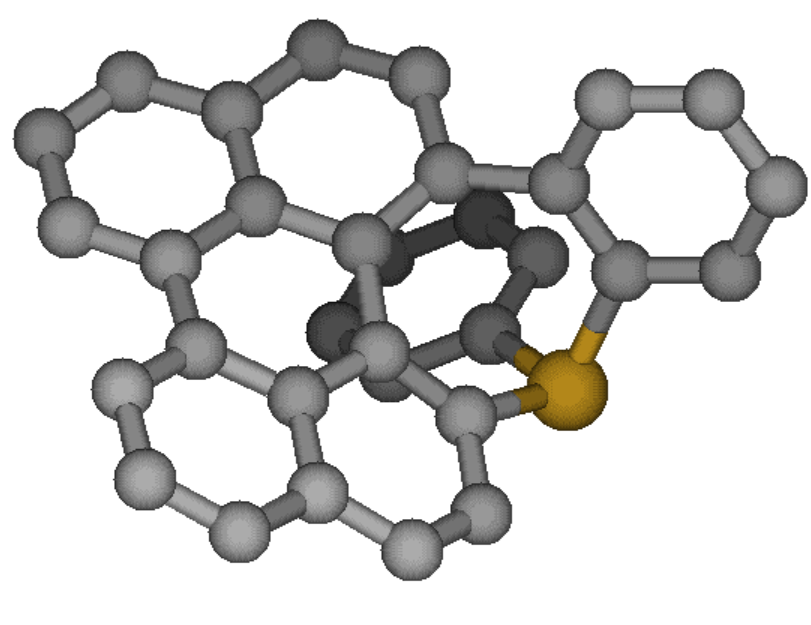

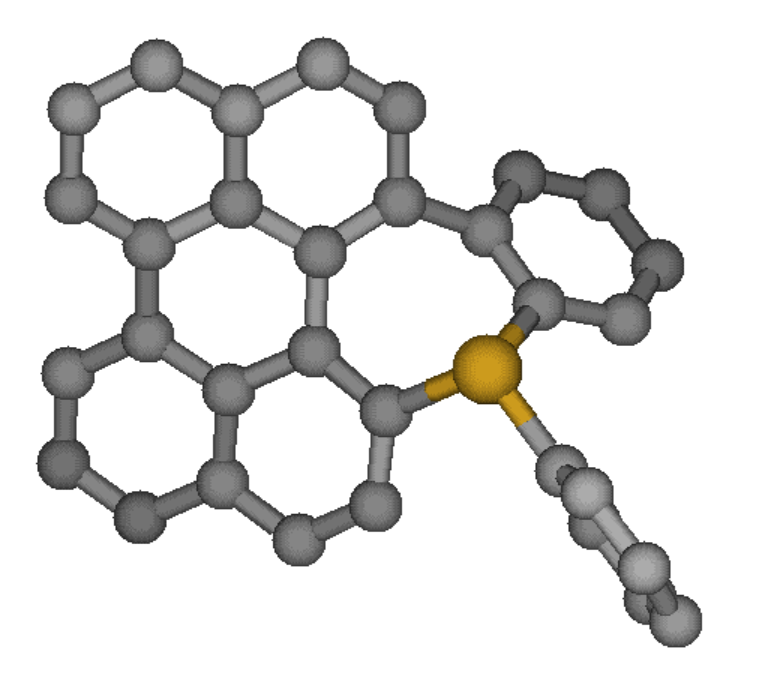

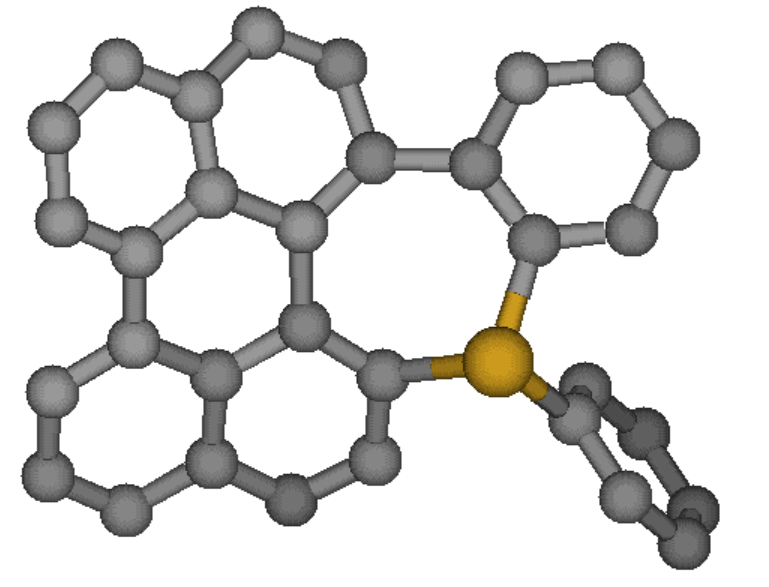


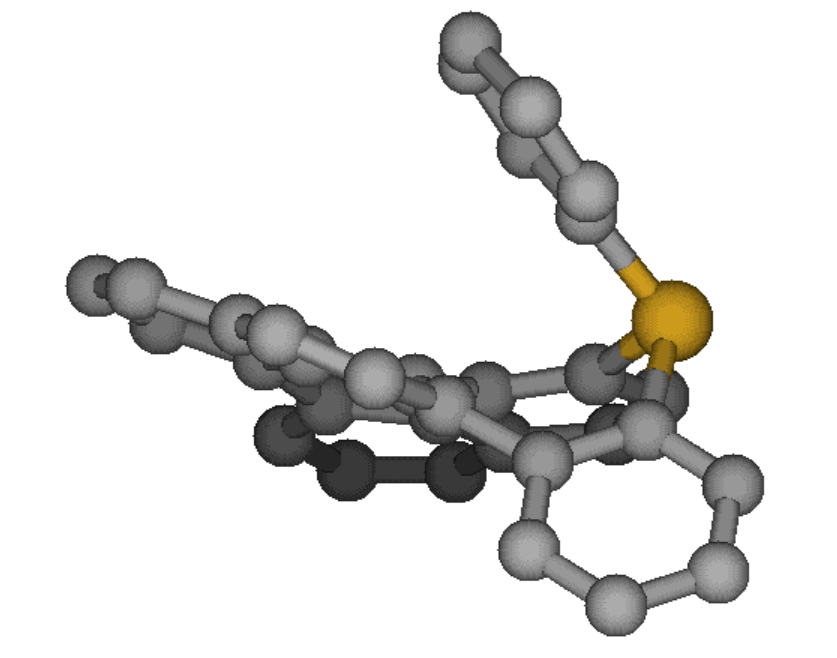

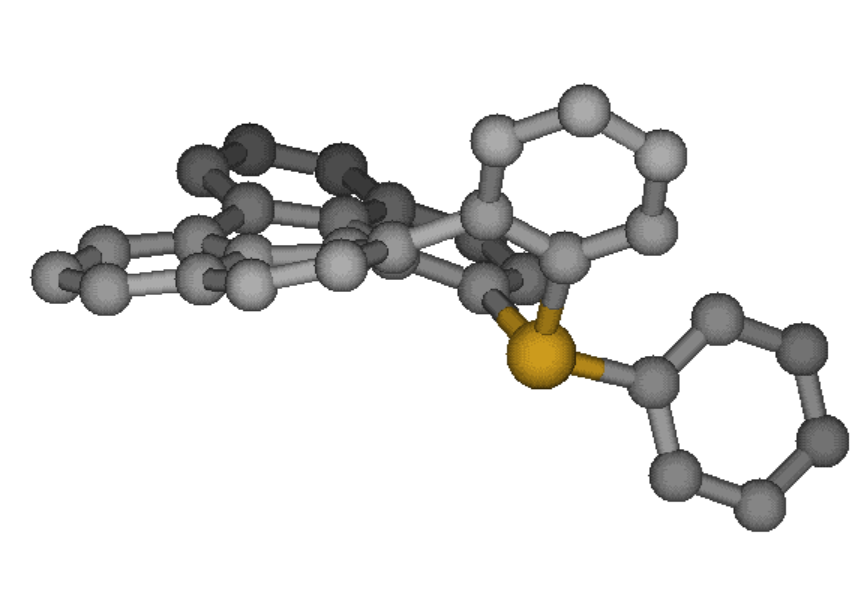

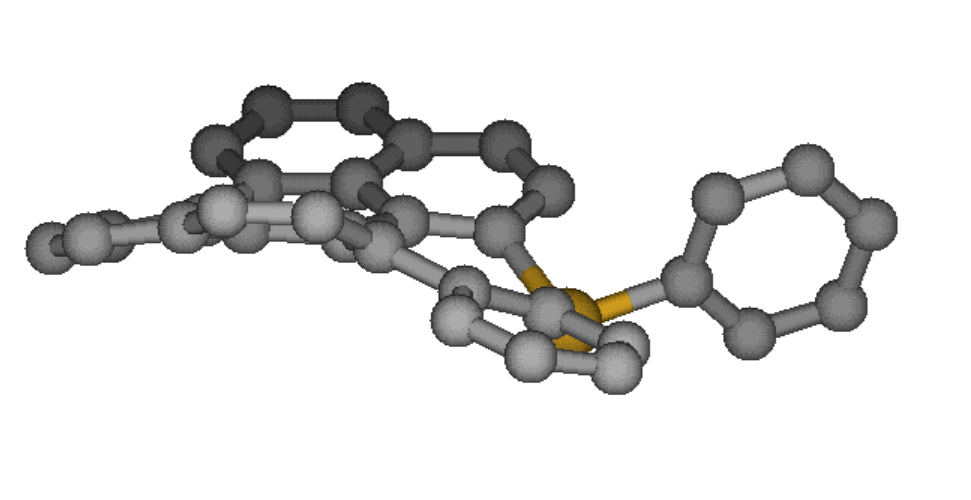


Figure S19: Top (up) and side (down) view of (R_P_,*M*)-**4** and (R_P_,*P*)-**4** and the transition state for backbone inversion, optimized at the B3LYP-D3/cc-pVDZ level (Hydrogen atoms were omitted for clarity.)

**5**


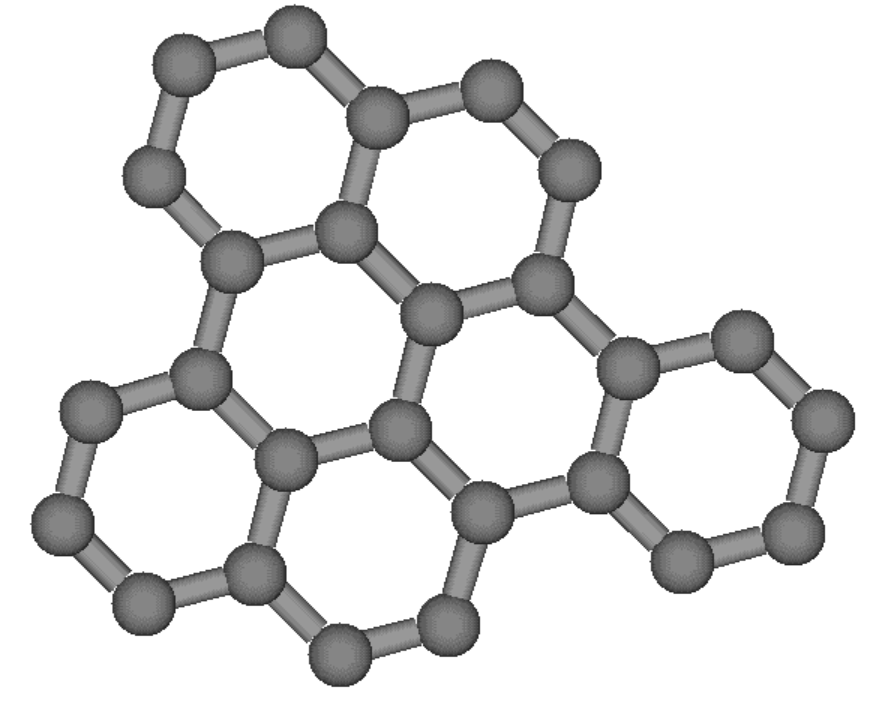


Figure S20: Top view of **5** at the B3LYP-D3/cc-pVDZ level (Hydrogen atoms were omitted for clarity.)

Total energies and Cartesian coordinates at the B3LYP-D3/cc-pVDZ level

(*M*,R_P_,*P*)-**1**

E(hartree): -1648.838004

C -3.204860 0.337803 -0.690463

C -2.016672 0.130217 -1.405469

C -1.496641 1.162338 -2.201269

C -2.150419 2.393691 -2.268986

C -3.330259 2.599702 -1.547146

C -3.858291 1.570044 -0.761710

P -1.158819 -1.487169 -1.362802

O -1.502111 -2.440426 -2.498085

C 0.639323 -1.149402 -1.242729

C 1.166204 -0.394601 -0.198967

C 2.590254 -0.389287 0.001297

C 3.430973 -1.096463 -0.924824

C 2.840579 -1.808182 -2.005236

C 1.473534 -1.855987 -2.147147

C 4.841273 -1.082299 -0.731282

C 5.404993 -0.418476 0.336164

C 4.576135 0.258920 1.265757

C 3.207511 0.274043 1.101839

C 0.277329 0.424268 0.685852

C -0.676904 -0.155046 1.526933

C -1.427610 0.675776 2.412132

C -1.286828 2.040138 2.414591

C -0.400029 2.673516 1.503951

C 0.393985 1.860034 0.631285

C 1.231989 2.520518 -0.313466

C 1.288929 3.897182 -0.380187

C 0.517689 4.694765 0.500137

C -0.309226 4.089997 1.421923

C -1.052813 -1.596983 1.498773

C -1.422025 -2.239385 0.293446

C -1.884057 -3.561377 0.301997

C -1.989937 -4.264172 1.504358

C -1.619303 -3.643612 2.700838

C -1.162421 -2.323523 2.696505

H -2.144476 -4.023097 -0.653089

H -2.352436 -5.294454 1.506431

H -1.683350 -4.189397 3.645040

H -0.872487 -1.846041 3.635232

H -2.160861 0.203735 3.068368

H -1.886546 2.656680 3.088656

H -0.920203 4.692044 2.099361

H 0.574644 5.783865 0.440864

H 1.932105 4.375819 -1.122003

H 1.826270 1.925732 -1.005656

H 2.580531 0.795000 1.825137

H 5.025780 0.770322 2.119714

H 6.488555 -0.418931 0.473957

H 5.470809 -1.618360 -1.446054

H 3.490203 -2.336992 -2.707386

H 0.995410 -2.436005 -2.939120

H -3.608986 -0.458843 -0.061696

H -4.777926 1.729978 -0.194304

H -3.836776 3.566489 -1.593113

H -1.730189 3.198618 -2.875595

H -0.565336 1.009879 -2.751425

(*P*,R_P_,*M*)-**1**

E(hartree): -1648.825351

C -0.292002 -2.196060 2.890958

C -0.310050 -1.685191 1.582228

C -1.547868 -1.257599 1.036669

C -2.720908 -1.406388 1.791506

C -2.683471 -1.926871 3.087646

C -1.461242 -2.305972 3.645139

C 0.937285 -1.720809 0.768781

C 1.418775 -0.638518 0.031475

C 2.541595 -0.823619 -0.852756

C 3.217915 -2.086910 -0.886015

C 2.740309 -3.143297 -0.065919

C 1.627376 -2.968596 0.716630

C 2.999771 0.198846 -1.733969

C 4.076869 -0.007093 -2.570267

C 4.761724 -1.247020 -2.573403

C 4.333458 -2.265298 -1.749989

C 0.807730 0.723914 0.126495

C -0.496658 0.962189 -0.309098

C -0.996913 2.291176 -0.383937

C -0.230917 3.357197 0.027332

C 1.060658 3.150697 0.579812

C 1.590379 1.818188 0.635762

C 1.838910 4.233075 1.078407

C 3.081586 4.015097 1.631335

C 3.598116 2.697076 1.712432

C 2.875204 1.629615 1.224875

P -1.548544 -0.514695 -0.659343

C -3.252210 0.101796 -0.971546

O -1.074438 -1.427618 -1.776541

H -3.682429 -1.123595 1.363158

H -3.609179 -2.034014 3.657270

H -1.416396 -2.697788 4.663775

H 0.662151 -2.507809 3.321345

H 1.233097 -3.805288 1.295747

H 3.250809 -4.109167 -0.092464

H 4.839115 -3.234340 -1.755229

H 5.616785 -1.396131 -3.236473

H 4.399687 0.791716 -3.241621

H 2.479490 1.155290 -1.757697

H 3.282609 0.621559 1.294815

H 4.576799 2.526470 2.166274

H 3.668088 4.853158 2.014442

H 1.426071 5.243446 1.020582

H -0.617488 4.376445 -0.051726

H -1.992865 2.470353 -0.787959

C -3.839257 -0.342901 -2.164788

C -5.133042 0.064027 -2.504523

C -5.845817 0.915210 -1.655510

C -5.264894 1.361969 -0.462564

C -3.972721 0.957885 -0.119785

H -3.262285 -1.008078 -2.810708

H -5.585103 -0.285618 -3.435528

H -6.856893 1.232809 -1.921285

H -5.820441 2.026999 0.202708

H -3.526086 1.310078 0.812554

Transition state of **1**

E(hartree): -1648.764348

C -3.432815 -0.583469 -1.167551

C -2.067243 -0.367004 -1.409810

C -1.639467 0.858997 -1.934640

C -2.569047 1.869534 -2.193376

C -3.927387 1.655948 -1.941225

C -4.359251 0.426321 -1.431580

P -0.920996 -1.764161 -1.137950

O -1.067226 -2.861739 -2.182499

C 0.828058 -1.172612 -0.929308

C 1.327832 -0.069251 -0.200945

C 2.784636 0.111742 -0.312340

C 3.608865 -1.020823 -0.660105

C 3.016425 -2.183546 -1.212048

C 1.673349 -2.188943 -1.463388

C 5.027905 -0.936226 -0.582728

C 5.664159 0.256525 -0.324302

C 4.874882 1.421176 -0.217569

C 3.495107 1.341263 -0.222398

C 0.327250 0.703427 0.664198

C -0.684210 -0.035529 1.314076

C -1.912916 0.573404 1.723679

C -2.197770 1.873404 1.423476

C -1.145316 2.715951 0.983250

C 0.170431 2.164903 0.785124

C 1.217486 3.124263 0.854442

C 0.994226 4.489610 0.835568

C -0.317674 5.002544 0.767627

C -1.366591 4.117263 0.886596

C -0.722615 -1.511729 1.584114

C -1.084826 -2.398365 0.559190

C -1.302968 -3.755135 0.809448

C -1.177829 -4.237604 2.115937

C -0.817158 -3.363936 3.147658

C -0.600138 -2.004901 2.888913

H -1.543458 -4.413909 -0.027959

H -1.348253 -5.295371 2.328528

H -0.703028 -3.742050 4.166324

H -0.334856 -1.323269 3.700163

H -2.671148 -0.068759 2.172851

H -3.192706 2.290078 1.591489

H -2.394309 4.479491 0.970621

H -0.495106 6.078321 0.709187

H 1.845389 5.170077 0.912248

H 2.222703 2.783264 1.050552

H 2.943114 2.265645 -0.272688

H 5.351514 2.402886 -0.165889

H 6.753719 0.313657 -0.281386

H 5.605302 -1.840194 -0.792332

H 3.649915 -3.022614 -1.509384

H 1.199459 -3.007698 -2.006725

H -3.769126 -1.542653 -0.766479

H -5.420579 0.254767 -1.237849

H -4.651561 2.448741 -2.142892

H -2.227708 2.829989 -2.585243

H -0.578882 1.032717 -2.123801

(*M*,R_P_,*M*)-**1**

55

scf done: -1648.782842

C 0.914915 -2.146291 1.850041

C 1.894471 -1.356983 1.225829

C 2.802145 -0.632756 2.013211

C 2.725938 -0.689937 3.407187

C 1.744120 -1.471527 4.023575

C 0.840760 -2.200998 3.243286

P 2.039847 -1.350797 -0.609388

C 2.691948 0.271309 -1.041611

C 2.013532 1.440698 -0.658534

C 2.593265 2.667050 -1.060921

C 3.782700 2.711404 -1.788889

C 4.439358 1.531235 -2.150173

C 3.883386 0.308570 -1.776655

C 0.711440 1.546477 0.073355

C -0.519020 0.889614 -0.226826

C -1.696689 1.754192 -0.108303

C -1.703641 2.860942 0.800445

C -0.492499 3.191746 1.463417

C 0.672033 2.621139 1.019955

C -2.879251 3.644082 0.959108

C -3.985150 3.428669 0.164849

C -3.936451 2.428693 -0.834643

C -2.831920 1.610130 -0.958308

C -0.716581 -0.515804 -0.654925

C -2.015079 -1.167468 -0.475993

C -2.337532 -2.337377 -1.246668

C -1.328533 -2.954797 -2.030366

C -0.024738 -2.543431 -1.878108

C 0.294308 -1.368599 -1.151580

C -3.629102 -2.927418 -1.131349

C -4.535812 -2.474636 -0.199905

C -4.164169 -1.425820 0.675454

C -2.949311 -0.788466 0.534119

H 4.354312 -0.641429 -2.038051

H 5.369854 1.564511 -2.720674

H 4.189683 3.680402 -2.087245

H 2.080434 3.601779 -0.830240

H 1.628252 2.979190 1.405011

H -0.482605 3.966348 2.233343

H -2.868753 4.454426 1.692432

H -4.876367 4.049981 0.276339

H -4.777294 2.302231 -1.519862

H -2.819822 0.852061 -1.739418

H -2.687390 0.002869 1.232516

H -4.840037 -1.119985 1.476871

H -5.514742 -2.949213 -0.103417

H -3.864048 -3.785569 -1.765929

H -1.576223 -3.825462 -2.642019

H 0.807479 -3.123627 -2.281783

H 3.564427 -0.012670 1.536305

H 3.432556 -0.118268 4.013297

H 1.681562 -1.511526 5.113577

H 0.072045 -2.812496 3.721191

H 0.200507 -2.711204 1.247637

O 2.846926 -2.500282 -1.194937

(M,R_P_,*P*)-**2**

54

scf done: -1573.580986

C -0.352704 -2.436565 2.642914

C -0.350366 -1.843253 1.368460

C -1.579088 -1.410651 0.806280

C -2.768727 -1.662595 1.508137

C -2.753228 -2.268584 2.767111

C -1.538716 -2.637079 3.348942

C 0.917697 -1.804634 0.587470

C 1.392387 -0.664268 -0.060944

C 2.548479 -0.762358 -0.919468

C 3.255266 -2.005727 -1.018408

C 2.779646 -3.126784 -0.285649

C 1.640845 -3.031147 0.473233

C 3.012233 0.330882 -1.707738

C 4.119868 0.207378 -2.520852

C 4.830797 -1.015579 -2.591446

C 4.400373 -2.099115 -1.856571

C 0.743122 0.672784 0.114146

C -0.556235 0.899574 -0.340878

C -1.074621 2.224456 -0.363433

C -0.345296 3.279620 0.134438

C 0.928942 3.069559 0.724574

C 1.483918 1.746876 0.719998

C 2.752508 1.549813 1.340351

C 3.436085 2.600916 1.914125

C 2.894096 3.910760 1.894893

C 1.665365 4.135330 1.313222

P -1.521425 -0.606951 -0.886288

C -3.246491 0.054506 -1.092965

C -3.889604 -0.250663 -2.303682

C -5.188532 0.201747 -2.562139

C -5.863435 0.967674 -1.609405

C -5.236120 1.280137 -0.397112

C -3.940501 0.828175 -0.140963

H -3.727667 -1.388960 1.068699

H -3.694573 -2.447102 3.292190

H -1.514131 -3.092148 4.341519

H 0.599504 -2.742970 3.082072

H 1.253373 -3.911857 0.988569

H 3.316417 -4.075952 -0.359892

H 4.929247 -3.054122 -1.912848

H 5.709898 -1.099158 -3.234320

H 4.448548 1.060241 -3.118941

H 2.474719 1.277518 -1.673206

H 3.179850 0.547602 1.366338

H 4.402872 2.422379 2.390228

H 3.448590 4.735815 2.347611

H 1.231530 5.138660 1.301451

H -0.751935 4.293760 0.097646

H -2.058729 2.411150 -0.792009

H -3.364659 -0.849999 -3.051873

H -5.671668 -0.046234 -3.510094

H -6.877591 1.322227 -1.808218

H -5.759421 1.879212 0.352118

H -3.460598 1.077088 0.807435

(M,R_P_,*P*)-**2**

E(hartree): -1573.585692

P 0.795966 -1.740984 -1.704094

C -0.879633 -0.983309 -1.409205

C -1.183403 -0.197621 -0.296189

C -0.108702 0.335997 0.596060

C 0.711198 -0.507910 1.345850

C 0.730479 -1.988985 1.200613

C 0.894192 -2.617443 -0.061529

C 1.964218 -0.307076 -1.548108

C 1.682190 0.893752 -2.220538

C 2.587931 1.955797 -2.189858

C 3.797149 1.829491 -1.499706

C 4.094652 0.630497 -0.844910

C 3.187620 -0.431817 -0.870974

C 1.655908 0.056149 2.254371

C 1.828974 1.412429 2.363706

C 1.081777 2.300788 1.545815

C 0.097983 1.760649 0.654680

C -0.597993 2.668152 -0.195692

C -0.344003 4.023252 -0.157760

C 0.615810 4.552372 0.739668

C 1.312139 3.703603 1.572453

C -1.910794 -1.391821 -2.300228

C -3.223149 -1.043088 -2.079725

C -3.585042 -0.302647 -0.923570

C -2.559039 0.117045 -0.010678

C -2.956663 0.813036 1.169468

C -4.282102 1.098474 1.417593

C -5.288330 0.707431 0.498311

C -4.943427 0.019246 -0.643776

C 1.027101 -4.014014 -0.119512

C 0.990169 -4.797958 1.036669

C 0.811577 -4.182469 2.276956

C 0.691553 -2.793409 2.352848

H 1.154674 -4.490863 -1.094412

H 1.094576 -5.882951 0.966206

H 0.765100 -4.781774 3.189150

H 0.550468 -2.315616 3.325121

H 2.282594 -0.620357 2.838176

H 2.574077 1.821645 3.050384

H 2.065222 4.095317 2.261084

H 0.806078 5.627691 0.763280

H -0.884824 4.693488 -0.829711

H -1.331918 2.280189 -0.900751

H -2.195172 1.115912 1.887381

H -4.560192 1.628014 2.331564

H -6.334613 0.944875 0.703438

H -5.711603 -0.300141 -1.352781

H -4.004554 -1.351044 -2.779195

H -1.641783 -1.995835 -3.169812

H 0.731716 1.012489 -2.746677

H 2.339116 2.891882 -2.694946

H 4.502227 2.663131 -1.468723

H 5.035037 0.523302 -0.298608

H 3.422241 -1.356150 -0.337389

Transition state of **2** (P-inversion)

E(hartree): -1573.532089

C -0.632115 3.070440 -2.155376

C -0.680110 1.994940 -1.246777

C -1.792007 1.112199 -1.316952

C -2.840645 1.378216 -2.221374

C -2.794049 2.485353 -3.064593

C -1.668259 3.318127 -3.053625

C 0.320507 1.921308 -0.143611

C 0.980855 0.760322 0.264839

C 1.773695 0.782117 1.473431

C 1.960109 2.013757 2.183116

C 1.333605 3.189219 1.690855

C 0.529117 3.135980 0.581473

C 2.368571 -0.392837 2.020588

C 3.116602 -0.345183 3.178659

C 3.319755 0.880891 3.857639

C 2.746880 2.034278 3.367666

C 0.944535 -0.531518 -0.498321

C -0.224958 -1.291127 -0.585515

C -0.189958 -2.635227 -1.065511

C 0.989825 -3.188206 -1.496065

C 2.181966 -2.408640 -1.550768

C 2.158815 -1.058457 -1.064195

C 3.356227 -0.292205 -1.150254

C 4.511583 -0.832069 -1.677894

C 4.534422 -2.169353 -2.142428

C 3.389791 -2.937415 -2.079721

P -1.773183 -0.418332 -0.376534

C -3.134047 -0.994464 0.622994

C -4.334351 -0.256943 0.711222

C -5.390963 -0.731724 1.487890

C -5.282720 -1.939117 2.187258

C -4.090310 -2.667605 2.106250

C -3.022076 -2.207336 1.337315

H -3.683973 0.687000 -2.276478

H -3.617065 2.676553 -3.756760

H -1.600323 4.162219 -3.743243

H 0.236360 3.732647 -2.139239

H 0.003196 4.033660 0.252392

H 1.472500 4.130938 2.227789

H 2.880355 2.986034 3.888400

H 3.919465 0.906782 4.770165

H 3.553097 -1.263802 3.577015

H 2.219225 -1.348597 1.520832

H 3.349845 0.739135 -0.797338

H 5.415406 -0.221794 -1.739566

H 5.455649 -2.587331 -2.554215

H 3.393690 -3.968007 -2.444139

H 1.016900 -4.221000 -1.852809

H -1.118368 -3.208059 -1.107466

H -2.093842 -2.781639 1.298987

H -3.984962 -3.607328 2.654224

H -6.114683 -2.304943 2.791957

H -6.311531 -0.145678 1.547827

H -4.434945 0.691870 0.180377

(*M*, R_P_, *M*)-**2**

E(hartree): -1573.524120

C -1.565315 1.884431 -0.129107

C -0.727336 0.683837 -0.053609

C -1.357525 -0.649868 -0.169488

C -2.778605 -0.791832 0.154613

C -3.408106 -0.002984 1.161893

C -4.748056 -0.145485 1.459536

C -5.550173 -1.069754 0.748794

C -4.963080 -1.896784 -0.184562

C -3.569488 -1.818657 -0.455867

C -2.910216 -2.783606 -1.261700

C -1.541268 -2.853259 -1.205645

C -0.730431 -1.870893 -0.551376

C -1.080174 3.131176 0.387942

C 0.274878 3.216391 0.790848

C 1.130774 2.180990 0.500071

C 0.669501 0.923368 0.014323

C -2.816862 1.927372 -0.814385

C -3.583322 3.073710 -0.859163

C -3.159232 4.255137 -0.205257

C -1.919253 4.281217 0.392289

C 0.599691 -2.394954 -0.100149

C 1.847987 -1.745487 -0.155616

C 3.007005 -2.413132 0.272462

C 2.945877 -3.701648 0.805737

C 1.707790 -4.331262 0.929718

C 0.556960 -3.682361 0.483052

P 1.895281 -0.088008 -0.954265

C 3.538776 0.570489 -0.390383

C 4.389957 1.094216 -1.374785

C 5.648763 1.604551 -1.034640

C 6.068035 1.597551 0.296929

C 5.227038 1.078404 1.290141

C 3.973970 0.568510 0.949708

H 3.976700 -1.924401 0.180399

H 3.861907 -4.201852 1.128003

H 1.630445 -5.327232 1.371332

H -0.408593 -4.175411 0.604182

H -1.023467 -3.692000 -1.674783

H -3.490515 -3.522411 -1.818824

H -5.550121 -2.662170 -0.698686

H -6.616962 -1.152310 0.968152

H -5.189432 0.461169 2.253161

H -2.812985 0.714600 1.724183

H -3.168212 1.044493 -1.343005

H -4.523858 3.066392 -1.414305

H -3.789384 5.147115 -0.217203

H -1.533623 5.201819 0.837904

H 0.654688 4.146082 1.221615

H 2.198729 2.334382 0.642756

H 3.321202 0.165806 1.727269

H 5.550844 1.072172 2.333729

H 7.049298 1.996212 0.565014

H 6.299869 2.008438 -1.813349

H 4.062906 1.102925 -2.417520

(*P*,R_P_,*M*)-**2**

E(hartree): -1573.580986

C -1.483703 1.747069 0.719822

C -0.743172 0.672829 0.113903

C -1.392554 -0.664201 -0.060951

C -2.548765 -0.762304 -0.919339

C -3.012647 0.330869 -1.707602

C -4.120387 0.207310 -2.520586

C -4.831323 -1.015636 -2.591029

C -4.400807 -2.099113 -1.856106

C -3.255601 -2.005669 -1.018100

C -2.779915 -3.126712 -0.285336

C -1.641030 -3.031062 0.473403

C -0.917840 -1.804546 0.587454

C -0.928625 3.069713 0.724071

C 0.345433 3.279606 0.133478

C 1.074550 2.224304 -0.364400

C 0.556101 0.899458 -0.341429

C -2.752091 1.550185 1.340628

C -3.435371 2.601392 1.914575

C -2.893276 3.911187 1.895047

C -1.664743 4.135596 1.312890

C 0.350282 -1.843193 1.368376

C 1.579023 -1.410780 0.806116

C 2.768651 -1.662758 1.507976

C 2.753120 -2.268589 2.767022

C 1.538587 -2.636906 3.348930

C 0.352581 -2.436364 2.642901

P 1.521418 -0.607123 -0.886548

C 3.246499 0.054385 -1.092984

C 3.889968 -0.251144 -2.303421

C 5.189007 0.201098 -2.561619

C 5.863670 0.967235 -1.608891

C 5.236002 1.280074 -0.396870

C 3.940279 0.828275 -0.140987

H 3.727598 -1.389229 1.068482

H 3.694450 -2.447129 3.292120

H 1.513995 -3.091818 4.341579

H -0.599659 -2.742638 3.082081

H -1.253520 -3.911712 0.988813

H -3.316716 -4.075870 -0.359476

H -4.929685 -3.054127 -1.912227

H -5.710502 -1.099270 -3.233786

H -4.449096 1.060159 -3.118681

H -2.475181 1.277537 -1.673252

H -3.179516 0.548013 1.366886

H -4.401987 2.422945 2.391058

H -3.447515 4.736326 2.347919

H -1.230803 5.138878 1.300881

H 0.752118 4.293717 0.096387

H 2.058543 2.410847 -0.793311

H 3.460123 1.077458 0.807207

H 5.759125 1.879285 0.352373

H 6.877926 1.321628 -1.807493

H 5.672414 -0.047164 -3.509363

H 3.365200 -0.850651 -3.051603

Transition state of **2** (backbone 1)

E(hartree): -1573.506225

C -2.720581 2.584646 -0.233250

C -1.416953 2.161536 0.147403

C -0.569571 3.224270 0.624702

C -1.116808 4.488523 0.979416

C -2.439429 4.790267 0.742108

C -3.224195 3.839684 0.056632

C -0.850504 0.812499 -0.015573

C 0.552920 0.772806 -0.194070

C 1.365951 1.910696 0.076558

C 0.837083 3.055458 0.603255

C -1.549646 -0.538339 0.115496

C -0.833744 -1.542867 0.800524

C -1.129374 -2.932620 0.623960

C -2.072663 -3.351754 -0.268167

C -2.960672 -2.395748 -0.825784

C -2.806996 -1.000064 -0.498878

C -4.074774 -2.844518 -1.587609

C -5.113870 -1.999187 -1.908822

C -5.087521 -0.688718 -1.389616

C -3.979122 -0.222328 -0.705865

C 0.343051 -1.375313 1.724664

C 1.632187 -1.248680 1.166524

C 2.758392 -1.416908 1.978641

C 2.603360 -1.637841 3.353698

C 1.327697 -1.693970 3.918465

C 0.197383 -1.587804 3.098637

P 1.463390 -0.826712 -0.632590

C 3.142703 -0.276136 -1.218966

C 3.375356 -0.505130 -2.587845

C 4.595759 -0.170234 -3.181573

C 5.618026 0.387867 -2.410076

C 5.408471 0.615855 -1.046120

C 4.183471 0.291847 -0.457470

H 3.759668 -1.386323 1.549141

H 3.487709 -1.762458 3.983193

H 1.208141 -1.847254 4.993276

H -0.804624 -1.689500 3.521762

H -0.506983 -3.654733 1.153992

H -2.207983 -4.411850 -0.494597

H -4.112282 -3.901103 -1.865226

H -5.971442 -2.356739 -2.482420

H -5.960796 -0.040455 -1.493465

H -4.063462 0.733546 -0.213281

H -3.307424 1.948804 -0.876888

H -4.228480 4.102170 -0.284336

H -2.848449 5.767814 1.005629

H -0.441305 5.239819 1.396760

H 1.483194 3.876455 0.923963

H 2.440202 1.851611 -0.072756

H 4.038517 0.496475 0.603456

H 6.202086 1.053432 -0.435548

H 6.576198 0.643703 -2.868354

H 4.748945 -0.352654 -4.247782

H 2.585388 -0.956832 -3.193676

(*M*,S_P_,*M*)-**2**

E(hartree): -1573.530905

C 1.333844 1.814708 0.169068

C 0.286749 0.794400 0.288961

C 0.671980 -0.576840 0.686225

C 2.047163 -1.039508 0.490784

C 2.901916 -0.533579 -0.533777

C 4.193300 -0.991441 -0.692573

C 4.723314 -1.974059 0.178243

C 3.904039 -2.549500 1.123338

C 2.543518 -2.149185 1.256217

C 1.165036 2.934001 -0.708335

C -0.099126 3.116361 -1.328998

C -1.162600 2.381091 -0.874683

C -1.026632 1.282623 0.034305

C 2.218305 3.874142 -0.873811

C 3.364797 3.794315 -0.112455

C 3.478881 2.775213 0.861790

C 2.499243 1.810877 0.989791

C 1.638251 -2.899819 2.049586

C 0.288680 -2.679313 1.894218

C -0.216797 -1.572202 1.160362

C -2.280717 0.962409 0.782051

C -2.993772 2.085802 1.263175

C -4.174333 1.952751 1.994417

C -4.686682 0.684741 2.275371

C -3.989856 -0.437513 1.826319

C -2.800752 -0.310394 1.091625

P -1.954791 -1.851932 0.584817

C -1.742480 -1.569424 -1.246524

C -2.721987 -0.896500 -1.994891

C -2.595355 -0.767519 -3.381160

C -1.489431 -1.309674 -4.041282

C -0.515432 -1.993391 -3.305702

C -0.643953 -2.129895 -1.921950

H -4.369231 -1.439395 2.041300

H -5.613218 0.569914 2.841967

H -4.687143 2.848113 2.353378

H -2.592685 3.085202 1.087636

H -2.169405 2.614156 -1.225039

H -0.235408 3.903659 -2.073723

H 2.076988 4.692541 -1.584426

H 4.159715 4.534187 -0.228882

H 4.346646 2.749584 1.524365

H 2.611098 1.042357 1.752450

H 2.517802 0.210729 -1.227646

H 4.807424 -0.594181 -1.503570

H 5.758502 -2.304409 0.067677

H 4.268186 -3.364298 1.754273

H 2.009021 -3.724562 2.662607

H -0.432939 -3.380502 2.320552

H -3.582917 -0.451532 -1.490851

H -3.362211 -0.230961 -3.945436

H -1.386408 -1.201310 -5.123425

H 0.353235 -2.422404 -3.811135

H 0.130144 -2.659958 -1.362569

(*P*,S_P_,*M*)-**2**

E(hartree): -1573.585692

C -0.097893 1.760729 0.654638

C 0.108730 0.336050 0.596065

C 1.183442 -0.197630 -0.296125

C 2.559093 0.116943 -0.010599

C 2.956755 0.812727 1.169678

C 4.282185 1.098179 1.417780

C 5.288377 0.707371 0.498344

C 4.943449 0.019377 -0.643840

C 3.585055 -0.302540 -0.923621

C -1.081840 2.300908 1.545577

C -1.829280 1.412578 2.363296

C -1.656254 0.056299 2.254009

C -0.711322 -0.507807 1.345733

C -1.312121 3.703726 1.572201

C -0.615608 4.552466 0.739525

C 0.344307 4.023306 -0.157749

C 0.598262 2.668185 -0.195623

C 3.223124 -1.042860 -2.079830

C 1.910767 -1.391634 -2.300301

C 0.879613 -0.983193 -1.409246

C -0.730534 -1.988884 1.200556

C -0.691694 -2.793233 2.352864

C -0.811490 -4.182315 2.277035

C -0.989815 -4.797900 1.036756

C -1.026729 -4.014031 -0.119478

C -0.894016 -2.617441 -0.061549

P -0.795907 -1.740977 -1.704118

C -1.964243 -0.307146 -1.548001

C -3.187556 -0.432091 -0.870737

C -4.094776 0.630069 -0.844607

C -3.797546 1.829087 -1.499471

C -2.588390 1.955620 -2.189696

C -1.682472 0.893736 -2.220448

H -1.154188 -4.490950 -1.094357

H -1.094027 -5.882916 0.966340

H -0.765048 -4.781571 3.189261

H -0.550846 -2.315361 3.325131

H -2.283118 -0.620193 2.837637

H -2.574508 1.821863 3.049796

H -2.065287 4.095500 2.260706

H -0.805836 5.627793 0.763106

H 0.885249 4.693517 -0.829627

H 1.332256 2.280185 -0.900588

H 2.195271 1.115367 1.887692

H 4.560339 1.627521 2.331847

H 6.334652 0.944870 0.703447

H 5.711598 -0.299859 -1.352941

H 4.004485 -1.350703 -2.779399

H 1.641765 -1.995585 -3.169933

H -3.421933 -1.356428 -0.337055

H -5.035093 0.522736 -0.298216

H -4.502797 2.662585 -1.468506

H -2.339790 2.891760 -2.694789

H -0.732023 1.012642 -2.746590

Transition state of **2** (backbone 2)

E(hartree):-1573.512529

C -1.779757 0.617469 -2.012528

C -1.945802 -0.695194 -1.545187

C -3.240269 -1.147156 -1.235898

C -4.340418 -0.297732 -1.367525

C -4.163799 1.016249 -1.812679

C -2.880671 1.468841 -2.134792

P -0.562102 -1.922727 -1.524975

C 1.036607 -1.061699 -1.074539

C 1.288092 0.094390 -0.304379

C 2.698222 0.530457 -0.323803

C 3.728962 -0.453949 -0.539840

C 3.386703 -1.718307 -1.086370

C 2.097110 -1.930470 -1.483719

C 3.174599 1.870392 -0.283407

C 4.516275 2.193454 -0.184923

C 5.498129 1.182103 -0.142460

C 5.099996 -0.118129 -0.363272

C 0.144666 0.617254 0.562980

C -0.694042 -0.350916 1.157187

C -1.981697 -0.009536 1.675379

C -2.501453 1.244009 1.533923

C -1.651224 2.293360 1.109050

C -0.277245 2.009603 0.780509

C -2.135672 3.630459 1.123878

C -1.288090 4.704072 0.966212

C 0.095771 4.448177 0.869853

C 0.569673 3.151549 0.791415

C -0.475113 -1.831215 1.271649

C -0.293239 -2.398976 2.540359

C -0.269952 -3.788616 2.703868

C -0.457439 -4.629063 1.603985

C -0.638323 -4.073766 0.332615

C -0.647269 -2.684290 0.162122

H -0.756299 -4.722084 -0.539159

H -0.451612 -5.713897 1.731993

H -0.108963 -4.213363 3.697454

H -0.168333 -1.743066 3.405006

H -2.593702 -0.813982 2.084189

H -3.538738 1.459387 1.797814

H -3.200982 3.785041 1.313039

H -1.667615 5.727703 0.989955

H 0.808907 5.275665 0.890772

H 1.635651 3.005790 0.849920

H 2.477951 2.676870 -0.457846

H 4.812451 3.245042 -0.179544

H 6.554204 1.432826 -0.023644

H 5.840379 -0.914944 -0.471694

H 4.170428 -2.453951 -1.281527

H 1.838380 -2.828803 -2.048903

H -3.387096 -2.167924 -0.872216

H -5.339067 -0.661860 -1.113776

H -5.022120 1.685267 -1.907837

H -2.730732 2.496974 -2.472190

H -0.781530 0.987269 -2.255169

(R_P_,*M*)-**3**

E(hartree): -1647.645087

C 4.496835 -1.049211 -0.306683

C 3.207845 -0.521162 -0.249078

C 2.113615 -1.391077 0.053531

C 2.390810 -2.738133 0.461266

C 3.721042 -3.228092 0.394569

C 4.749906 -2.402279 -0.010678

C 0.756514 -0.914608 0.009244

C 0.490511 0.389149 -0.638065

C 1.583311 1.331302 -0.674427

C 2.929879 0.911681 -0.422330

C 1.349027 2.704476 -1.008383

C 0.050546 3.090007 -1.431660

C -0.923844 2.141438 -1.597695

C -0.733500 0.775655 -1.229235

C 3.942847 1.869885 -0.373982

C 3.684759 3.231622 -0.613485

C 2.409890 3.642543 -0.946064

C 1.332507 -3.540998 0.964881

C 0.074546 -3.008314 1.110554

C -0.220382 -1.701978 0.641298

C -1.859498 -0.123396 -1.623356

C -2.544978 -0.978004 -0.741024

C -3.630340 -1.754123 -1.165966

C -4.061416 -1.683437 -2.490628

C -3.392606 -0.840735 -3.385729

C -2.311758 -0.068382 -2.957387

P -1.947522 -1.188155 0.958516

O -2.740685 -2.218953 1.747939

C -1.884578 0.489428 1.697649

C -0.802412 0.900070 2.489156

C -0.804864 2.167842 3.075513

C -1.889525 3.028745 2.881105

C -2.975897 2.619086 2.100928

C -2.974036 1.353840 1.510495

H 0.052438 0.236402 2.633419

H 0.046003 2.485975 3.682060

H -1.887680 4.021504 3.337149

H -3.824668 3.289449 1.947340

H -3.817512 1.040474 0.890540

H -4.115507 -2.406833 -0.436855

H -4.909917 -2.283530 -2.826412

H -3.710463 -0.787483 -4.429602

H -1.791015 0.574512 -3.670147

H -1.901635 2.432753 -1.983884

H -0.147991 4.138927 -1.663103

H 2.200697 4.689644 -1.177051

H 4.501829 3.953669 -0.555179

H 4.963164 1.566272 -0.139091

H 5.335266 -0.409203 -0.582452

H 5.770824 -2.784497 -0.075526

H 3.914886 -4.264634 0.679810

H 1.545452 -4.564508 1.282435

H -0.734383 -3.571988 1.579286

(R_P_,*P*)-**3**

E(hartree): -1647.633095

C -3.961916 -2.607245 -0.757946

C -3.121007 -1.583705 -0.322918

C -1.706849 -1.723492 -0.494995

C -1.192621 -2.984216 -0.943062

C -2.086547 -4.002391 -1.364432

C -3.449921 -3.801823 -1.298802

C -0.808906 -0.643138 -0.177863

C -1.392771 0.697726 0.045996

C -2.768595 0.753687 0.477027

C -3.625947 -0.386835 0.361813

C -3.325117 1.973062 0.982392

C -2.540232 3.153307 0.928094

C -1.312668 3.128667 0.320417

C -0.717911 1.920702 -0.151452

C -4.922192 -0.320823 0.873260

C -5.427249 0.855375 1.455862

C -4.648065 1.993838 1.491313

C 0.207754 -3.202200 -0.906281

C 1.044697 -2.232018 -0.410999

C 0.563665 -0.943408 -0.054003

C 0.542311 2.132743 -0.923652

C 1.778749 1.523270 -0.622660

C 2.938854 1.897534 -1.318275

C 2.881259 2.841138 -2.343985

C 1.653992 3.419831 -2.679985

C 0.504970 3.078902 -1.967661

P 1.721322 0.272649 0.706186

O 1.279258 0.795255 2.062449

C 3.385105 -0.504091 0.796764

C 4.112089 -1.001816 -0.299485

C 5.380138 -1.554608 -0.107116

C 5.930947 -1.614598 1.178540

C 5.212569 -1.119668 2.270414

C 3.943509 -0.564165 2.081645

H 3.689319 -0.960804 -1.305758

H 5.940110 -1.939604 -0.962478

H 6.922978 -2.047847 1.326446

H 5.641294 -1.164497 3.274165

H 3.365421 -0.165477 2.917874

H 3.899230 1.458551 -1.047948

H 3.791354 3.124083 -2.877337

H 1.591143 4.145674 -3.493836

H -0.449030 3.542375 -2.226843

H -0.738206 4.050984 0.224498

H -2.941521 4.083761 1.336037

H -5.039062 2.928180 1.900802

H -6.442448 0.865779 1.857809

H -5.565739 -1.199778 0.831283

H -5.043025 -2.491759 -0.678396

H -4.139616 -4.578149 -1.636844

H -1.675389 -4.946581 -1.728937

H 0.605554 -4.170191 -1.220068

H 2.101166 -2.463737 -0.290659

Transition state of **3**

E(hartree): -1647.593189

C -3.994431 -2.762779 0.041783

C -3.147838 -1.653896 0.099345

C -1.757882 -1.795164 -0.194105

C -1.286193 -3.115465 -0.513872

C -2.183050 -4.212120 -0.578730

C -3.520558 -4.039461 -0.298226

C -0.844597 -0.666378 -0.127591

C -1.420174 0.713133 -0.167520

C -2.868069 0.808625 0.003840

C -3.669568 -0.318755 0.370345

C -3.576711 2.042634 -0.168307

C -2.906025 3.075579 -0.864565

C -1.561733 2.978711 -1.053170

C -0.739084 1.940055 -0.485322

C -4.964792 -0.125277 0.858443

C -5.583538 1.132982 0.835380

C -4.916877 2.193733 0.255346

C 0.094077 -3.324572 -0.734232

C 0.970362 -2.301284 -0.495507

C 0.530597 -0.992650 -0.153170

C 0.669479 2.417835 -0.262320

C 1.823632 1.668301 0.083950

C 3.083498 2.290782 0.216524

C 3.257771 3.656547 0.049209

C 2.122738 4.433412 -0.186962

C 0.879973 3.829811 -0.318042

P 1.790361 -0.021361 0.724838

O 1.600392 -0.087675 2.233322

C 3.417483 -0.730480 0.232239

C 3.913001 -0.688291 -1.081007

C 5.158978 -1.244149 -1.374859

C 5.916590 -1.841183 -0.358493

C 5.427047 -1.879522 0.949969

C 4.177544 -1.324023 1.247085

H 3.323732 -0.218610 -1.872728

H 5.543399 -1.211018 -2.396986

H 6.892412 -2.274652 -0.590266

H 6.019422 -2.341650 1.743128

H 3.773083 -1.335585 2.261633

H 3.945232 1.679558 0.484841

H 4.245651 4.108918 0.154369

H 2.194116 5.521795 -0.245454

H 0.032732 4.499882 -0.419740

H -1.076942 3.755847 -1.636285

H -3.466439 3.932839 -1.244028

H -5.401571 3.163744 0.124160

H -6.598280 1.250357 1.220666

H -5.526073 -0.977672 1.240513

H -5.060230 -2.638210 0.228535

H -4.214200 -4.881391 -0.345385

H -1.786249 -5.194858 -0.842933

H 0.453320 -4.315779 -1.019070

H 2.038418 -2.501834 -0.548849

(R_P_,*M*)-**4**

E(hartree): -1572.393308

P -1.905965 -1.364625 1.350327

C -2.582510 -1.342890 -0.365522

C -0.154387 -1.755450 0.881753

C -1.866577 0.442302 1.783488

C -0.783596 1.007297 2.475705

C -0.815288 2.346945 2.870313

C -1.934406 3.138495 2.593574

C -3.025129 2.578108 1.922076

C -2.992729 1.240109 1.521999

C -3.619433 -2.233863 -0.684139

C -4.115192 -2.325203 -1.985686

C -3.563009 -1.519646 -2.985256

C -2.537624 -0.625464 -2.675373

C -2.024447 -0.518439 -1.366473

C 0.296254 -3.006869 1.386725

C 1.580869 -3.450298 1.184463

C 2.536015 -2.593874 0.576750

C 2.116044 -1.301831 0.118106

C 0.720755 -0.943890 0.134156

C 3.111683 -0.363905 -0.302924

C 2.700547 1.024550 -0.553224

C 1.311583 1.304931 -0.759850

C 0.312317 0.279672 -0.586548

C 0.937547 2.620285 -1.185639

C -0.408769 2.853457 -1.569241

C -1.299372 1.812882 -1.603838

C -0.968314 0.507559 -1.131645

C 3.624157 2.067605 -0.628445

C 3.231704 3.378715 -0.953367

C 1.910780 3.648747 -1.249910

C 3.897927 -2.973674 0.456400

C 4.826264 -2.088870 -0.053385

C 4.437278 -0.783298 -0.408540

H 0.103706 0.405545 2.684622

H 0.044330 2.775941 3.390862

H -1.955627 4.187000 2.899296

H -3.904614 3.187603 1.699891

H -3.844118 0.818812 0.981101

H -4.037919 -2.861498 0.106382

H -4.924342 -3.021038 -2.218369

H -3.927742 -1.588429 -4.012710

H -2.104141 -0.009428 -3.466156

H -2.317921 1.984446 -1.954875

H -0.713309 3.857979 -1.871614

H 1.597192 4.652008 -1.547990

H 3.981008 4.172202 -0.991228

H 4.677863 1.872845 -0.427383

H 5.198158 -0.092238 -0.772160

H 5.871943 -2.385749 -0.158744

H 4.197176 -3.971930 0.784304

H 1.895460 -4.434483 1.539712

H -0.413279 -3.625385 1.941141

(S_P_,*M*)-**4**

E(hartree): -1572.383674

P -1.728049 0.335112 0.787503

C -1.807880 1.480665 -0.663333

C -0.645141 -0.869194 -0.122369

C -3.386074 -0.467226 0.961116

C -3.493677 -1.794850 1.421170

C -4.736555 -2.344178 1.744966

C -5.899087 -1.574988 1.639065

C -5.802970 -0.243274 1.226489

C -4.560745 0.307950 0.900737

C -2.857071 1.668612 -1.573943

C -2.725683 2.576606 -2.626931

C -1.535745 3.292876 -2.786480

C -0.475849 3.082852 -1.903523

C -0.587774 2.168953 -0.839164

C -1.134045 -2.054134 -0.738469

C -0.278159 -2.977716 -1.289503

C 1.129799 -2.819789 -1.167047

C 1.636534 -1.627188 -0.557421

C 0.732077 -0.581097 -0.169487

C 3.037134 -1.519698 -0.285420

C 3.513761 -0.372140 0.502869

C 2.653812 0.763520 0.661717

C 1.295553 0.733226 0.184401

C 3.183947 1.954975 1.253546

C 2.393246 3.136450 1.233633

C 1.185188 3.145536 0.584738

C 0.611211 1.961890 0.031499

C 4.791906 -0.331915 1.061732

C 5.270373 0.810251 1.729018

C 4.488990 1.946631 1.805836

C 2.035013 -3.816491 -1.609632

C 3.393822 -3.659765 -1.414419

C 3.888060 -2.528254 -0.739032

H -2.597653 -2.407710 1.538408

H -4.793406 -3.379601 2.089428

H -6.870507 -2.005704 1.891592

H -6.700014 0.377339 1.162128

H -4.509322 1.359532 0.612845

H -3.780242 1.098365 -1.468693

H -3.553106 2.720103 -3.325840

H -1.424997 4.002331 -3.609481

H 0.468155 3.612016 -2.051137

H 0.617838 4.074349 0.501244

H 2.782774 4.045680 1.697627

H 4.867678 2.855462 2.279460

H 6.272431 0.799151 2.162998

H 5.444013 -1.202369 0.986520

H 4.962877 -2.448354 -0.574311

H 4.092010 -4.423290 -1.764011

H 1.639754 -4.711148 -2.096629

H -0.671604 -3.865130 -1.791593

H -2.209760 -2.222272 -0.791249

Transition state of **4** (P-inversion)

E(hartree): -1572.338811

C -3.016132 -2.227974 1.435510

C -3.231085 -0.997639 0.777160

C -4.452797 -0.318495 0.977684

C -5.427443 -0.863602 1.812442

C -5.217761 -2.088745 2.456164

C -4.006137 -2.761072 2.260733

P -1.984965 -0.309460 -0.299370

C -0.445293 -1.157699 -0.624672

C 0.832690 -0.580952 -0.446021

C 1.978566 -1.416336 -0.710356

C 1.824536 -2.693058 -1.345382

C 0.513772 -3.205917 -1.547965

C -0.578004 -2.477794 -1.159104

C 3.288688 -1.026274 -0.288569

C 3.412110 0.105182 0.638302

C 2.304141 0.999682 0.795976

C 1.059506 0.777114 0.094825

C 2.476732 2.152213 1.631761

C 1.437674 3.115797 1.689243

C 0.351357 2.992382 0.863347

C 0.151599 1.859718 0.017276

C 4.578037 0.325717 1.373439

C 4.709858 1.423268 2.241698

C 3.678664 2.331923 2.361573

C 2.967794 -3.439134 -1.721468

C 4.234864 -2.968669 -1.433923

C 4.389146 -1.783527 -0.694943

C -0.939069 2.084741 -0.991587

C -2.095150 1.287396 -1.086943

C -3.170027 1.674680 -1.908611

C -3.097416 2.856380 -2.644537

C -1.927497 3.624190 -2.611115

C -0.861514 3.236940 -1.796707

H -2.070662 -2.759058 1.309888

H -3.821763 -3.713573 2.763915

H -5.986961 -2.511518 3.105215

H -6.364613 -0.321133 1.960375

H -4.633231 0.641002 0.488423

H -4.050833 1.033732 -1.981065

H -3.939936 3.161081 -3.269371

H -1.847907 4.531570 -3.213647

H 0.040594 3.851098 -1.754857

H -0.404512 3.779276 0.842166

H 1.540919 3.978260 2.351693

H 3.773901 3.206312 3.009594

H 5.633863 1.555099 2.808532

H 5.408843 -0.375002 1.292112

H 5.395985 -1.462394 -0.427512

H 5.117993 -3.531661 -1.742920

H 2.827941 -4.393758 -2.234031

H 0.390257 -4.188999 -2.008264

H -1.581877 -2.881049 -1.301066

(R_P_,*P*)-**4**

E(hartree): -1572.387591

C 3.853786 -2.615954 0.803718

C 3.041193 -1.584894 0.333071

C 3.584885 -0.404126 -0.351924

C 4.899322 -0.357799 -0.818081

C 5.434122 0.803506 -1.404112

C 4.666962 1.947713 -1.492273

C 3.326397 1.947982 -1.031444

C 2.744471 0.742783 -0.520046

C 2.548061 3.135652 -1.028730

C 1.301572 3.133198 -0.459360

C 0.685438 1.939303 0.025138

C 1.355821 0.708203 -0.129040

C 1.620275 -1.704880 0.460742

C 0.745965 -0.617601 0.105347

C 3.309174 -3.799748 1.336478

C 1.941470 -3.982872 1.353922

C 1.076397 -2.956509 0.894994

C -0.324299 -3.154069 0.800595

C -1.131692 -2.174712 0.274086

C -0.627908 -0.889207 -0.067153

C -0.576612 2.169861 0.793127

C -1.814304 1.580379 0.464697

C -2.977229 1.988931 1.134939

C -2.915847 2.932496 2.163286

C -1.684322 3.476033 2.535347

C -0.530082 3.104373 1.845009

P -1.722117 0.359417 -0.924695

C -3.405015 -0.430096 -0.918859

C -4.026321 -0.566753 -2.171109

C -5.300682 -1.133839 -2.284390

C -5.974045 -1.571693 -1.142246

C -5.368772 -1.442502 0.113749

C -4.096877 -0.877973 0.224722

H -3.633129 -0.787837 1.208937

H -5.890107 -1.784727 1.011030

H -6.969475 -2.013917 -1.227253

H -5.766112 -1.231798 -3.267880

H -3.503883 -0.223049 -3.067461

H -3.945143 1.580460 0.844966

H -3.832461 3.240124 2.672011

H -1.621514 4.198922 3.351706

H 0.431893 3.541562 2.121169

H 0.734787 4.063013 -0.387771

H 2.972625 4.054905 -1.438942

H 5.081894 2.870234 -1.905171

H 6.463802 0.798411 -1.767876

H 5.533899 -1.240487 -0.737076

H 4.938454 -2.515297 0.758104

H 3.976921 -4.581716 1.704218

H 1.505597 -4.919725 1.709012

H -0.748359 -4.115122 1.102255

H -2.185764 -2.396326 0.121236

Transition state of **4** (backbone)

E(hartree): -1572.341796

C -4.325286 -1.416444 -1.203994

C -3.452542 -0.765646 -0.320719

C -3.811428 -0.654949 1.035367

C -5.015077 -1.189993 1.493758

C -5.879893 -1.841206 0.603589

C -5.534867 -1.953267 -0.744554

P -1.865247 -0.070271 -1.010424

C -1.919810 1.625080 -0.313246

C -0.787972 2.386757 0.067283

C -0.994527 3.797569 0.091387

C -2.232439 4.405322 -0.081924

C -3.356387 3.618167 -0.325770

C -3.176365 2.247760 -0.469979

C 0.614700 1.917238 0.376298

C 1.337180 0.714266 0.072955

C 2.790213 0.841171 0.011893

C 3.461806 2.080145 0.280669

C 2.723377 3.076164 0.957947

C 1.368492 2.949649 1.036789

C 3.638980 -0.257785 -0.338895

C 3.127303 -1.612294 -0.148984

C 1.723805 -1.791181 0.050031

C 0.788745 -0.681024 -0.039198

C 1.269117 -3.133212 0.295458

C 2.184703 -4.214235 0.375570

C 3.532051 -4.004607 0.186748

C 3.993661 -2.704239 -0.075384

C -0.578536 -1.029984 -0.100750

C -0.999959 -2.358788 0.187137

C -0.114942 -3.374543 0.430494

C 4.826815 2.269589 -0.042796

C 5.550979 1.244987 -0.615450

C 4.958739 -0.022906 -0.730169

H -3.140261 -0.144924 1.729991

H -5.283166 -1.099954 2.549284

H -6.822932 -2.258489 0.964495

H -6.206435 -2.457560 -1.443387

H -4.055095 -1.503976 -2.259417

H -4.036552 1.635832 -0.744986

H -4.345692 4.062206 -0.454723

H -2.305934 5.494236 -0.043501

H -0.144590 4.461615 0.217438

H 0.820842 3.701991 1.597597

H 3.235869 3.931677 1.403420

H 5.281765 3.242234 0.157750

H 6.587495 1.393820 -0.924759

H 5.562708 -0.849994 -1.102820

H 5.065907 -2.549007 -0.187964

H 4.241893 -4.832004 0.247623

H 1.793474 -5.213374 0.580767

H -0.470100 -4.380365 0.666201

H -2.064293 -2.585279 0.194001

**5**

E(hartree): -999.390523

C -0.005885 -0.006115 -0.013897

C -0.010026 -0.014876 1.397865

C 1.226425 -0.037676 2.118337

C 2.457836 -0.051473 1.389971

C 2.408683 -0.041833 -0.009260

C 1.192853 -0.019438 -0.706211

C 1.210377 -0.046203 3.557031

C 2.459163 -0.069243 4.284257

C 3.702450 -0.083458 3.560230

C 3.728136 -0.075169 2.129719

C 4.939277 -0.106411 4.280048

C 6.165021 -0.120655 3.579675

C 6.175480 -0.112533 2.195381

C 4.969281 -0.090063 1.481888

C 2.460123 -0.077849 5.698905

C 3.717441 -0.100677 6.383039

C 4.903906 -0.114405 5.706735

C -0.020396 -0.032083 4.254401

C -0.031622 -0.040534 5.707047

C 1.202338 -0.063504 6.425634

C -1.233337 -0.001350 2.132768

C -1.235932 -0.009616 3.498490

C 1.157156 -0.071553 7.845666

C -0.038550 -0.057716 8.537171

C -1.255527 -0.034816 7.828478

C -1.244293 -0.026547 6.447213

H 2.083254 -0.089089 8.417763

H -0.037043 -0.064574 9.629157

H -2.205945 -0.023566 8.366123

H -2.198906 -0.008635 5.924099

H -2.193933 0.001237 4.015054

H -2.176206 0.015866 1.580974

H -0.959303 0.011307 -0.547111

H 1.199100 -0.012597 -1.798216

H 3.331467 -0.051735 -0.587970

H 5.017139 -0.084368 0.393673

H 7.122093 -0.123559 1.650991

H 7.099304 -0.138133 4.145747

H 5.849139 -0.131861 6.254463

H 3.741021 -0.107351 7.471216

**Table S1:** Vertical excitation energies (in eV), wavelengths (in nm) and oscillator strengths (f) of (R_P_,*M*)-**1** at the TD-B3LYP/cc-pVDZ level

Excited State 1: Singlet-A 3.7386 eV 331.63 nm f=0.0476 <S**2>=0.000

117 ->119 -0.28636

118 ->119 0.60575

118 ->120 0.17853

This state for optimization and/or second-order correction.

Total Energy, E(TD-HF/TD-DFT) = -1648.62675962

Copying the excited state density for this state as the 1-particle RhoCI density.

Excited State 2: Singlet-A 3.7641 eV 329.39 nm f=0.0290 <S**2>=0.000

117 ->119 -0.32624

117 ->120 0.16964

118 ->119 -0.28141

118 ->120 0.52125

Excited State 3: Singlet-A 3.9731 eV 312.06 nm f=0.0386 <S**2>=0.000

117 ->119 0.38118

117 ->120 0.50027

118 ->119 0.19311

118 ->120 0.20064

Excited State 4: Singlet-A 4.0725 eV 304.44 nm f=0.0187 <S**2>=0.000

117 ->119 -0.35062

117 ->120 0.43890

117 ->121 0.10588

118 ->120 -0.35889

Excited State 5: Singlet-A 4.0946 eV 302.80 nm f=0.0014 <S**2>=0.000

116 ->119 0.46667

116 ->120 0.11154

117 ->119 -0.15537

117 ->121 -0.18619

118 ->121 -0.41044

Excited State 6: Singlet-A 4.2619 eV 290.91 nm f=0.0018 <S**2>=0.000

114 ->119 -0.22773

114 ->120 0.25371

115 ->119 -0.12602

116 ->120 0.30720

117 ->121 -0.27881

117 ->122 0.10517

118 ->120 -0.10249

118 ->121 0.22518

118 ->122 -0.23165

118 ->123 0.10973

118 ->124 0.10770

Excited State 7: Singlet-A 4.4102 eV 281.13 nm f=0.0255 <S**2>=0.000

114 ->120 0.13899

115 ->119 0.42654

116 ->119 0.34887

116 ->120 -0.25350

118 ->121 0.25426

Excited State 8: Singlet-A 4.4353 eV 279.54 nm f=0.0153 <S**2>=0.000

115 ->119 0.50454

116 ->119 -0.13507

116 ->120 0.41880

118 ->121 -0.13040

Excited State 9: Singlet-A 4.4969 eV 275.71 nm f=0.0441 <S**2>=0.000

114 ->119 0.20399

114 ->120 -0.14732

115 ->119 -0.11521

116 ->119 0.10591

116 ->120 0.21715

117 ->121 -0.24130

117 ->122 -0.22625

118 ->121 0.33416

118 ->122 0.28900

118 ->124 -0.12356

Excited State 10: Singlet-A 4.5468 eV 272.69 nm f=0.0052 <S**2>=0.000

113 ->119 -0.12233

114 ->119 0.23943

115 ->120 0.61350

Excited State 11: Singlet-A 4.5590 eV 271.96 nm f=0.0505 <S**2>=0.000

113 ->119 -0.12725

114 ->119 0.14499

114 ->120 0.21489

115 ->120 -0.11422

116 ->119 -0.12265

116 ->120 -0.12689

117 ->121 -0.32211

117 ->122 0.24135

118 ->121 -0.13552

118 ->122 0.37354

Excited State 12: Singlet-A 4.5814 eV 270.62 nm f=0.0030 <S**2>=0.000

112 ->119 0.10156

112 ->120 -0.13506

113 ->119 0.58041

113 ->120 -0.14302

116 ->120 0.17772

117 ->122 0.14758

118 ->122 0.16005

Excited State 13: Singlet-A 4.6083 eV 269.04 nm f=0.0553 <S**2>=0.000

113 ->120 0.28186

114 ->119 0.34141

114 ->120 0.30363

115 ->120 -0.23040

116 ->119 0.16282

116 ->120 0.11386

117 ->121 0.26577

Excited State 14: Singlet-A 4.6219 eV 268.25 nm f=0.0187 <S**2>=0.000

112 ->119 0.16043

112 ->120 -0.11951

113 ->120 -0.29564

114 ->119 0.37243

116 ->119 -0.12136

117 ->121 -0.16556

117 ->122 -0.14698

118 ->122 -0.33314

118 ->123 -0.13133

Excited State 15: Singlet-A 4.7086 eV 263.32 nm f=0.0859 <S**2>=0.000

112 ->119 -0.28243

113 ->119 0.26750

113 ->120 0.35228

116 ->120 -0.11233

117 ->121 -0.23483

118 ->122 -0.14424

118 ->123 -0.25448

Excited State 16: Singlet-A 4.7339 eV 261.91 nm f=0.0025 <S**2>=0.000

112 ->119 0.58516

112 ->120 0.18500

113 ->120 0.23912

114 ->120 -0.15441

118 ->123 -0.10274

Excited State 17: Singlet-A 4.7882 eV 258.94 nm f=0.0257 <S**2>=0.000

111 ->119 0.58222

111 ->120 0.14412

113 ->119 0.10043

118 ->123 0.21353

118 ->124 -0.10782Excited State 18: Singlet-A 4.8390 eV 256.22 nm f=0.0615 <S**2>=0.000

110 ->119 0.13532

111 ->119 -0.18288

117 ->122 -0.35403

118 ->123 0.39644

118 ->124 0.29543Excited State 19: Singlet-A 4.8452 eV 255.89 nm f=0.0590 <S**2>=0.000

110 ->119 -0.10234

111 ->119 0.18616

112 ->120 0.13330

114 ->120 0.17342

117 ->122 -0.26597

117 ->123 -0.14737

118 ->122 0.10668

118 ->123 -0.32696

118 ->124 0.37400Excited State 20: Singlet-A 4.8955 eV 253.26 nm f=0.0021 <S**2>=0.000

109 ->119 0.12068

111 ->119 -0.16717

112 ->120 0.57502

113 ->120 -0.20431

114 ->120 0.14194

118 ->124 -0.11025

**Table S2:** Vertical excitation energies (in eV), wavelengths (in nm) and oscillator strengths (f) of (R_P_,*P*)-**1** at the TD-B3LYP/cc-pVDZ level

Excited State 1: Singlet-A 3.4952 eV 354.73 nm f=0.0267 <S**2>=0.000

117 ->119 -0.12450

118 ->119 0.68044

118 ->120 -0.12365

This state for optimization and/or second-order correction.

Total Energy, E(TD-HF/TD-DFT) = -1648.62941671

Copying the excited state density for this state as the 1-particle RhoCI density.

Excited State 2: Singlet-A 3.8263 eV 324.03 nm f=0.0724 <S**2>=0.000

117 ->119 0.31492

117 ->120 0.13026

118 ->119 0.16427

118 ->120 0.56022

118 ->121 -0.10797

Excited State 3: Singlet-A 3.9781 eV 311.67 nm f=0.0125 <S**2>=0.000

117 ->119 -0.45386

117 ->120 0.48007

118 ->120 0.12527

118 ->121 -0.10064

Excited State 4: Singlet-A 4.0343 eV 307.33 nm f=0.0367 <S**2>=0.000

117 ->119 0.38326

117 ->120 0.47594

118 ->120 -0.31453

Excited State 5: Singlet-A 4.1165 eV 301.19 nm f=0.0135 <S**2>=0.000

115 ->119 0.10377

116 ->119 0.27599

116 ->120 0.24316

117 ->120 0.11088

118 ->120 0.18065

118 ->121 0.48896

118 ->122 0.14507

118 ->123 -0.10767

Excited State 6: Singlet-A 4.1821 eV 296.46 nm f=0.0022 <S**2>=0.000

113 ->119 -0.12284

114 ->119 0.12031

115 ->119 0.41594

116 ->119 0.11334

116 ->120 -0.28176

117 ->121 0.31765

117 ->122 0.11401

117 ->125 0.10387

118 ->123 0.20683

Excited State 7: Singlet-A 4.2297 eV 293.13 nm f=0.0043 <S**2>=0.000

115 ->119 -0.25007

116 ->119 0.58745

116 ->120 -0.12307

118 ->121 -0.22908

Excited State 8: Singlet-A 4.3115 eV 287.57 nm f=0.0333 <S**2>=0.000

113 ->119 0.14848

115 ->119 0.34948

115 ->120 -0.12260

116 ->120 0.27868

118 ->121 -0.30576

118 ->122 -0.13405

118 ->123 -0.31113

Excited State 9: Singlet-A 4.3804 eV 283.05 nm f=0.0026 <S**2>=0.000

118 ->121 -0.21206

118 ->122 0.65821

Excited State 10: Singlet-A 4.4053 eV 281.44 nm f=0.0372 <S**2>=0.000

113 ->119 -0.31386

114 ->119 0.22726

115 ->119 -0.26175

115 ->120 -0.10201

116 ->119 -0.15636

116 ->120 0.16675

117 ->121 0.33045

117 ->122 0.11055

117 ->123 0.10776

118 ->121 -0.12657

118 ->123 -0.14388

Excited State 11: Singlet-A 4.4750 eV 277.06 nm f=0.0080 <S**2>=0.000

113 ->119 0.27472

114 ->119 0.61319

117 ->121 -0.10299

Excited State 12: Singlet-A 4.5146 eV 274.63 nm f=0.0095 <S**2>=0.000

113 ->120 0.11027

115 ->120 0.64999

116 ->120 0.12487

Excited State 13: Singlet-A 4.6015 eV 269.44 nm f=0.0075 <S**2>=0.000

113 ->119 -0.11763

117 ->121 -0.36705

117 ->122 0.52989

117 ->123 0.12687

Excited State 14: Singlet-A 4.6769 eV 265.10 nm f=0.0789 <S**2>=0.000

114 ->120 0.37922

116 ->120 0.21040

117 ->122 -0.17740

117 ->123 0.19993

118 ->123 0.35923

118 ->124 -0.11631

118 ->125 0.21120

Excited State 15: Singlet-A 4.7017 eV 263.70 nm f=0.0845 <S**2>=0.000

113 ->120 0.11392

114 ->120 0.53864

116 ->120 -0.14159

117 ->122 0.17557

117 ->123 -0.20172

118 ->123 -0.23961

Excited State 16: Singlet-A 4.7269 eV 262.29 nm f=0.1448 <S**2>=0.000

113 ->119 0.23337

116 ->120 0.20525

116 ->121 -0.13539

117 ->121 0.23359

117 ->122 0.29156

117 ->123 -0.11648

117 ->125 -0.12891

118 ->123 0.26884

118 ->124 0.24306

118 ->125 -0.19841

Excited State 17: Singlet-A 4.7752 eV 259.64 nm f=0.0236 <S**2>=0.000

113 ->119 -0.17985

114 ->120 0.16452

116 ->121 0.19765

117 ->121 -0.11601

117 ->123 0.13516

117 ->125 0.11639

118 ->124 0.48085

118 ->125 -0.23274

Excited State 18: Singlet-A 4.8462 eV 255.84 nm f=0.0414 <S**2>=0.000

109 ->120 0.12001

111 ->119 -0.12432

112 ->119 0.12429

113 ->120 0.41882

116 ->121 -0.24665

116 ->122 -0.12985

117 ->123 0.29573

118 ->124 0.13914

118 ->125 -0.11039

Excited State 19: Singlet-A 4.8731 eV 254.43 nm f=0.0071 <S**2>=0.000

113 ->120 0.21342

115 ->121 -0.12327

116 ->120 -0.10803

116 ->121 0.22393

117 ->123 -0.16598

117 ->124 0.10671

118 ->124 0.25625

118 ->125 0.45881

118 ->126 -0.12199

Excited State 20: Singlet-A 4.9178 eV 252.11 nm f=0.0116 <S**2>=0.000

112 ->119 -0.13921

113 ->119 0.18155

113 ->120 0.19361

115 ->121 -0.28552

116 ->121 0.31148

117 ->123 0.18060

118 ->124 -0.23084

118 ->125 -0.21966

118 ->126 -0.15187


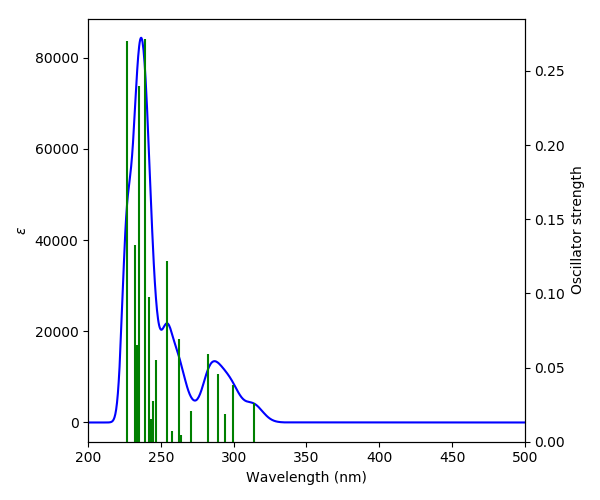

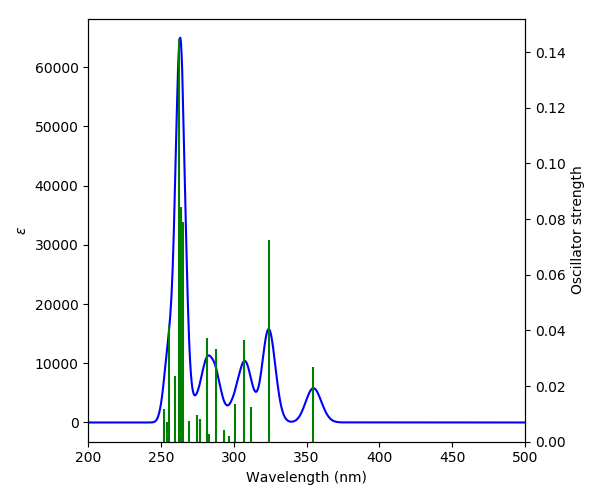


Figure S21: Simulated UV-Vis spectra for (S_P_,*M*)-**1** using TD-DFT (left) and ADC(2) (right)


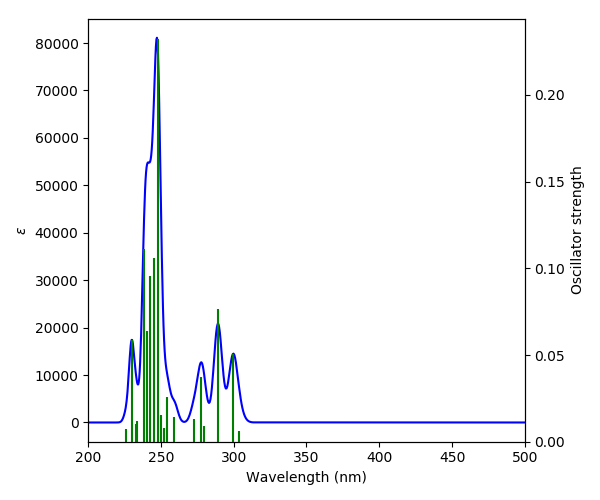

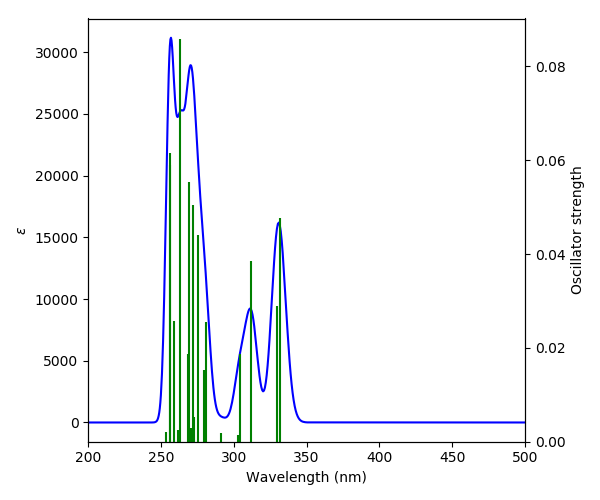


Figure S22: Simulated UV-Vis spectra for (R_P_,*M*)-**1** using TD-DFT (left) and ADC(2) (right)


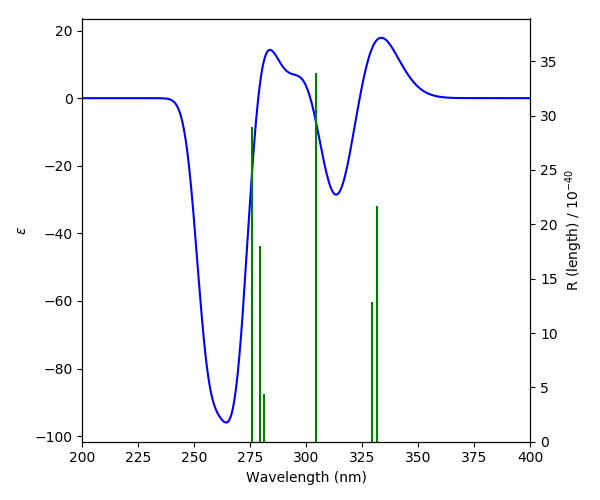

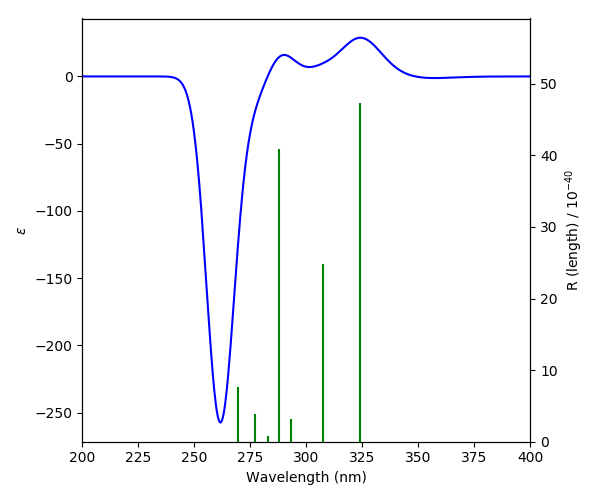


Figure S23: Simulated ECD spectra for (R_P_,*M*)-**1** (left) and (S_P_,*M*)-**1**(right)

1. G. M. Sheldrick*, Acta Cryst*. A71 (2015) 3-8 [↑](#footnote-ref-1)
2. Sheldrick G.M., *Acta Cryst*. C71 (2015) 3-8 [↑](#footnote-ref-2)
3. R. Mokrai, A. Mocanu, M. P. Duffy, T. Vives, E. Caytan, V. Dorcet, T. Roisnel, L. Nyulászi, Z. Benkő, P.-A. Bouit, M. Hissler, *Chem. Commun.* **2021**, *57*, 7256–7259. [↑](#footnote-ref-3)
